# Paintable Carbon Nanotube Coating-Based Textronics for Sustained Holter-Type Electrocardiography

**DOI:** 10.1021/acsanm.2c03904

**Published:** 2022-10-07

**Authors:** Sławomir Boncel, Rafał G. Jędrysiak, Marek Czerw, Anna Kolanowska, Anna W. Blacha, Maciej Imielski, Bertrand Jóźwiak, Marzena H. Dzida, Heather F. Greer, Aleksander Sobotnicki

**Affiliations:** † Faculty of Chemistry, Department of Organic Chemistry, Bioorganic Chemistry and Biotechnology, NanoCarbonGroup, Silesian University of Technology, Krzywoustego 4, 44-100 Gliwice, Poland; ‡ Centre for Organic and Nanohybrid Electronics, Silesian University of Technology, Konarskiego 22B, 44-100 Gliwice, Poland; § Łukasiewicz Research Network Institute of Medical Technology and Equipment, Roosevelta 118, 41-800 Zabrze, Poland; ∥ Department of Biosensors and Processing of Biomedical Signals, Silesian University of Technology, Roosevelta 40, 41-800 Zabrze, Poland; ⊥ Department of Physical Chemistry and Technology of Polymers, Silesian University of Technology, Marcina Strzody 9, 44-100 Gliwice, Poland; # Biotechnology Centre, Silesian University of Technology, Krzywoustego 8, 44-100 Gliwice, Poland; ∇ Department of Chemical Engineering and Process Design, Silesian University of Technology, Marcina Strzody 7, 44-100 Gliwice, Poland; ○ Institute of Chemistry, University of Silesia in Katowice, Szkolna 9, 40-006 Katowice, Poland; ◆ Department of Chemistry, University of Cambridge, Cambridge CB2 1EW, U.K.

**Keywords:** carbon nanotubes, conductive paints, coatings, textronics, electrocardiography

## Abstract

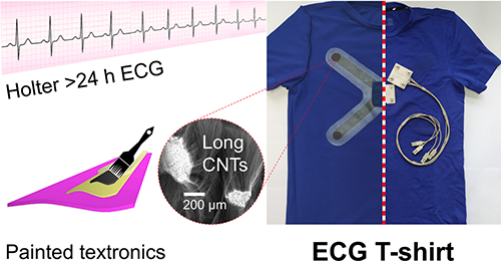

A growing population suffering from or at high risk of developing cardiovascular diseases can benefit from rapid, precise, and readily available diagnostics. Textronics is an interdisciplinary approach for designing and manufacturing high-performance flexible electronics integrated with textiles for various applications, with electrocardiography (ECG) being the most convenient and most frequently used diagnostic technique for textronic solutions. The key challenges that still exist for textronics include expedient manufacturing, adaptation to human subjects, sustained operational stability for Holter-type data acquisition, reproducibility, and compatibility with existing solutions. The present study demonstrates conveniently paintable ECG electroconductive coatings on T-shirts woven from polyester or 70% polyamide and 30% polyester. The up to 600-μm-thick coatings encompass working electrodes of low resistivity 60 Ω sq^–1^ sheathed in the insulated pathways—conjugable with a wireless, multichannel ECG recorder. Long (800 μm) multiwalled carbon nanotubes, with scalable reproducibility and purity (18 g per round of synthesis), constituted the electroactive components and were embedded into a commercially available screen-printing acrylic base. The resulting paint had a viscosity of 0.75 Pa·s at 56 s^–1^ and 25 °C and was conveniently applied using a paintbrush, making this technique accessible to manufacturers. The amplified and nondigitally processed ECG signals were recorded under dry-skin conditions using a certified ECG recorder. The system enabled the collection of ECG signals from two channels, allowing the acquisition of cardiac electrical activity on six ECG leads with quality at par with medical diagnostics. Importantly, the Holter-type ECG allowed ambulatory recording for >24 h under various activities (sitting, sleeping, walking, and running) in three male participants. The ECG signal was stable for >5 cycles of washing, a level of stability not reported yet previously. The developed ECG-textronic application possesses acceptable and reproducible characteristics, making this technology a suitable candidate for further testing in clinical trials.

## Introduction

Textronics encompasses an application-oriented interdisciplinary approach.^1^ It aims to design and manufacture high-performance flexible electronics integrated with textiles.^2^ These complex applications require inspiration from a wide variety of disciplines, such as the physicochemistry of materials, electronics, computer science, automatics, and metrology.^3^ A special area of interest for textronics is biomedicine, where an amalgamation of stimuli-responsive materials with high throughput applicability is desirable.^4^ Several global efforts aim to apply textronics to improve diagnostics, therapeutics, and the rehabilitation of patients. These can potentially improve recovery, reduce mortality, and diminish healthcare costs.^1^

Cardiovascular diseases are the top causes of death globally. Annually, 18 million deaths occur due to cardiovascular diseases, representing 31% of all global deaths (with 85% of them occurring due to strokes and myocardial infarction). It is hence inevitable to address those alarming statistics. Remote textronics could improve health and save lives in aging societies, particularly for older adults living alone. The COVID-19 pandemic has expanded the use of remote diagnostics and theranostics via telemedicine.^5^ Studies conducted during the pandemic revealed that simple, inexpensive, well-established, and noninvasive diagnostic techniques such as electrocardiography (ECG) could substantially reduce mortality in high-risk patients. ECG allows the monitoring of several parameters associated with cardiac pathologies, such as QT interval prolongation, which is associated with fatal arrhythmias and cardiac arrest.^6^ Sultanian et al. found that the pandemic increased mortality in cases of cardiac arrest, warranting intensive monitoring and preventive measures.^7^ Moreover, the prolongation in the QRS interval could be the marker of respiratory failure.^8^ The European Society of Cardiology recommends routine ECG upon initial assessment and even to monitor treatment progress.^9,10^ Studies have shown that continuous ECG monitoring during home management of COVID-19 reduces the risk of major adverse events.^11,12^ Moreover, tracking the oxygen saturation level along with ECG reduces the risk of hospitalization while QRS interval prolongation could be a marker for respiratory failure. This can allow the detection of low levels of oxygen saturation, which is an important parameter indicating severity and progression.^13^ Oxygen saturation below 90% on admission is a strong predictor of patient mortality.^14^ Moreover, a large threat is silent hypoxia that occurs in the absence of dyspnea or tachypnea, resulting in treatment delays and increasing the probability of developing acute respiratory failure.^15^

The core of remote ECG systems is flexible electrical circuits that are conveniently implantable into textiles and electronics via metallic interconnections.^16,17^ This is unlike conventional gel-coated Ag/AgCl electrodes, which show signal quality degradation as the gel dehydrates with prolonged use.^18,19^ Dry electrodes that maintain time-stable electrical contact with the skin can become an alternative to gel electrodes for continuous long-term ECG monitoring. Previously, a shirt with screen-printed silver paste on the fabric and equipped with a low-consumption battery enabled ECG data collection for up to 14 days.^20^ Similarly, scalable GaIn-polymer conductors can be screen-printed on a T-shirt.^21^ Sinha et al. demonstrated the integration of screen-printed ECG circuitry using a commercially available conducting polymer poly(ethylenedioxy–thiophene):poly(styrenesulfonate) onto finished textiles. The polymer-formed electrodes and wires enabled ECG signal recordings comparable to the classical Ag/AgCl electrode connected to copper wires.^22^

In recent years, carbon nanomaterials have become the most promising candidates in textronics owing to their excellent electrical and mechanical properties, low density, and biocompatibility.^19,23,24^ The incorporation of nanomaterials such as graphene^20,25−27^ or carbon nanotubes (CNTs) with various morphologies and surface physicochemistries^28−32^ can improve the electrical performance of flexible electrodes. Gilshteyn et al. prepared electrically conductive hydrogels as skin-like electrodes for wearable electronics.^33^ The incorporation of graphene and oxidized multiwalled carbon nanotubes (MWCNTs) into paper also yielded a flexible electrode.^34^ Additionally, a wireless CNT-based T-shirt for ECG monitoring was designed by Taylor et al.^35^ They constructed electrodes and transmission wires using a thread dip-coated in CNT ink, which was sewn into the T-shirt fabric. In Table 1, we summarized up-to-date ECG technologies based on CNTs and/or their hybrid materials with their key characteristics geared toward high-performance Holter-type monitoring.

**Table 1:** Review of the Up-to-Date ECG Technologies Based on CNTs and/or Their Hybrid Materials^a^

nanocarbon morphology (outer diameter, length, etc.), concentration	base	manufacturing/form of electrodes	electrical properties/electrode thickness	washability	activity upon ECG monitoring	Holter-like performance	ref
MWCNTs (OD = 60 nm, *l* = 0.8 mm), 10 wt %	acrylic	painting/T-shirt	ρ = 60 Ω sq^–1^, *d* = 660 μm	>5 cycles of washing (35 °C), liquid detergent	rest, work, sleep, jogging	>24 h	this work
MWCNTs (OD = 10–40 nm, *l* = 1–25 μm), 1.5 wt %	poly(dimethylsiloxane) (PDMS)	spin coating/chest electrode	σ = 0.09 S m^–1^, *d* = 40 μm, ρ = 2.78 × 10^5^ Ω sq^–1^	no data	rest	no data	(29)
Ag nanoparticles (*D* < 150 nm), oxidized MWCNTs (OD = 20 nm; *l* = 20–100 μm), 1 mg mL^–1^	PDMS	screen-printing/bandage-supported chest and wrist electrode	ρ = 0.1 Ω sq^–1^, *d* = 100 μm	drum washing (40 °C), triple rinsing	rest, moving the arms	no data	(28)
MWCNTs (OD = 10 nm, *l* = 10 mm), graphene flakes (lateral size 4.5 mm, thickness 12 nm), mixed in 1:9 ratio; 1 wt %	PDMS	spin coating/electrode located on stomach, forearm, or ankle	ρ = 150 Ω cm^–1^, *d* = 10–20 μm	washing with water; functioning upon immersion in water	rest, wrist curl flexion and extension, squats, writing	no data	(31)
MWCNTs (OD = 5–10 nm, *l* = 1–25 μm), 1.0–4.5 wt %	PDMS	molded with the acrylic plate/chest electrode	σ = 0.5 × 10^–4^ to 10^1^ S m^–1^, *d* = 3 mm, ρ = 33.33 Ω sq^–1^	no data	treadmill walking (< 5 km h^–1^)	7 days	(32)
graphene and oxidized MWCNTs (OD = 7–80 nm, *l* = 0.5–2 mm) mixed in 6:1 ratio, 25 wt %	*N*,*N*-dimethylformamide/water (*v*/*v* = 1:1)	drop casting on Nylon paper/chest and stomach electrodes	ρ = 75 Ω sq^–1^	no data	rest	no data	(34)
single-walled CNTs (SWCNTs) (*D* = 0.9–3 nm, *l* < 10 μm), no data	alginate polyacrylamide hydrogel	SWCNT film deposited on hydrogel electrode to the arm or the finger	ρ = 100–350 Ω sq^–1^, *d* = 40 nm	no data	rest	no data	(33)
SWCNTs(*D* = 1–3 nm, *l* = 7.4 μm), 0.2 wt %	1 wt % aqueous sodium deoxycholate	dip-coating of the cotton thread/5-electrode T-shirt	σ = 0.01 MS m^–1^, *d* = 240 μm, ρ = 0.42 Ω sq^–1^	tide free, detergent	walking, jogging, and running	no data	(35)
graphene oxide, no data, 50 mg mL^–1^	PDMS-polyurethane/wrist or neck electrode on polyester fabric	dip-coating/skin electrode	σ = 0.216 S m^–1^, *d* = 100 μm, *ρ* = 47,000 Ω sq^–1^	no data	wrist-bending	no data	(27)

a OD—outer diameter (nm); *D*—diameter (nm); *d*—thickness.

Textronic solutions for reliable ECG monitoring have been recently developed that allow convenient manufacturing, adaptation to a variety of human subjects, and sustained stability including washability, reproducibility, and compatibility with existing solutions.^16^ Modifications in the localization, geometry, number of electrodes, and conductive pathways in seamless T-shirts could allow monitoring of other biosignals, such as blood flow, respiratory rate, force exertion, hydration level, and electrolyte balance.^36^ ECG is a technique that allows the conversion of heart-derived biopotentials to electrical signals. ECG—based on the standard Ag/AgCl gel electrodes—allows assessing the functioning of the heart and indicating a variety of cardiovascular diseases.^37^ However, standard Ag/AgCl gel electrodes have several disadvantages, including the induction of dermatitis, discomfort upon application of cold gel, and high cost.^38^ Additionally, using both standard office ECG and long-term ECG (i.e., Holter-type ECG) poses several challenges. These include recording of resting ECG only in the supine position, using up to 12 electrodes, and requiring qualified personnel for treating the patient’s skin. Furthermore, Holter-type ECG electrodes should remain undisturbed for at least 24 h. Additionally, the cables can interfere with patient movement. Lastly, standard electrodes might change conductivity and become breakable upon drying.

Here, we demonstrate as-grown long (L)-MWCNT-based textronics, which are notably a few orders of magnitude less expensive than their SWCNT counterparts, for long-duration Holter-type ECG monitoring. Electrodes and insulated pathways were conveniently and inexpensively painted all-in-one onto a finished polyester or polyamide–polyester T-shirt and integrated with snap fasteners to enable signal output. The circuits in the T-shirts were oriented in various geometries and had flexibility and stretchability matching the human skin. Our technology allowed continuous ECG recording at par with medical diagnostics quality and in various activities performed by different male participants. The outcomes of this work bring us closer to effective and reproducible solutions for rapid medical intervention in remote, outpatient, and hospital settings.

## Materials and Methods

### Materials

Ferrocene (FeCp_2_; 98%, Sigma-Aldrich), toluene (pure, Chempur), sodium dodecyl sulfate (SDS; ≥90%, Sigma-Aldrich), and argon (5.0 UN1006, Siad Poland Ltd.) were used as purchased. Sicotex SX 150 (SICO Screen Inks, Belgium) was used as a transparent acrylic paint base. SDS was used as an auxiliary stabilizer. Deionized water (5 μS cm^–1^) was used as the diluent/solvent.

### Synthesis and Characterization of Long MWCNTs

Long MWCNTs (L-MWCNTs) were synthesized via chemical vapor deposition (CVD) under well-established conditions, and their characteristics were experimentally determined.^39^ Briefly, the reactor served a one-heating-zone (760 °C) STF1200 Tube Furnace (Across International) equipped with a preheater (250 °C) and a syringe pump. The feedstock used was 5.5 wt % of FeCp_2_ (catalyst precursor) in toluene (main carbon source) dosed at a rate of 2.8 mL h^–1^. The flow rate of the carrier gas argon was 1.8 L min^–1^, and the synthesis lasted 24 h. The synthesis emerged as scalable in reproducibility and purity (18 g per synthesis), using up to five synthesis runs per one quartz tube. Scanning electron microscopy (SEM; Phenom Pro Desktop SEM, Thermo Fisher Scientific, Warsaw, Poland) equipped with an energy-dispersive X-ray spectroscopy (EDX) detector (15 kV) was used to acquire the micrographs. Transmission electron microscopy (TEM) was acquired using Thermo Scientific (FEI) Talos F200X G2 TEM at 200 kV with a Ceta 4 k × 4 k CMOS camera. Thermogravimetric analysis (TGA) curves were recorded using a PerkinElmer TGA 8000 thermobalance at a heating rate of 10 °C min^–1^ under both argon and air atmosphere. Raman spectra were acquired with a Renishaw Ramascope1000 spectrometer using a 633-nm-laser with a resolution higher than 1.5 cm^–1^.

### Preparation of the Electroconductive Paint

L-MWCNTs were preincubated using a 5 wt % SDS solution according to the previously published method.^40^ Preindividualized MWCNTs (5 g) were ultrasonicated in 200-mL water using an ultrasonic bath (Bandelin Sonorex Super RK106, 35 kHz, nominal power 480 W) for 30 min. The suspension was stirred using Silverson L5MA (10,000 rpm) and transferred to a cup blender (Bosch SilentMixx). Acrylic base (45 g) was added in portions until the appropriate concentration was achieved. After the addition of all the binders, the resulting slurry was stirred again with a Silverson high-shear mixer for 1 h at maximum speed.

### Rheology of the Electroconductive Paint

The rheological behavior of the paint at 25 °C was measured using spring-type viscosimeter DV2TRV (Brookfield, USA) with a small sample adapter (spindle SC4-28, chamber SC4-13RPY) via two consecutive shear rate ramp tests: up (1–200 rpm, 0.28–56.0 s^–1^) and down (200–1 rpm, 56.0–0.28 s^–1^) with 30-s intervals between each experimental point. Furthermore, the yield stress of the paint was measured using a viscosimeter spring deformation method. The paint was sheared for 30 s at a very low shear rate of 0.28 s^–1^ (1 rpm) before the spindle rotation was stopped. The deformed spring forced the spindle to move back, reducing the torque and shear stress on the spindle surface until it stopped precisely at the yield stress of the sample.^41^ The values of yield stress were determined in triplicate and averaged. The estimated expanded uncertainty of the rheological measurements was ±4.7%.

### Preparation of the Insulated Conductive Pathways

Three layers of Sicotex SX 150 insulation were applied using a brush (No. 16) on a knitted textile substrate (Decathlon or 4F; polyester, 70% polyamide and 30% polyester, and polyurethane Elastan). Upon application, each layer of the insulation was thermoset in a laboratory dryer for 15 min at 105 °C. The conductive paint was applied to the insulating layers multiple times to obtain a satisfactorily low surface resistivity of the conducting path (60 Ω sq^–1^). Each layer of the conducting paint was thermoset in the laboratory dryer for 30 min at 80 °C. The final insulation layers were painted in repeatedly alternating painting and thermosetting until the resistance between the electrode surface and the insulation could not be measured, i.e., >40 MΩ sq^–1^.

### Measurements of the Electrical Properties of the Coatings

A multimeter (UNI-T UT139C with 15.5-mm-diameter brass electrodes) was used for resistance measurements. An electronic universal micrometer (LINEAR 0–25 mm with 6.5-mm diameter probes) was used to measure the thickness of the neat and coated knitwear.

### Measurements of the Mechanical Properties of the Coated T-Shirt Fabric

A static tensile test was performed according to the ISO 13934-1:2013 standard using Zwick Roell Z020. The extension rate was 100 mm min^–1^, and the initial force was 2 N (rate 5 mm min^–1^). The test sample dimensions were 250 mm × 50 mm, and the measurement length was 104 mm. The electrical resistance (measured as above) was monitored upon elongation of the sections of pristine/neat and painted/coated T-shirt fabric. The results are presented as a function of %-elongation. Each measuring point was constructed from three independent measurements.

### Washing Parameters

In the washability studies, fabrics were vigorously agitated in 0.2-wt % SDS aqueous solution. The stirring rate was controlled at 300 rpm, and each washing lasted for 2 h for the first four cycles. For the fifth cycle, the washing time was increased to 12 h. The test was performed on three individual sets of samples.

### ECG Recording

A BlueECG-210 module was used as a wireless device to allow noninvasive cardiological diagnostics of patients of all ages and health statuses. The device had cardiac floating protection for its use in the medical and nonmedical facilities. It was powered by a lithium-polymer battery inaccessible to the user. The device could work continuously for not less than 24 h, record 12-lead ECG signals, and could be used in both rest mode and stress testing. The ECG signals were transmitted in real time via a wireless link to the certified 4HeartsCardiv IT system dedicated to cardiac supervision. The 4HeartsCardiv software was used for the acquisition, visualization, and archiving of ECG signals and biophysical parameters of the patient. These included heart rate (HR), oxygen saturation (SpO_2_), blood pressure, and body temperature. The system enabled the presentation of the electrocardiogram, downloading of examination results from the database, and printing of required parts of the electrocardiogram. A wide-frequency response and unique filtration system guaranteed the accurate presentation of electrocardiograms. Additionally, the system allowed for reliable measurements and analyses as the basis for the interpretation of changes in heart morphology and arrhythmias. HR and ECG measurements (in various arrangements of electrodes and recording systems) were performed for four participants: a 22-year-old male with asthma, a 54-year-old healthy male, a 42-year-old male with diabetes, and a 51-year-old male with cardiovascular disorders). The participants varied in terms of their physiques.

## Results and Discussion

### Synthesis of Highly Vertically Aligned L-MWCNT Array

**Figure 1:**
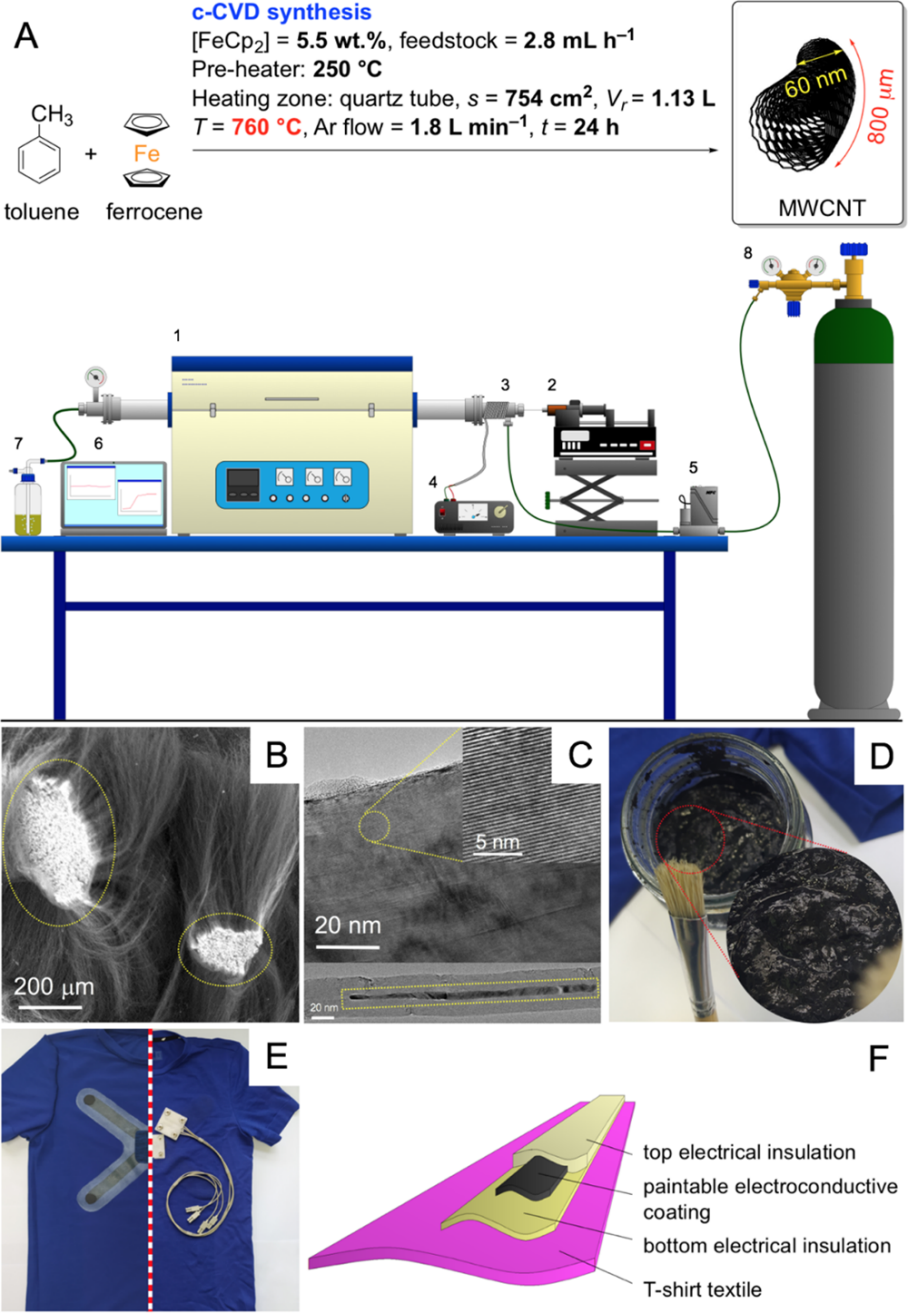
From L-MWCNT to ECG paintable textronics: (A) CVD synthesis of L-MWCNTs: #1 CVD furnace, #2 syringe pump dosing the feedstock, #3 preheater, #4 preheater controller, #5 mass flow controller, #6 CVD furnace controlling unit, #7 oil gas-washing bottle, and #8 carrier gas (Ar) cylinder; (B) SEM and (C) TEM images of L-MWCNTs (insets—magnified areas of multiwalled patterns and iron-based phases); (D) electroconductive paint applicable by a paintbrush (inset—a magnified view showing the homogeneous texture of paint); (E) photo of the optimized ECG T-shirt showing the geometry of the insulated conductive coating with its schematic view (F).

The synthesis process involved embedding highly vertically aligned L-MWCNT arrays (so-called “carpet” or “film”) in the electroconductive coating onto a T-shirt, forming the measuring unit of the functional textronics (Figure 1).

L-MWCNTs served as the key electroconductive component and were synthesized via CVD (Figure 1A). The synthesis was performed in a quartz tube reactor (1) placed in a furnace at a computer-controlled (6) temperature following previously reported protocol but with a longer synthesis time (24 h). This enabled us to achieve 0.8-mm-long MWCNTs, i.e., a high aspect ratio, hence called L-MWCNTs. Briefly, the syringe-dosed toluene solution of FeCp_2_ (2) vaporized in the preheater (3), controllable by the external unit (4), was injected into the stream of argon as the carrier gas (8) regulated by a mass flow controller (5). The exhausts were led out to the fume cupboard via an oil gas-washing bottle (7). The L-MWCNT arrays scrapped with a dedicated rotating head were isolated in the yield *per carbon* equal to 30%. As seen in SEM images (Figure 1B), L-MWCNTs formed fibrous-like superstructures of a high aspect ratio (ca. 15,000). The nanotube bases (encircled with ellipses in Figure 1B) formed the light regions of coalesced (i.e., growing from a “common root”) nanotubes. This fact derives from the higher base (local) concentration of the iron-based phases (α-Fe, γ-Fe, and Fe_3_C).^42^ TEM imaging (Figure 1C) proved that the crystallinity of L-MWCNTs was high, with only randomly dispersed intrusions of amorphous carbon at the surface. This was further confirmed on closer inspection of the magnified images (top inset), revealing characteristic patterning of the multiwalled structure. TEM imaging of the regions of the nanotube bases (a bottom inset) also confirmed that L-MWCNTs contained encapsulated iron phases in the form of the discontinuously filled core. Analysis of the morphology ascertained that the L-MWCNTs were grown via a “base-growth” mechanism, and the long *quasi*-1D nanoparticles could serve as the electroactive component of the functional coating. L-MWCNTs were also synthesized by Aly et al. using chlorine mediated low-pressure CVD and acetylene plus iron(II) chloride as the carbon source and catalyst, respectively.^43^ Their MWCNT array, with an average height of 2 mm and MWCNT diameter of 29 nm, was grown for 20 min at 760 °C. However, the authors did not characterize their grown CNTs.^44−46^ Also, an improved MWCNT purification technique exploiting chlorine led to a large decrease in the amount of metallic impurities.^47^ It should be noted though that when the metallic content decreased to the ppm level, the chlorine concentration in the MWCNT product simultaneously increased to 3 wt %. The release of gaseous HCl upon contact of Cl-containing MWCNTs with water disqualifies such a material from any biomedical applications, apart from the obvious dangers related to handling gaseous chlorine and complications in manufacturing.

The electroconductive paint, applied using a standard paintbrush (Figure 1D), was prepared by mixing L-MWCNTs, water, SDS, and the acrylic base and subjecting the mixture to ultrasonication, high-shear mixing, and blending. The protocol was refined to achieve optimum viscosity and adhesivity of the paint and the final electroconductive coatings embedded in the insulating matrix (Figure 1E,F). The simplicity and availability of our technique are important, especially if more advanced techniques are inaccessible to manufacturers. Other methods to form electrodes include dip- and spin-coating^29^ and screen printing^22,28^ as well as molding electrodes to the desired shape.^31,32^

**Figure 2:**
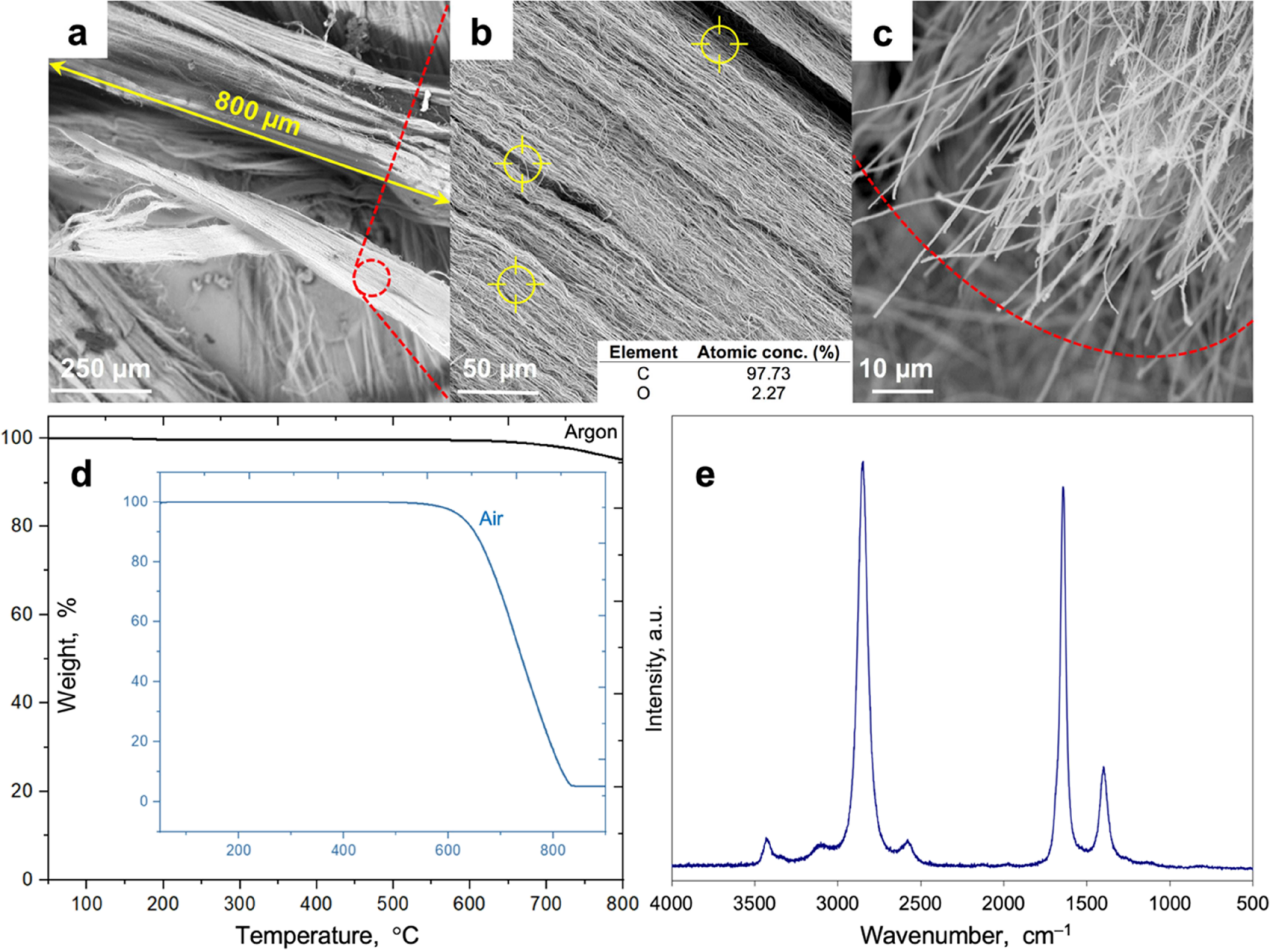
Analysis of the grown MWCNT carpets. SEM images revealing the key morphological features: length of the fibrous-like bundles (a), wavy, aligned fibers (b), and the nanotube tips at the top layer (c); the inset in (b) corresponds to the EDX elemental analysis determined from three points (marked with the viewfinder symbols) with standard deviation lower than 0.25 at %; (d) TGA curves recorded in argon and air; (e) Raman spectrum.

We further characterized our as-grown 800-μm-long MWCNTs (Figure 2) via SEM (Figure 2a–c) and EDX analysis (Figure 2b). MWCNT carpets revealed structural continuity across the entire length of the wavy bundles (Figure 2a,b) (Supporting Information, SI, Figure S1) and were composed purely of carbon, adsorbed water, and encapsulated iron-based nanoparticles undetectable via EDX (Figure 2b).

The as-grown MWCNTs were also analyzed using TGA and Raman spectroscopy. TGA curves were recorded under argon and air, revealing practically full thermal stability up to 800 and ca. 600 °C, respectively. The postcombustion orange solid residue was composed of ca. 5.0 wt % Fe_2_O_3_, which corresponded to 3.5 wt % of total iron in the pristine MWCNT product. The Raman spectrum of MWCNTs shows features typical for graphitic nanomaterials with G-, D-, and 2D-peaks located at 1581, 1354, and 2848 cm^–1^, respectively.^48^ The G-band corresponds to the in-plane stretching of the C–C bonds in the graphene walls. In turn, crystallographic defects in the graphene lattice (Stone–Wales defects, vacancies, adatoms, etc.) are responsible for the emergence of the D-peak, which is a combination of the first order hexagon-breathing mode and an elastic scattering of a photoexcited electron. The *I*_D_/*I*_G_, therefore, constitutes a diagnostic value for determining crystallinity in graphene-like materials. Here, the *I*_D_/*I*_G_ ratio of 0.28 can be attributed to a relatively low number of defects. In turn, the 2D-band is attributable to breathing vibrations of the six carbon atoms in the single unit of the super-conjugated “benzene-like” lattice^49^ while its relatively high intensity could be ascribed to the abundant incommensurate graphene walls originating from the nanotube waviness.^50^ The 2D satellite peaks at 2580 and 3103 cm^–1^ conform to the multiwall structure in the relatively thick MWCNTs.^48^ Overall, the above characteristics confirm that MWCNTs are wavy/curly at the macroscale and dominantly crystalline in the nanoscale.

A careful analysis of the micromorphology and length distribution of MWCNTs embedded into the acrylic paint base before curing and during manufacturing revealed significant changes (Figure S2). Curing itself changed neither the morphology nor the length distribution. The mean length of the cut MWCNT bundles calculated from optical micrographs of 150 MWCNT bundles was 31 ± 21 μm (mean ± standard deviation; median = 28 μm) with the minimal and maximal lengths of 3 and 102 μm, respectively.

### Rheological Properties of L-MWCNT-Based Paints

From a rheological point-of-view, the paint was optimized by targeting its yield stress in the range of 1–10 Pa. This range was a trade-off between high shelf life stability, good leveling or sag resistance, and easy distribution on the surface.^51,52^ The results were obtained as a function of nanotube concentration, as shown in Table 2. We decided that the optimum paint had yield stress approximately in the middle of this interval, i.e., 6.44 ± 0.10 Pa.

**Table 2:** Yield Stress of Paint at 25 °C at Various MWCNT Concentrations

MWCNT concentration (wt %)	yield stress (Pa)
0.2	0.13 ± 0.03
0.5	0.37 ± 0.03
1	1.76 ± 0.20
2	6.44 ± 0.10^a^
3	b

a Determined as the optimum.

b High-viscosity “bucky gel”—results outside the measuring range of the viscometer.

**Figure 3:**
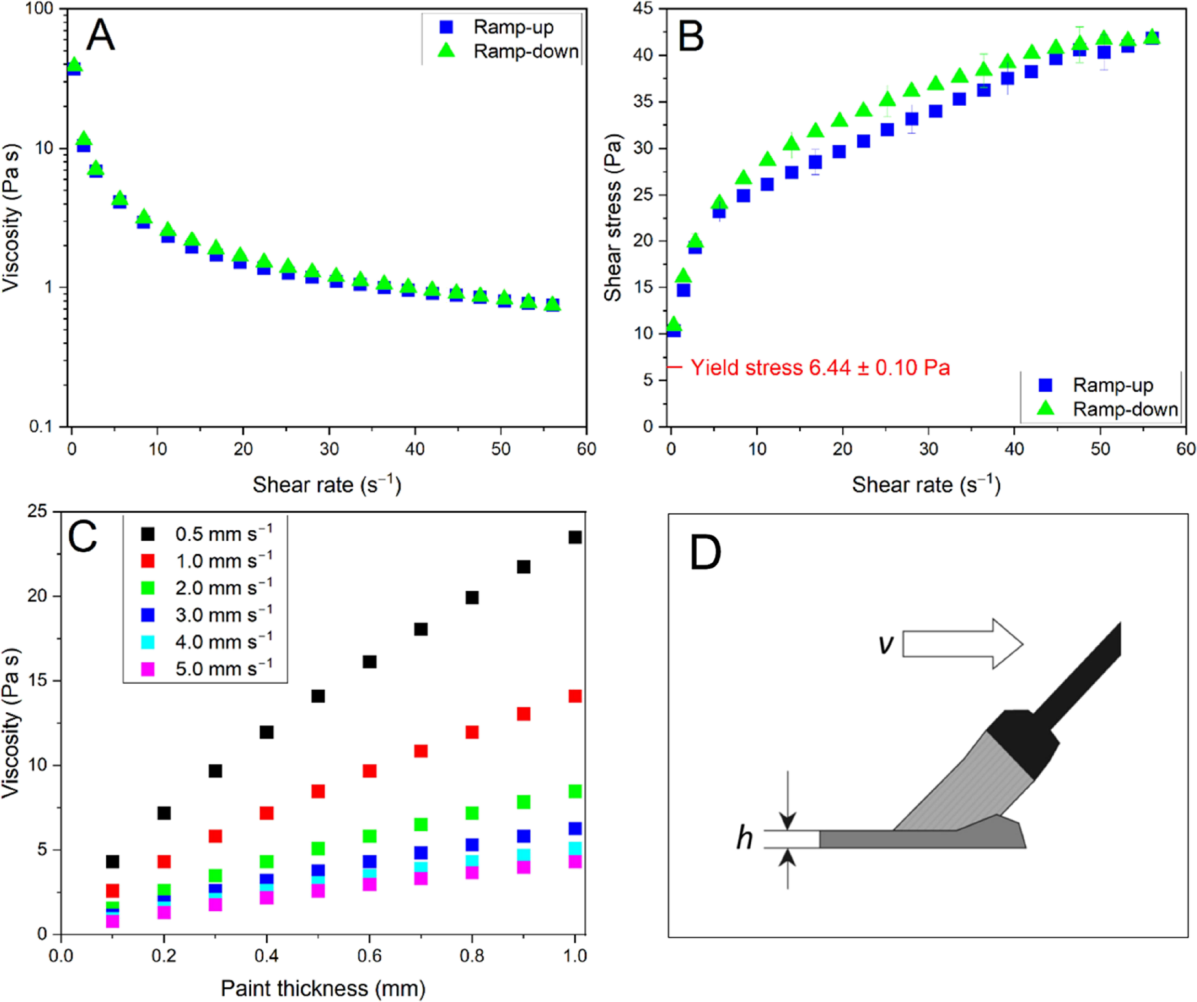
Rheological behavior of the selected paint at 25 °C: (A) viscosity as a function of shear rate, (B) shear stress as a function of shear rate with yield stress marked in red, (C) viscosity as a function of wet paint thickness and painting/brushing speed. (D) Schematic of the painting with the indicated speed of the painting (*v*) and the thickness of the wet layer (*h*).

Detailed rheological studies of the selected paint (Figure 3) at 25 °C revealed that the paint was a shear-thinning non-Newtonian fluid and its viscosity decreased from 39.0 Pa·s at 0.28 s^–1^ to 0.75 Pa·s at 56 s^–1^ (Figure 3A). This behavior was due to the breakdown of carbon agglomerates and the alignment of nanoparticles along the flow direction, leading to a reduction of internal friction in the fluid.^53^ Shear-thinning was a very desirable and useful phenomenon, which ensured that the paint was easy to mix, pump, and apply to surfaces (high/medium shear rates → low viscosity). This also indicated good stability during storage and drying (low shear rates → high viscosity).^51^ At 25 °C, the paint exhibited significant yield stress of 6.44 ± 0.10 Pa, which represents the minimum stress necessary to initiate irreversible deformation/flow. High yield stress can hinder the flow of coating under gravity, giving better shelf life stability and sag resistance.^51,52^ Furthermore, the paint in the analyzed shear rate range and temperature revealed slight, although noticeable, time-dependent antithixotropic properties, as indicated by the hysteresis loop (Figure 3B). At a constant shear rate, the longer the fluid is deformed, the greater its shear stress and viscosity become due to a reversible shear-induced build-up of network structure.^54,55^

The rheological behavior of the selected paint strongly depended on the manner of its distribution (Figure 3C). The shear rate (γ̇) during the painting process can be expressed as
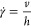
where *v* is the speed of painting and *h* is the thickness of the wet layer (Figure 3D). The increased thickness of paint (at constant painting speed) resulted in a lower shear rate. This led to a higher apparent viscosity because the paint was a shear-thinning non-Newtonian fluid (Figure 3A). A higher viscosity in turn effectively reduced the flow of paint.

### High-Performance L-MWCNT-Based Electroconductive Coatings toward ECG: From Fitness to Medical Quality Diagnostics

**Figure 4:**
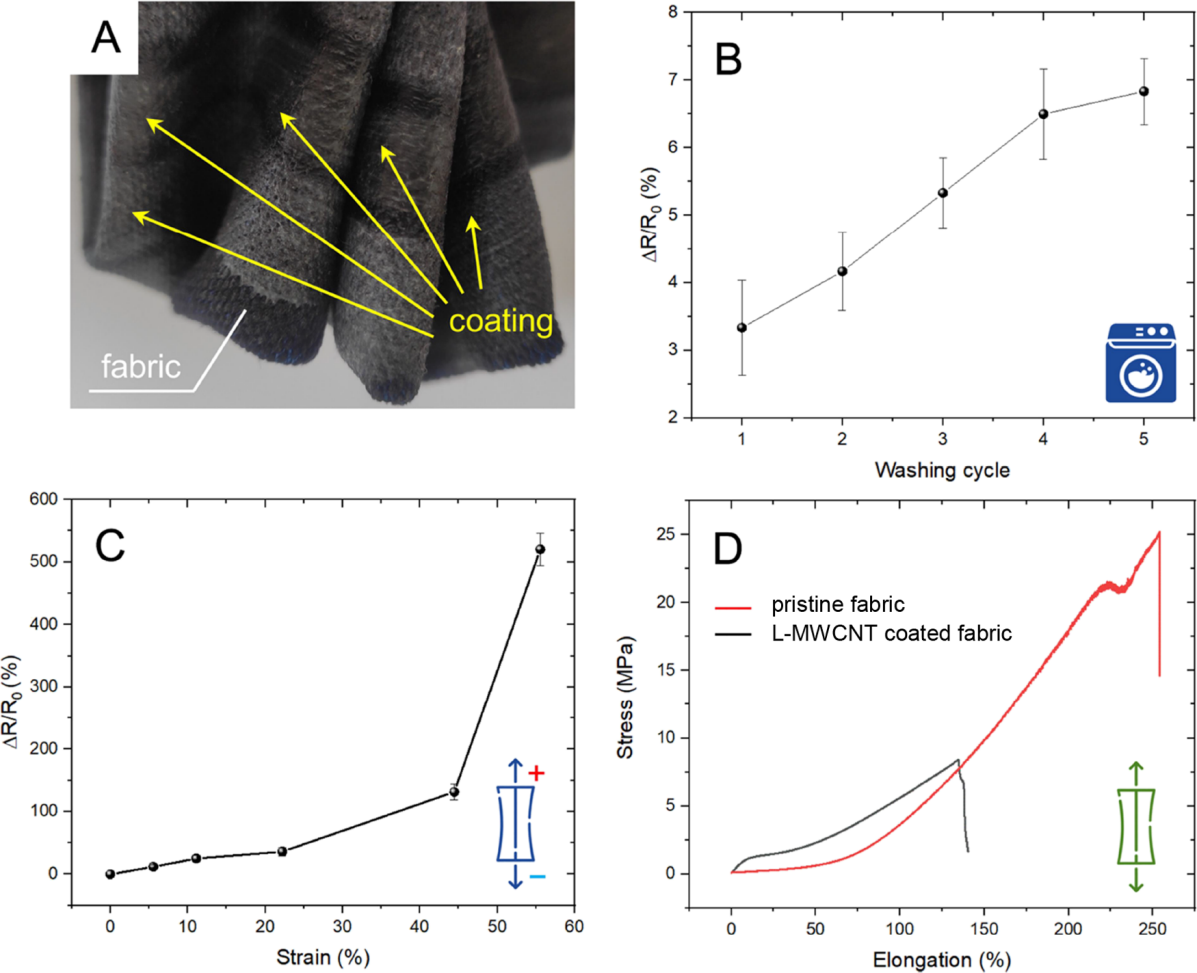
(A) Photograph of L-MWCNT-coated ECG textile demonstrating its flexibility at the coatings edge. Durability of the coating upon subsequent cycles of (B) washing and drying (C) and strain—both shown as changes in the relative resistance of the coating. (D) Stress–strain curves of the pristine and coated ECG T-shirt textile.

**Figure 5:**
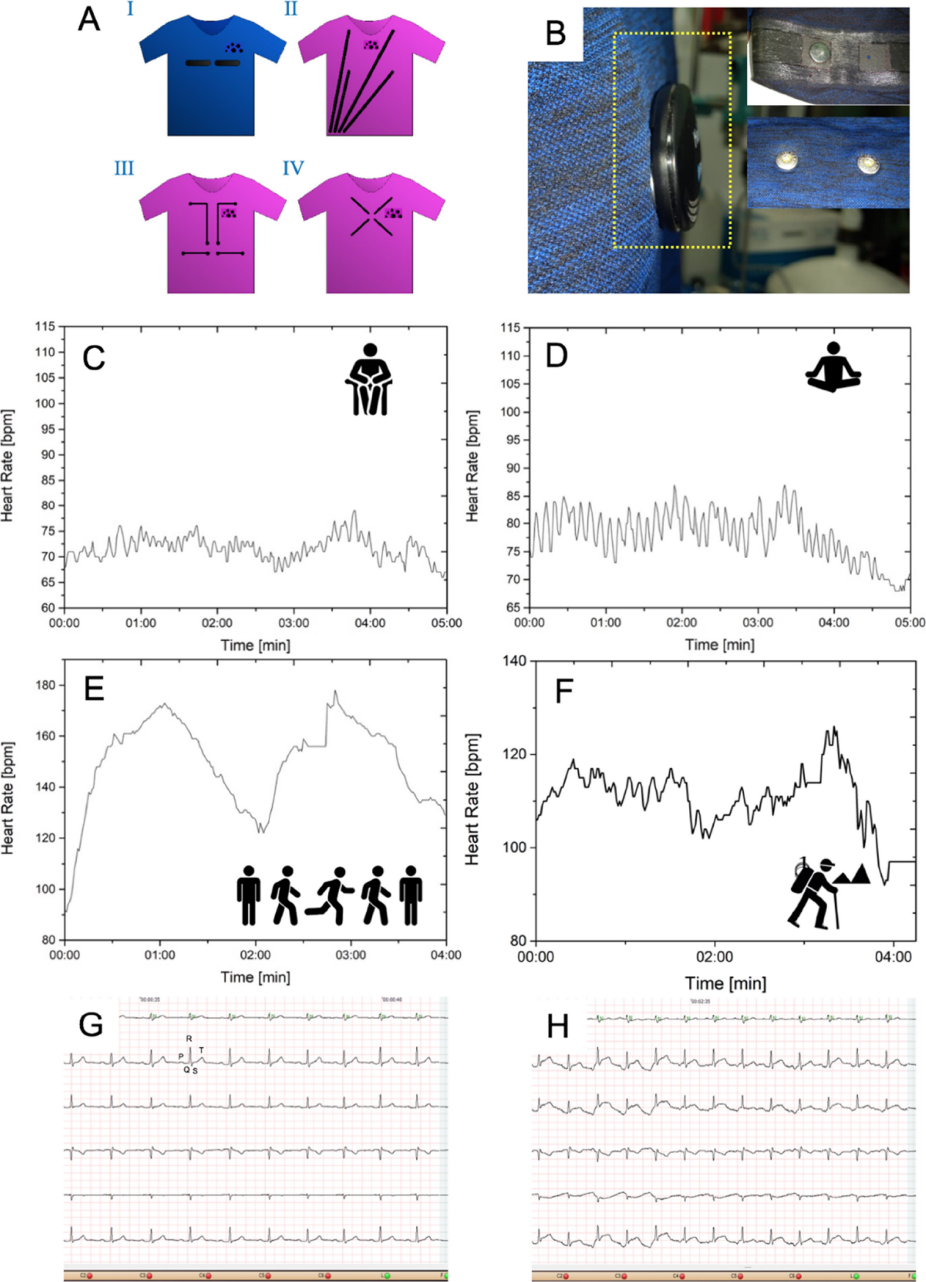
(A) T-shirts coated with different geometries of the insulated electroconductive paths of paintable wiring enclosed in polymer sheaths: (I) fitness system compatible with a commercially available heart monitoring gadget (Decathlon), (II) first long-term Holter-type monitoring system compatible with a commercially available ECG monitoring system (Emtel),(III) preoptimized system enabling long-term Holter-like monitoring in the person wearing the T-shirt and monitoring of ECG signals generated artificially, (IV) final demonstration version of the medical-grade T-shirt for males with the optimized geometry including equalized resistance of pathways. (B) Photograph of the HR measuring device clipped onto the snaps in the T-shirt; insets show the T-shirt bottom (the first snap *implemented* and the second snap *to be implemented*) and top (*two snaps yet to be implemented*) with the outlets connected to the measuring device. (C–F) HR of a healthy, 22-year-old male participant recorded using the final demonstration version IV during: (C) normal breathing while seated; (D) slow, deep breathing while seated; (E) interval running; (F) walking outdoors; (G) a sample of two-channel ECG registration during calm activity in a 22-year-old male participant (showing the PQRST complex), HR = 75; and (H) a sample of two-channel ECG registration of a 51-year-old male participant while walking, HR = 96.

We optimized the geometries to ensure uniformity of conductivity of the paintable electrical pathways, simplification of technology, economy of the materials and technology, higher medical diagnostic value of the received signals, and complex variation in end-users. Thus, we tested the performance of the coatings upon strain and washing (Figure 4) and designed four different 2D arrangements of pathway-electrode coatings (Figure 5).

From a mechanical property point-of-view, the coated ECG T-shirt fabric was fully flexible (Figure 4A). The decrease in tensile strength after coating was related to higher rigidity as the paint penetrated the fabric fibers (Figure 4D). Simultaneously, the fabric coating with low resistivity (60 Ω sq^–1^) emerged as resistant to washing and strain, with Δ*R*/*R*_0_ not exceeding 7% after five cycles of washing/drying (Figure 4B). This level of resistance has not been reported yet and could allow high-quality ECG recordings. Furthermore, Δ*R*/*R*_0_ showed a 125% increase upon 45% elongation (Figure 4C). This amount of elongation far exceeds real-life strain that can be encountered during ECG signal acquisition.

The initial geometric arrangement of the textronic connections (Figure 5A, II) enabled the universal recording of ECG signals regardless of the recorder system connected or the physique of the person wearing the shirt (S, XS, and L sizes, according to the International Men’s Clothing Sizes). Moreover, we designed our demonstration version (Figure 5A, IV) to allow personalization of chest size and was adapted to a next-generation recorder that has two-channel ECG and respiratory wave recording capacities. We developed textronic connections with reduced lengths and designed a new mechanical structure accommodating the recorder, ensuring stable mounting of the recorder module and reproducibility of recordings in real-world conditions. The external dimensions of the signal recorder were reduced so that they do not exceed half the size of a credit card. This enabled user-friendly integration with textronic T-shirts and reduced overall susceptibility to artifacts. Our approach is different from other methods in which whole ECG monitoring systems were composed of CNT-based electrodes, metal wiring,^25,33^ or printed silver inks.^30^ Such approaches required additional supporting materials or bandages.^28,29^ Moreover, the wires can cause skin irritation and discomfort, particularly during long-term ECG recording.^56^ Herein, the T-shirt we designed is a unified system composed of both electrodes and insulated transmission with no wires. The integration of the snap fasteners enabled the transfer of signals to the recording device. The ECG T-shirt was also as flexible as a sports T-shirt, and the electrodes did not cause discomfort to the human skin. Furthermore, we used versatile, scalable, and inexpensive materials and reagents while achieving high electronic performance and excellent operational stability.

We tested the capacity of the ECG T-shirt to monitor HR starting at a fitness-level quality of diagnostics (Figure 5A, I). In this case, it was necessary to design various arrangements of the two electrodes and a commercially available device (Figure 5B). The positioning of the electrodes was inspired by the commercially available Decathlon Kalenji jersey and a Polar HR monitoring belt. The devices were intended to work during cycling for a minimum of 4 h. To acquire the HR signals derived from the L-MWCNT coatings, a heartbeat Polar H10 belt and the Decathlon Coach application were used with the Prym 4GB steel snaps as the electrical connectors. As the original electrode snap connection was hardly generating reproducible results, the connectors were reinforced with knitwear pieces. During the first test (Figure 5C), the HR signal was collected while seated and breathing normally. The signal was continuous and in the range of normal HR in the individual wearing the T-shirt. The second HR data (Figure 5D) were collected while seated and making slow and deep breaths. It should be emphasized that, after further optimization, such T-shirts could be optimized for monitoring breathing cycles in patients with sleep apnea. The next test (Figure 5E) was performed during more intense physical activity. The person wearing the T-shirt was running for 1 min and then standing still for 1 min, and two such cycles were registered. During the second cycle, there was a fragment of the HR curve with a flat course followed by a sudden increase. This could be due to limited contact between the electrode and the individual’s skin for a short period. Overall, the T-shirt equipped with paintable coatings allowed HR monitoring at both low and high intensities of physical activity. Importantly, the same system allowed HR monitoring upon taking a stroll outdoors (Figure 5F) and recording full ECG traces (Figure 5G,H). The ECG signals for two channels and six ECG leads were recorded with the certified BlueECG-210 ECG module. This is a component of the Cardiv system for standard resting and stress ECG examinations. We evaluated a demonstration version of the medical-grade T-shirt with the optimized geometry, including resistance-compensated tracks (Figure 5A, IV). Figure 5G,H shows the ECG recording of a healthy 51-year-old male during the resting and exercise tests (fast walking pace), respectively. In the resting ECG test (Figure 5G), the ECG signal was standard with suitable diagnostic quality, high amplitude (>1 mV), negligible noise, and stable, averaged PQRST complex, i.e., the electrical signals in a single cardiac cycle.^24^ For the stress test (Figure 5H), the ECG signal was sufficient for analysis and calculation of HR and the PQRST complex was undistorted. In both cases, the recorded signals did not differ in quality from the classical exercise test that uses Ag/AgCl gel electrodes.

### Sustained >24-h-Long Holter-Type ECG T-Shirt

Arrangement II (Figure 5, A) was the initial geometry selected for long-term Holter ECG monitoring of a 42-year-old male with diabetes. The experiment lasted 24 h and ECG signals were continuously recorded during normal working day activities (i.e., chemist working in a synthetic laboratory and an office) and during sleep (Figure 6). Detailed recording intervals can be found in the Supplementary Information (Figures S3–S19).

**Figure 6:**
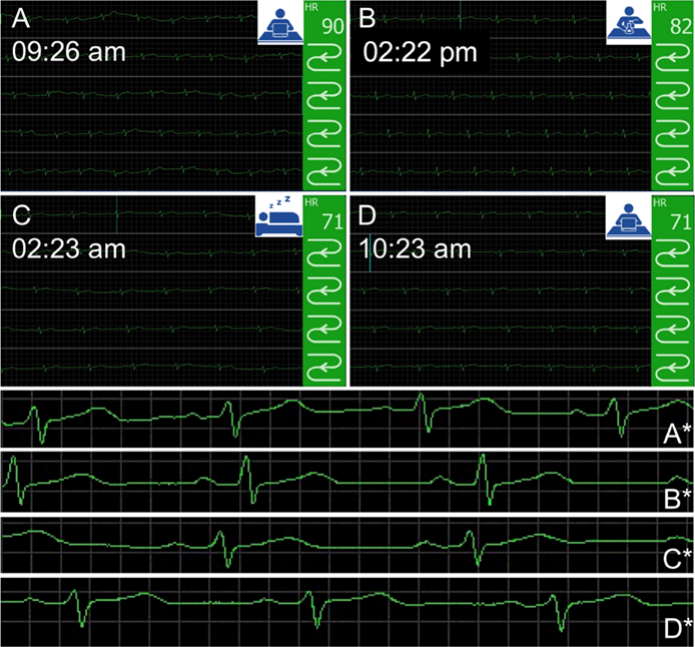
ECG signals sectioned and recorded at different periods of day and night of a 42-year-old male patient with diabetes: (A) 09:26 AM; (B) 2:22 PM; (C) 02:23 AM; (D) 10:23 AM; A*–D*—the corresponding magnified areas of ECG signals displaying the well-defined PQRST complexes.

The signals were collected and recorded on an EMTEL bedside monitor and transmitted wirelessly via Bluetooth. In addition to the ECG signal, the HR was displayed. The electrodes did not require wetting before or throughout the whole experiment. ECG recordings of diagnostic value were available when the patient wore the T-shirt (Figure 6A). The quality of the signal remained at this level until 12:21 PM, when a disturbance in the complex PQRST waveform was observed. This could have most likely resulted from more intense body movements. Nevertheless, the curve immediately returned to the medical level quality, allowing observation of both HR and the PQRST waveform. Indeed, physical activity from 09:20 AM to 12:20 PM was limited to working at a computer. From 01:00 PM to 4:20 PM, the recording also allowed monitoring of the HR and the PQRST waveform (Figure 6B). It was a period of slightly more intense physical activity, i.e., working in a standing position. The recording continued smoothly and stably even after the patient fell asleep (Figure 6C). Eventually, the ECG tracing returned to a pattern similar to the recordings obtained at the beginning of the previous day, with an increased level of physical activity upon paperwork (Figure 6D).

**Figure 7:**
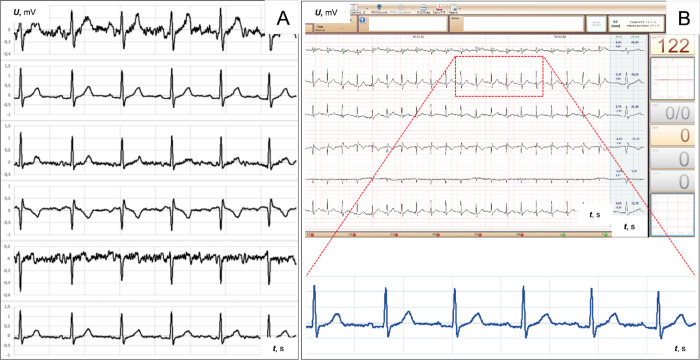
Six-lead ECG signals from the optimized version of the medical grade T-shirt using arrangement IV (from Figure 5), which was recorded during the stress test: (A) initial ECG tracing; (B) ECG tracing after 3 min of walking.

We achieved technological optimization with the final arrangements (III) and (IV) (Figure 5A), i.e., minimal material costs and standardization of the electrode geometry. We minimized the differences in electroconductivity, allowing temporally stable ECG recordings (Figure 7).

A cardiac exercise stress test was performed on a healthy 51-year-old male during which HR and ECG were monitored. With the increase in training intensity after 3 min, HR increased to 122 min^–1^ from a baseline HR of 115 min^–1^. These results show the diagnostic quality of the recorded electrocardiogram during the stress test. The noise level was found to be independent of the intensity of physical activity. Temporary fluctuations of the basic ECG signal (Figure 7A) did not affect the diagnostic value and the correct interpretation of the signal characteristics. The calculated averaged PQRST complexes were stable, unambiguous, and undisturbed at all times, further confirming the high diagnostic value of the recorded ECG signals. Furthermore, as the HR increased, the stability of the ECG signal improved, which was likely due to enhanced skin contact of the T-shirt dry electrodes (Figure 7B). Concerning the safety of this technology, no skin allergic reactions were observed in all four participants during HR monitoring and >24-h-long ECG recording. This corresponds well with previous reports that found negligible toxicity of CNTs to human skin. For instance, Huczko and Lange performed clinical studies that determined whether CNTs induce allergic reactions and skin irritation. In their sample of 40 volunteers, there were no reports of irritation and allergic reaction after 96 h.^57^ Other studies showed that MWCNTs were nonirritating to the skin in vivo and nonirritating to the eye and skin rabbit cells in the in vitro studies.^58^ Additionally, Kumar et al. performed a simple leach test on their acrylic-based CNT electrodes for ECG monitoring. Their study revealed that the amount of CNTs transferrable to a wearer’s skin was negligible.^59^ Indeed, the quantities tested in these safety studies are insignificant compared to quantities used in studies evaluating the cytotoxicity of CNTs.^60^

Detailed parameter variations during the preparation of the functional T-shirt can be found in the Supplementary Information (Figures S20 and S21). Detailed information comparing the ECG T-shirts to the conventional Ag/AgCl electrodes (Figure S32), including their stepwise manufacturing at all stages, is included in Figures S22–S31 and S33–S50.

## Conclusions

We have developed a convenient textronic system that enables prolonged (>24 h) Holter-type ECG recordings. The all-in-one system (electrodes, transmission, and insulation) was easily achievable by applying L-MWCNT-based paint on a T-shirt base. The system represents a versatile tool in remote medicine that can be easily utilized for applications such as monitoring and intervention for soldiers, firefighters, and other professionals in which remote tracking of health is critical. The system can also be easily adapted for automated alert systems upon remote ECG signal processing in rehabilitation supervision. The list of possible applications remains open, with this technology possibly finding translational use in flexible radar absorbing materials or electromagnetic interference shielding.

## Supporting Information Available

SEM images of L-MWCNTs, optical micrographs of MWCNTs embedded in the paint, detailed Holter-type data (i.e., ECG and HR recordings), and documentation of the T-shirt textronics (PDF)

## References

[ref1] Choudhry N. A.; Arnold L.; Rasheed A.; Khan I. A.; Wang L. Textronics—A Review of Textile-Based Wearable Electronics. Adv. Eng. Mater. 2021, 23, 2100469 10.1002/adem.202100469.

[ref2] Angelucci A.; Cavicchioli M.; Cintorrino I. A.; Lauricella G.; Rossi C.; Strati S.; Aliverti A. Smart Textiles and Sensorized Garments for Physiological Monitoring: A Review of Available Solutions and Techniques. Sensors 2021, 21, 814 10.3390/s21030814. 33530403 PMC7865961

[ref3] Liman M. L. R.; Islam M. T. Emerging Washable Textronics for Imminent E-Waste Mitigation: Strategies, Reliability, and Perspective. J. Mater. Chem. A 2022, 2697 10.1039/D1TA09384C.

[ref4] Shim H. J.; Sunwoo S.-H.; Kim Y.; Koo J. H.; Kim D.-H. Functionalized Elastomers for Intrinsically Soft and Biointegrated Electronics. Adv. Healthcare Mater. 2021, 10, 2002105 10.1002/adhm.202002105. 33506654

[ref5] Ivanoska-Dacikj A.; Stachewicz U. Smart Textiles and Wearable Technologies – Opportunities Offered in the Fight against Pandemics in Relation to Current COVID-19 State. Rev. Adv. Mater. Sci. 2020, 59, 487–505. 10.1515/rams-2020-0048.

[ref6] Long B.; Brady W. J.; Bridwell R. E.; Ramzy M.; Montrief T.; Singh M.; Gottlieb M. Electrocardiographic Manifestations of COVID-19. Am. J. Emerg. Med. 2021, 41, 96–103. 10.1016/j.ajem.2020.12.060. 33412365 PMC7771377

[ref7] Sultanian P.; Lundgren P.; Strömsöe A.; Aune S.; Bergström G.; Hagberg E.; Hollenberg J.; Lindqvist J.; Djärv T.; Castelheim A.; Thorén A.; Hessulf F.; Svensson L.; Claesson A.; Friberg H.; Nordberg P.; Omerovic E.; Rosengren A.; Herlitz J.; Rawshani A. Cardiac Arrest in COVID-19: Characteristics and Outcomes of in- and out-of-Hospital Cardiac Arrest. A Report from the Swedish Registry for Cardiopulmonary Resuscitation. Eur. Heart J. 2021, 42, 1094–1106. 10.1093/eurheartj/ehaa1067. 33543259 PMC7928992

[ref8] Alareedh M.; Nafakhi H.; Shaghee F.; Nafakhi A. Electrocardiographic Markers of Increased Risk of Sudden Cardiac Death in Patients with COVID-19 Pneumonia. Ann. Noninvasive Electrocardiol. 2021, 26, e12824 10.1111/anec.12824. 33463863 PMC7995115

[ref9] Baigent C.; Windecker S.; Andreini D.; Arbelo E.; Barbato E.; Bartorelli A. L.; Baumbach A.; Behr E. R.; Berti S.; Bueno H.; Capodanno D.; Cappato R.; Chieffo A.; Collet J.-P.; Cuisset T.; de Simone G.; Delgado V.; Dendale P.; Dudek D.; Edvardsen T.; Elvan A.; González-Juanatey J. R.; Gori M.; Grobbee D.; Guzik T. J.; Halvorsen S.; Haude M.; Heidbuchel H.; Hindricks G.; Ibanez B.; Karam N.; Katus H.; Klok F. A.; Konstantinides S. V.; Landmesser U.; Leclercq C.; Leonardi S.; Lettino M.; Marenzi G.; Mauri J.; Metra M.; Morici N.; Mueller C.; Petronio A. S.; Polovina M. M.; Potpara T.; Praz F.; Prendergast B.; Prescott E.; Price S.; Pruszczyk P.; Rodríguez-Leor O.; Roffi M.; Romaguera R.; Rosenkranz S.; Sarkozy A.; Scherrenberg M.; Seferovic P.; Senni M.; Spera F. R.; Stefanini G.; Thiele H.; Tomasoni D.; Torracca L.; Touyz R. M.; Wilde A. A.; Williams B. European Society of Cardiology Guidance for the Diagnosis and Management of Cardiovascular Disease during the COVID-19 Pandemic: Part 1—Epidemiology, Pathophysiology, and Diagnosis. Eur. Heart J. 2022, 43, 1033–1058. 10.1093/eurheartj/ehab696. 34791157 PMC8690026

[ref10] Baigent C.; Windecker S.; Andreini D.; Arbelo E.; Barbato E.; Bartorelli A. L.; Baumbach A.; Behr E. R.; Berti S.; Bueno H.; Capodanno D.; Cappato R.; Chieffo A.; Collet J.-P.; Cuisset T.; de Simone G.; Delgado V.; Dendale P.; Dudek D.; Edvardsen T.; Elvan A.; González-Juanatey J. R.; Gori M.; Grobbee D.; Guzik T. J.; Halvorsen S.; Haude M.; Heidbuchel H.; Hindricks G.; Ibanez B.; Karam N.; Katus H.; Klok F. A.; Konstantinides S. V.; Landmesser U.; Leclercq C.; Leonardi S.; Lettino M.; Marenzi G.; Mauri J.; Metra M.; Morici N.; Mueller C.; Petronio A. S.; Polovina M. M.; Potpara T.; Praz F.; Prendergast B.; Prescott E.; Price S.; Pruszczyk P.; Rodríguez-Leor O.; Roffi M.; Romaguera R.; Rosenkranz S.; Sarkozy A.; Scherrenberg M.; Seferovic P.; Senni M.; Spera F. R.; Stefanini G.; Thiele H.; Tomasoni D.; Torracca L.; Touyz R. M.; Wilde A. A.; Williams B. ESC Guidance for the Diagnosis and Management of Cardiovascular Disease during the COVID-19 Pandemic: Part 2—Care Pathways, Treatment, and Follow-Up. Eur. Heart J. 2022, 43, 1059–1103. 10.1093/eurheartj/ehab697. 34791154 PMC8690006

[ref11] Bergamaschi L.; D’Angelo E. C.; Paolisso P.; Toniolo S.; Fabrizio M.; Angeli F.; Donati F.; Magnani I.; Rinaldi A.; Bartoli L.; Chiti C.; Biffi M.; Pizzi C.; Viale P.; Galié N. The Value of ECG Changes in Risk Stratification of COVID-19 Patients. Ann. Noninvasive Electrocardiol. 2021, 26, e12815 10.1111/anec.12815. 33512742 PMC7994985

[ref12] Kaliyaperumal D.; Bhargavi K.; Ramaraju K.; Nair K. S.; Ramalingam S.; Alagesan M. Electrocardiographic Changes in COVID-19 Patients: A Hospital-Based Descriptive Study. Indian J. Crit. Care Med. 2022, 26, 43–48. 10.5005/jp-journals-10071-24045. 35110843 PMC8783240

[ref13] Osman A. M.; Farouk S.; Osman N. M.; Abdrabou A. M. Longitudinal Assessment of Chest Computerized Tomography and Oxygen Saturation for Patients with COVID-19. Egypt. J. Radiol. Nucl. Med. 2020, 51, 255 10.1186/s43055-020-00376-y.

[ref14] Mejía F.; Medina C.; Cornejo E.; Morello E.; Vásquez S.; Alave J.; Schwalb A.; Málaga G. Oxygen Saturation as a Predictor of Mortality in Hospitalized Adult Patients with COVID-19 in a Public Hospital in Lima, Peru. PLoS One 2020, 15, e0244171 10.1371/journal.pone.0244171. 33370364 PMC7769479

[ref15] Van Son C. R.; Eti D. U. Screening for COVID-19 in Older Adults: Pulse Oximeter vs. Temperature. Front. Med. 2021, 8, 660886 10.3389/fmed.2021.660886. PMC8079646 33937297

[ref16] Chen S.; Qi J.; Fan S.; Qiao Z.; Yeo J. C.; Lim C. T. Flexible Wearable Sensors for Cardiovascular Health Monitoring. Adv. Healthcare Mater. 2021, 10, 2100116 10.1002/adhm.202100116. 33960133

[ref17] Nigusse A. B.; Mengistie D. A.; Malengier B.; Tseghai G. B.; Langenhove L. V. Wearable Smart Textiles for Long-Term Electrocardiography Monitoring—A Review. Sensors 2021, 21, 4174 10.3390/s21124174. 34204577 PMC8234162

[ref18] Searle A.; Kirkup L. A Direct Comparison of Wet, Dry and Insulating Bioelectric Recording Electrodes. Physiol. Meas. 2000, 21, 271–283. 10.1088/0967-3334/21/2/307. 10847194

[ref19] Llerena Zambrano B.; Renz A. F.; Ruff T.; Lienemann S.; Tybrandt K.; Vörös J.; Lee J. Soft Electronics Based on Stretchable and Conductive Nanocomposites for Biomedical Applications. Adv. Healthcare Mater. 2021, 10, 2001397 10.1002/adhm.202001397. 33205564

[ref20] Yoo J.; Yan L.; Lee S.; Kim H.; Yoo H.-J. A Wearable ECG Acquisition System With Compact Planar-Fashionable Circuit Board-Based Shirt. IEEE Trans. Inform. Technol. Biomed. 2009, 13, 897–902. 10.1109/TITB.2009.2033053. 19789119

[ref21] Tang L.; Mou L.; Zhang W.; Jiang X. Large-Scale Fabrication of Highly Elastic Conductors on a Broad Range of Surfaces. ACS Appl. Mater. Interfaces 2019, 11, 7138–7147. 10.1021/acsami.8b20460. 30681826

[ref22] Sinha S. K.; Noh Y.; Reljin N.; Treich G. M.; Hajeb-Mohammadalipour S.; Guo Y.; Chon K. H.; Sotzing G. A. Screen-Printed PEDOT:PSS Electrodes on Commercial Finished Textiles for Electrocardiography. ACS Appl. Mater. Interfaces 2017, 9, 37524–37528. 10.1021/acsami.7b09954. 29020777

[ref23] Yao S.; Swetha P.; Zhu Y. Nanomaterial-Enabled Wearable Sensors for Healthcare. Adv. Healthcare Mater. 2018, 7, 1700889 10.1002/adhm.201700889. 29193793

[ref24] Kolanowska A.; Herman A. P.; Jędrysiak R. G.; Boncel S. Carbon Nanotube Materials for Electrocardiography. RSC Adv. 2021, 11, 3020–3042. 10.1039/D0RA08679G. 35424207 PMC8693996

[ref25] Dallinger A.; Keller K.; Fitzek H.; Greco F. Stretchable and Skin-Conformable Conductors Based on Polyurethane/Laser-Induced Graphene. ACS Appl. Mater. Interfaces 2020, 12, 19855–19865. 10.1021/acsami.0c03148. 32249561 PMC7304821

[ref26] Du X.; Jiang W.; Zhang Y.; Qiu J.; Zhao Y.; Tan Q.; Qi S.; Ye G.; Zhang W.; Liu N. Transparent and Stretchable Graphene Electrode by Intercalation Doping for Epidermal Electrophysiology. ACS Appl. Mater. Interfaces 2020, 12, 56361–56371. 10.1021/acsami.0c17658. 33270412

[ref27] Chun S.; Son W.; Kim D. W.; Lee J.; Min H.; Jung H.; Kwon D.; Kim A.-H.; Kim Y.-J.; Lim S. K.; Pang C.; Choi C. Water-Resistant and Skin-Adhesive Wearable Electronics Using Graphene Fabric Sensor with Octopus-Inspired Microsuckers. ACS Appl. Mater. Interfaces 2019, 11, 16951–16957. 10.1021/acsami.9b04206. 31034198

[ref28] Ko Y.; Oh J.; Park K. T.; Kim S.; Huh W.; Sung B. J.; Lim J. A.; Lee S.-S.; Kim H. Stretchable Conductive Adhesives with Superior Electrical Stability as Printable Interconnects in Washable Textile Electronics. ACS Appl. Mater. Interfaces 2019, 11, 37043–37050. 10.1021/acsami.9b11557. 31518103

[ref29] Lee J. H.; Nam Y. W.; Jung H.-C.; Baek D.-H.; Lee S.-H.; Hong J. S. Shear Induced CNT/PDMS Conducting Thin Film for Electrode Cardiogram (ECG) Electrode. BioChip J. 2012, 6, 91–98. 10.1007/s13206-012-6112-9.

[ref30] Yamamoto Y.; Yamamoto D.; Takada M.; Naito H.; Arie T.; Akita S.; Takei K. Efficient Skin Temperature Sensor and Stable Gel-Less Sticky ECG Sensor for a Wearable Flexible Healthcare Patch. Adv. Healthcare Mater. 2017, 6, 1700495 10.1002/adhm.201700495. 28661047

[ref31] Kim T.; Park J.; Sohn J.; Cho D.; Jeon S. Bioinspired, Highly Stretchable, and Conductive Dry Adhesives Based on 1D–2D Hybrid Carbon Nanocomposites for All-in-One ECG Electrodes. ACS Nano 2016, 10, 4770–4778. 10.1021/acsnano.6b01355. 26986477

[ref32] Jung H.-C.; Moon J.-H.; Baek D.-H.; Lee J.-H.; Choi Y.-Y.; Hong J.-S.; Lee S.-H. CNT/PDMS Composite Flexible Dry Electrodesfor Long-Term ECG Monitoring. IEEE Trans. Biomed. Eng. 2012, 59, 1472–1479. 10.1109/TBME.2012.2190288. 22410324

[ref33] Gilshteyn E. P.; Lin S.; Kondrashov V. A.; Kopylova D. S.; Tsapenko A. P.; Anisimov A. S.; Hart A. J.; Zhao X.; Nasibulin A. G. A One-Step Method of Hydrogel Modification by Single-Walled Carbon Nanotubes for Highly Stretchable and Transparent Electronics. ACS Appl. Mater. Interfaces 2018, 10, 28069–28075. 10.1021/acsami.8b08409. 30052424

[ref34] Hossain M. F.; Heo J. S.; Nelson J.; Kim I. Paper-Based Flexible Electrode Using Chemically-Modified Graphene and Functionalized Multiwalled Carbon Nanotube Composites for Electrophysiological Signal Sensing. Information 2019, 10, 325 10.3390/info10100325.

[ref35] Taylor L. W.; Williams S. M.; Yan J. S.; Dewey O. S.; Vitale F.; Pasquali M. Washable, Sewable, All-Carbon Electrodes and Signal Wires for Electronic Clothing. Nano Lett. 2021, 21, 7093–7099. 10.1021/acs.nanolett.1c01039. 34459618

[ref36] Jin H.; Abu-Raya Y. S.; Haick H. Advanced Materials for Health Monitoring with Skin-Based Wearable Devices. Adv. Healthcare Mater. 2017, 6, 1700024 10.1002/adhm.201700024. 28371294

[ref37] Xu P. J.; Zhang H.; Tao X. M. Textile-Structured Electrodes for Electrocardiogram. Text. Prog. 2008, 40, 183–213. 10.1080/00405160802597479.

[ref38] Connolly M.; Buckley D. A. Contact Dermatitis from Propylene Glycol in ECG Electrodes, Complicated by Medicament Allergy. Contact Dermatitis 2004, 50, 42–42. 10.1111/j.0105-1873.2004.00271c.x. 15059104

[ref39] Jóźwiak B.; Dzido G.; Zorȩbski E.; Kolanowska A.; Jȩdrysiak R.; Dziadosz J.; Libera M.; Boncel S.; Dzida M. Remarkable Thermal Conductivity Enhancement in Carbon-Based Ionanofluids: Effect of Nanoparticle Morphology. ACS Appl. Mater. Interfaces 2020, 12, 38113–38123. 10.1021/acsami.0c09752. 32649171 PMC7458364

[ref40] Kolanowska A.; Kuziel A. W.; Herman A. P.; Jędrysiak R. G.; Giżewski T.; Boncel S. Electroconductive Textile Coatings from Pastes Based on Individualized Multi-Wall Carbon Nanotubes – Synergy of Surfactant and Nanotube Aspect Ratio. Prog. Org. Coat. 2019, 130, 260–269. 10.1016/j.porgcoat.2019.01.042.

[ref41] Dziubiński M.; Kiljański T.; Sęk J. Theoretical basis and measuring methods of rheology; Lodz University of Technology Press: Łódź, 2014.

[ref42] Boncel S.; Pluta A.; Skonieczna M.; Gondela A.; Maciejewska B.; Herman A. P.; Jędrysiak R. G.; Budniok S.; Komędera K.; Błachowski A.; Walczak K. Z. Hybrids of Iron-Filled Multiwall Carbon Nanotubes and Anticancer Agents as Potential Magnetic Drug Delivery Systems: In Vitro Studies against Human Melanoma, Colon Carcinoma, and Colon Adenocarcinoma. J. Nanomater. 2017, 2017, 1262309 10.1155/2017/1262309.

[ref43] Aly K.; Li A.; Bradford P. D. Compressive Piezoresistive Behavior of Carbon Nanotube Sheets Embedded in Woven Glass Fiber Reinforced Composites. Composites, Part B 2017, 116, 459–470. 10.1016/j.compositesb.2016.11.002.

[ref44] Faraji S.; Stano K.; Rost C.; Maria J.-P.; Zhu Y.; Bradford P. D. Structural Annealing of Carbon Coated Aligned Multi-Walled Carbon Nanotube Sheets. Carbon 2014, 79, 113–122. 10.1016/j.carbon.2014.07.049.

[ref45] Yildiz O.; Stano K.; Faraji S.; Stone C.; Willis C.; Zhang X.; Jur J. S.; Bradford P. D. High Performance Carbon Nanotube – Polymer Nanofiber Hybrid Fabrics. Nanoscale 2015, 7, 16744–16754. 10.1039/C5NR02732B. 26399497

[ref46] Yildiz O.; Bradford P. D. Aligned Carbon Nanotube Sheet High Efficiency Particulate Air Filters. Carbon 2013, 64, 295–304. 10.1016/j.carbon.2013.07.066.

[ref47] Chng E. L. K.; Poh H. L.; Sofer Z.; Pumera M. Purification of Carbon Nanotubes by High Temperature Chlorine Gas Treatment. Phys. Chem. Chem. Phys. 2013, 15, 5615 10.1039/c3cp50348h. 23471202

[ref48] Dresselhaus M. S.; Dresselhaus G.; Saito R.; Jorio A. Raman Spectroscopy of Carbon Nanotubes. Phys. Rep. 2005, 409, 47–99. 10.1016/j.physrep.2004.10.006.

[ref49] Jorio A.; Saito R. Raman Spectroscopy for Carbon Nanotube Applications. J. Appl. Phys. 2021, 129, 021102 10.1063/5.0030809.

[ref50] Li H.; Papadakis R.; Jafri S.; Hassan M.; Thersleff T.; Michler J.; Ottosson H.; Leifer K. Superior Adhesion of Graphene Nanoscrolls. Commun. Phys. 2018, 1, 44 10.1038/s42005-018-0043-2.

[ref51] Crawford N.; Meyer F. Investigating the Shear Flow and Thixotropic Behavior of Paints and Coatings; Thermo Fisher Scientific: Karlsruhe, 2019.

[ref52] Wang C.-S.; Chapelle G.; Carreau P.; Heuzey M.-C. Prediction of Sag Resistance in Paints Using Rheological Measurements. Prog. Org. Coat. 2021, 153, 106139 10.1016/j.porgcoat.2021.106139.

[ref53] Jóźwiak B.; Dzido G.; Kolanowska A.; Jędrysiak R. G.; Zorębski E.; Greer H. F.; Dzida M.; Boncel S. From Lab and up: Superior and Economic Heat Transfer Performance of Ionanofluids Containing Long Carbon Nanotubes and 1-Ethyl-3-Methylimidazolium Thiocyanate. Int. J. Heat Mass Transfer 2021, 172, 121161 10.1016/j.ijheatmasstransfer.2021.121161.

[ref54] Deshpande A. P.; Krishnan J. M.; Kumar S. Rheology of Complex Fluids; Springer: Dordrecht, 2010.

[ref55] Larson R. G.; Wei Y. A Review of Thixotropy and Its Rheological Modeling. J. Rheol. 2019, 63, 477–501. 10.1122/1.5055031.

[ref56] Ramasamy S.; Balan A. Wearable Sensors for ECG Measurement: A Review. Sens. Rev. 2018, 38, 412–419. 10.1108/SR-06-2017-0110.

[ref57] Huczko A.; Lange H. Carbon Nanotubes: Experimental Evidence For A Null Risk Of Skin Irritation And Allergy. Fullerene Sci. Technol. 2001, 9, 247–250. 10.1081/FST-100102972.

[ref58] Kishore A. S.; Surekha P.; Murthy P. B. Assessment of the Dermal and Ocular Irritation Potential of Multi-Walled Carbon Nanotubes by Using in Vitro and in Vivo Methods. Toxicol. Lett. 2009, 191, 268–274. 10.1016/j.toxlet.2009.09.007. 19770026

[ref59] Kumar P. S.; Rai P.; Oh S.; Kwon H.; Varadan V. K. Nanocomposite Electrodes for Smartphone Enabled Healthcare Garments: E-Bra and Smart Vest; Choi S. H.; Choy J.-H.; Lee U.; Varadan V. K., Eds.; Incheon, Republic of Korea, 2012; p 85481O.

[ref60] Boncel S.; Müller K. H.; Skepper J. N.; Walczak K. Z.; Koziol K. K. K. Tunable Chemistry and Morphology of Multi-Wall Carbon Nanotubes as a Route to Non-Toxic, Theranostic Systems. Biomaterials 2011, 32, 7677–7686. 10.1016/j.biomaterials.2011.06.055. 21764122

