# Supplementary material for: Paintable Carbon Nanotube Coating-Based Textronics for Sustained Holter-Type Electrocardiography
Source: ACS Appl Nano Mater. 2022 Oct 7;5(10):15762–74. doi: 10.1021/acsanm.2c03904 (PMC9623549; doi:10.1021/acsanm.2c03904)
Supplement: Supplementary file 1 — an2c03904_si_001.pdf [file an2c03904_si_001.pdf]

Supplementary Information

for

Paintable Carbon Nanotube Coating–based Textronics for Sustained Holter-type Electrocardiography

Sławomir Boncel[1,2,*], Rafał G. Jędrysiak[1,2], Marek Czerw[3,4], Anna Kolanowska[1,2,5,6], Anna W. Blacha[1,2], Maciej Imielski[1,2], Bertrand Jóźwiak[1,2,7], Marzena H. Dzida[8], Heather F. Greer[9], Aleksander Sobotnicki[3]

[1] Silesian University of Technology, Faculty of Chemistry, Department of Organic Chemistry, Bioorganic Chemistry and Biotechnology, NanoCarbonGroup, Krzywoustego 4, 44-100 Gliwice, Poland

[2] Silesian University of Technology, Centre for Organic and Nanohybrid Electronics, Konarskiego 22B, 44-100 Gliwice, Poland

[3] Łukasiewicz Research Network Institute of Medical Technology and Equipment, Roosevelta 118, 41-800 Zabrze, Poland

[4] Silesian University of Technology, Department of Biosensors and Processing of Biomedical Signals, Roosevelta 40, 41-800 Zabrze, Poland

[5] Silesian University of Technology, Department of Physical Chemistry and Technology of Polymers, Marcina Strzody 9, 44-100 Gliwice, Poland

[6] Silesian University of Technology, Biotechnology Centre, Krzywoustego 8, 44-100 Gliwice, Poland

[7] Silesian University of Technology, Department of Chemical Engineering and Process Design, Marcina Strzody 7, 44-100 Gliwice, Poland

[8] University of Silesia in Katowice, Institute of Chemistry, Szkolna 9, 40-006 Katowice, Poland

[9] University of Cambridge, Department of Chemistry, Cambridge CB2 1EW, UK

Corresponding author: Sławomir Boncel, e-mail: slawomir.boncel@polsl.pl

Supplementary Information contains SEM images of L-MWCNTs, optical micrographs of MWCNTs embedded in the paint, detailed Holter-type data, i.e. ECG and HR recordings, and documentation on the T-shirt textronics as 50 figures.

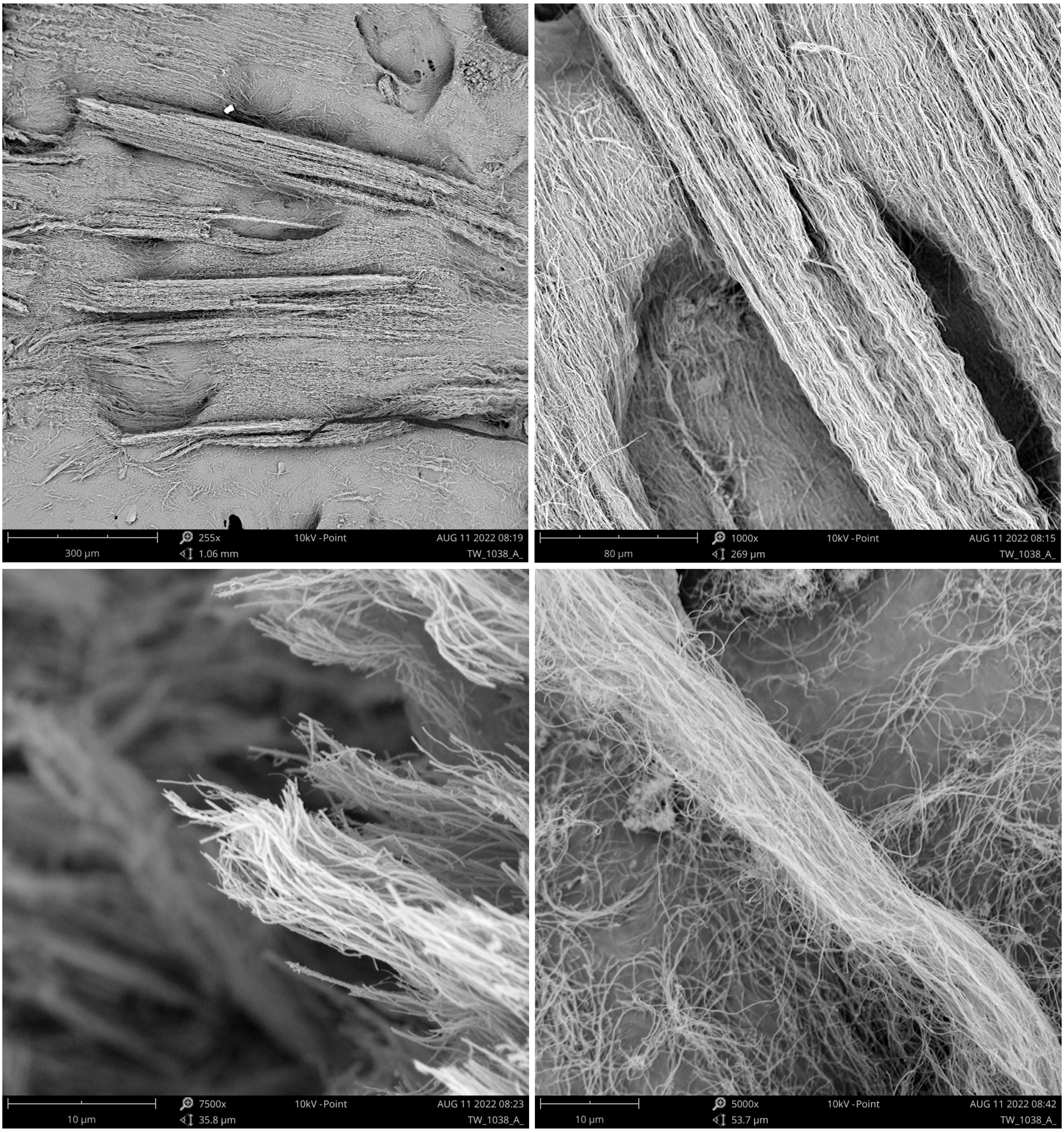

Fig. S1 SEM images of as-synthesized 800-μm-long MWCNTs

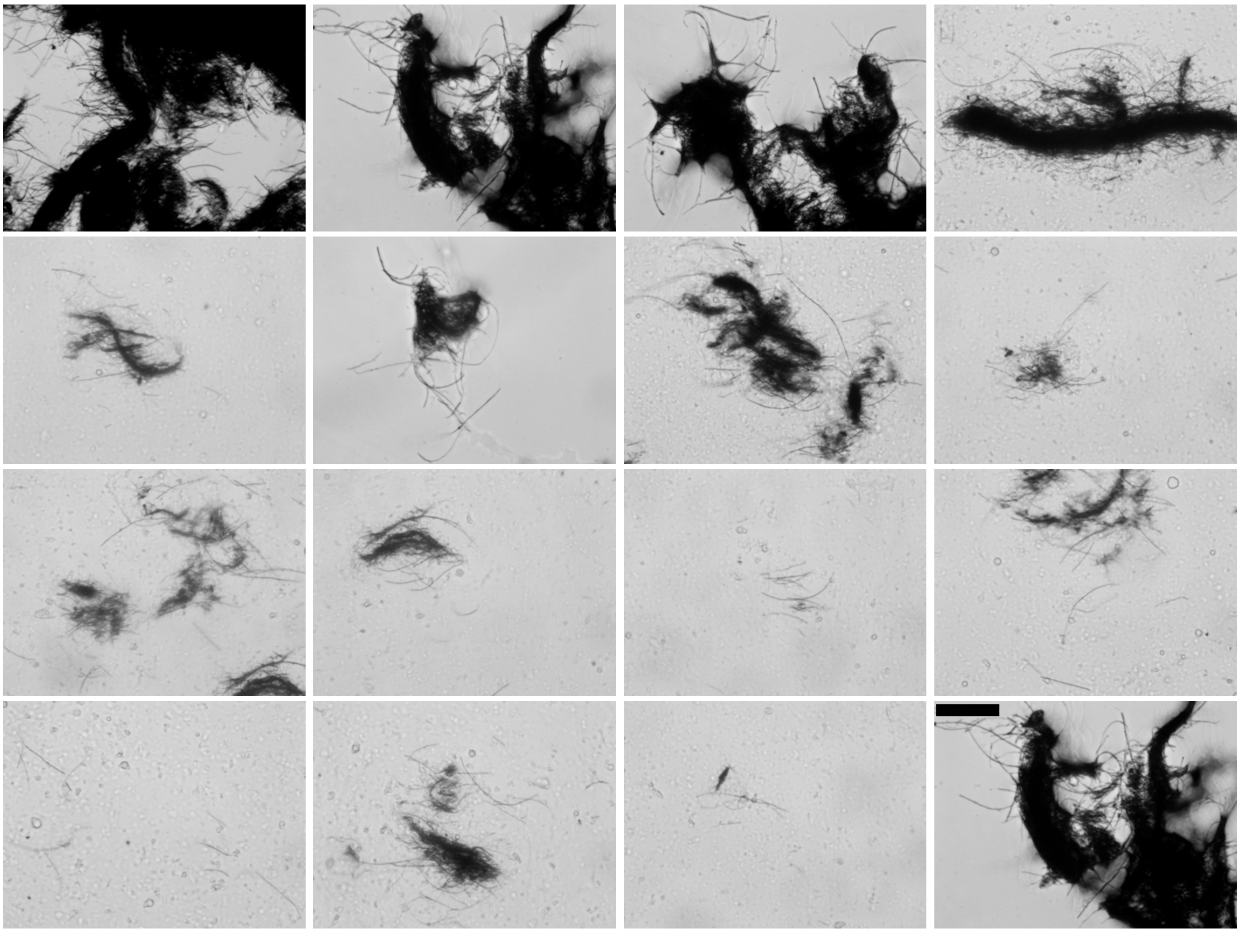

Fig. S2 Optical micrographs used to determine the MWCNT bundle length distribution in the paint; scale bar = 50 μm

Fig. S3-S19 Detailed sections of the ECG and HR recording intervals of daily activity of the male individual as print-screens from the commercial EMTEL ArchiwumFX® software

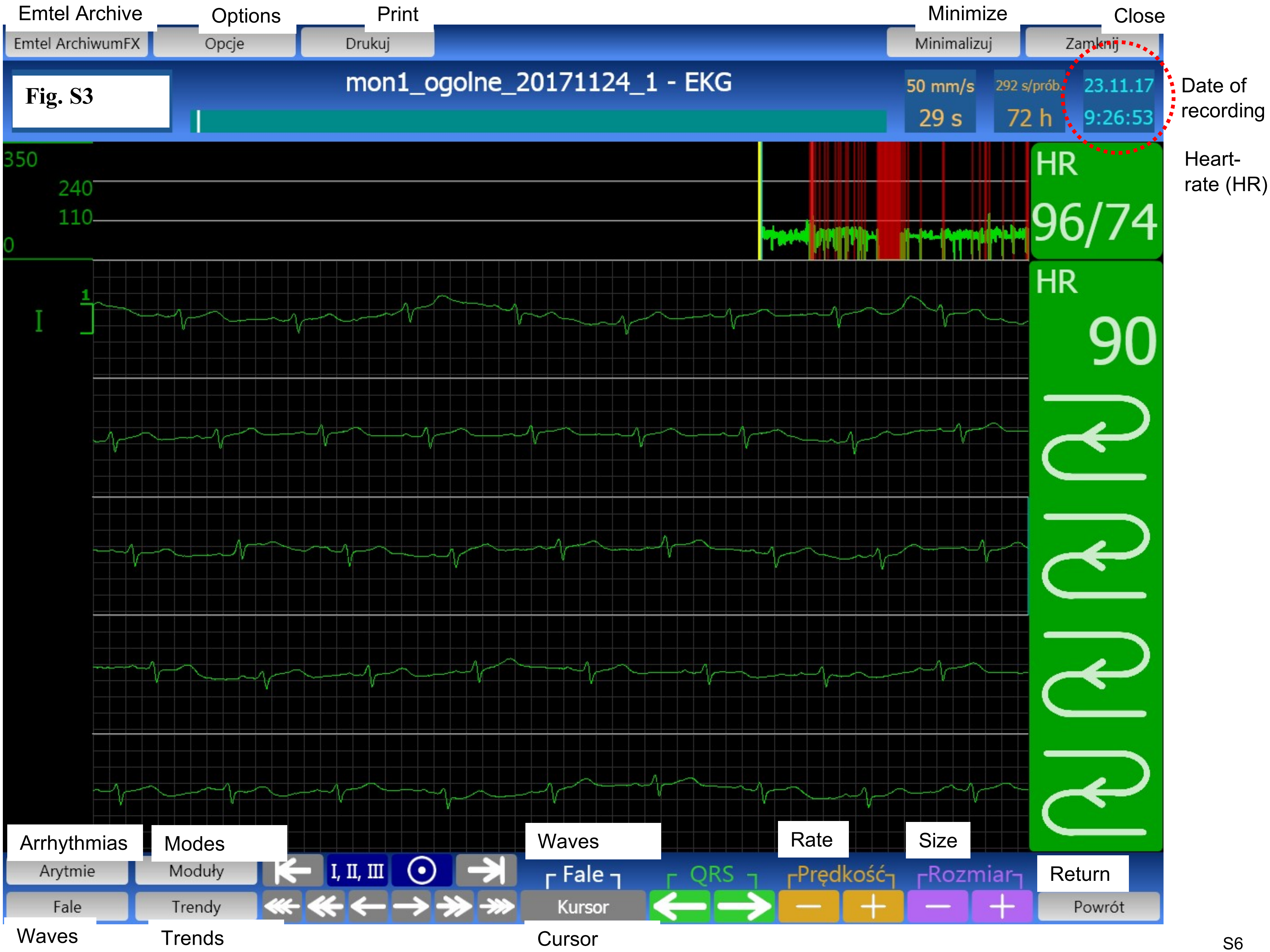

Emtel Archive
Options
Print
Minimize
Close
Emtel ArchiwumFX
Opcje
Drukuj
Minimalizuj
Zamknij
mon1_ogolne_20171124_1 - EKG
50 mm/s
29 s
292 s/prób.
72 h
23.11.17
9:26:53
Date of recording
350
240
110
0
HR
96/74
Heart-rate (HR)
I
1
HR
90
Arrhythmias
Modes
Waves
Rate
Size
Arytmie
Moduły
I, II, III
Fale
QRS
Prędkość
Rozmiar
Return
Fale
Trendy
Kursor
Powrót
Waves
Trends
Cursor

Fig. S3

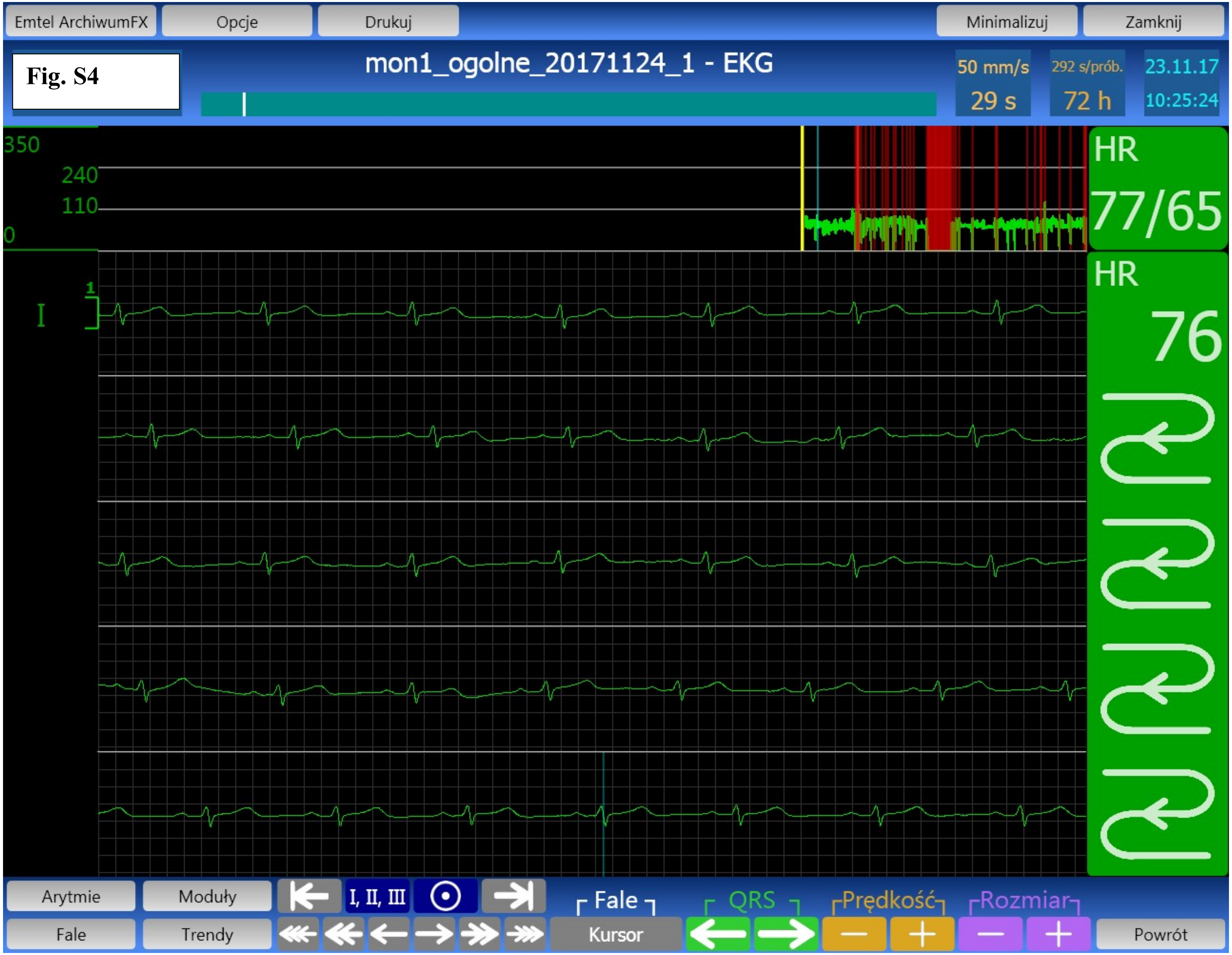
Emtel ArchiwumFX
Opcje
Drukuj
Minimalizuj
Zamknij
Fig. S4
mon1_ogolne_20171124_1 - EKG
50 mm/s
29 s
292 s/prób.
72 h
23.11.17
10:25:24
350
240
110
0
HR
77/65
HR
76
I
Arytmie
Moduły
I, II, III
Fale
Trendy
Fale
Kursor
QRS
Prędkość
Rozmiar
Powrót

Fig. S5

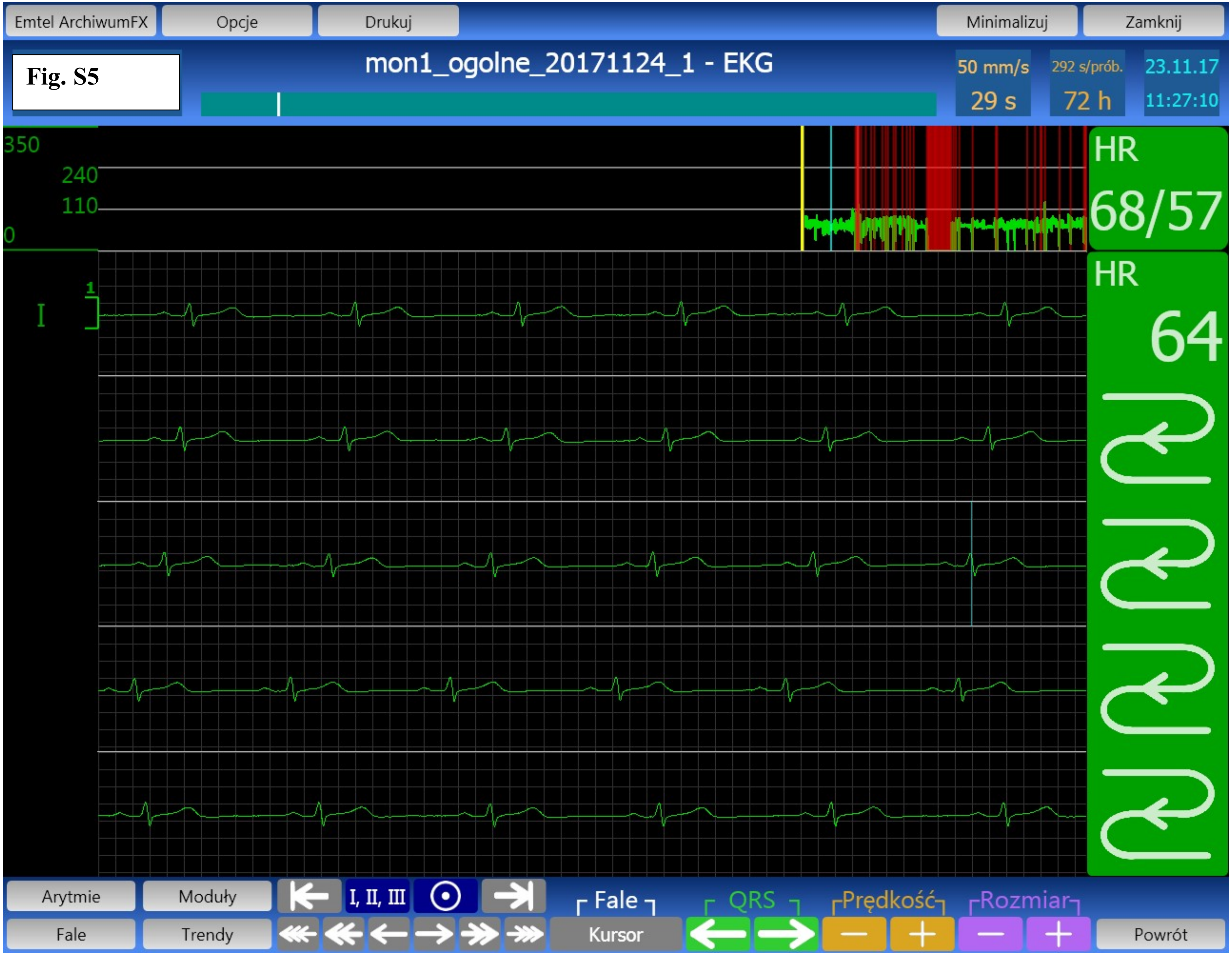
Emtel ArchiwumFX
Opcje
Drukuj
Minimalizuj
Zamknij
mon1_ogolne_20171124_1 - EKG
50 mm/s
29 s
292 s/prób.
72 h
23.11.17
11:27:10
350
240
110
0
HR
68/57
HR
64
I
Arytmie
Moduły
Fale
Trendy
I, II, III
Fale
Kursor
QRS
Prędkość
Rozmiar
Powrót

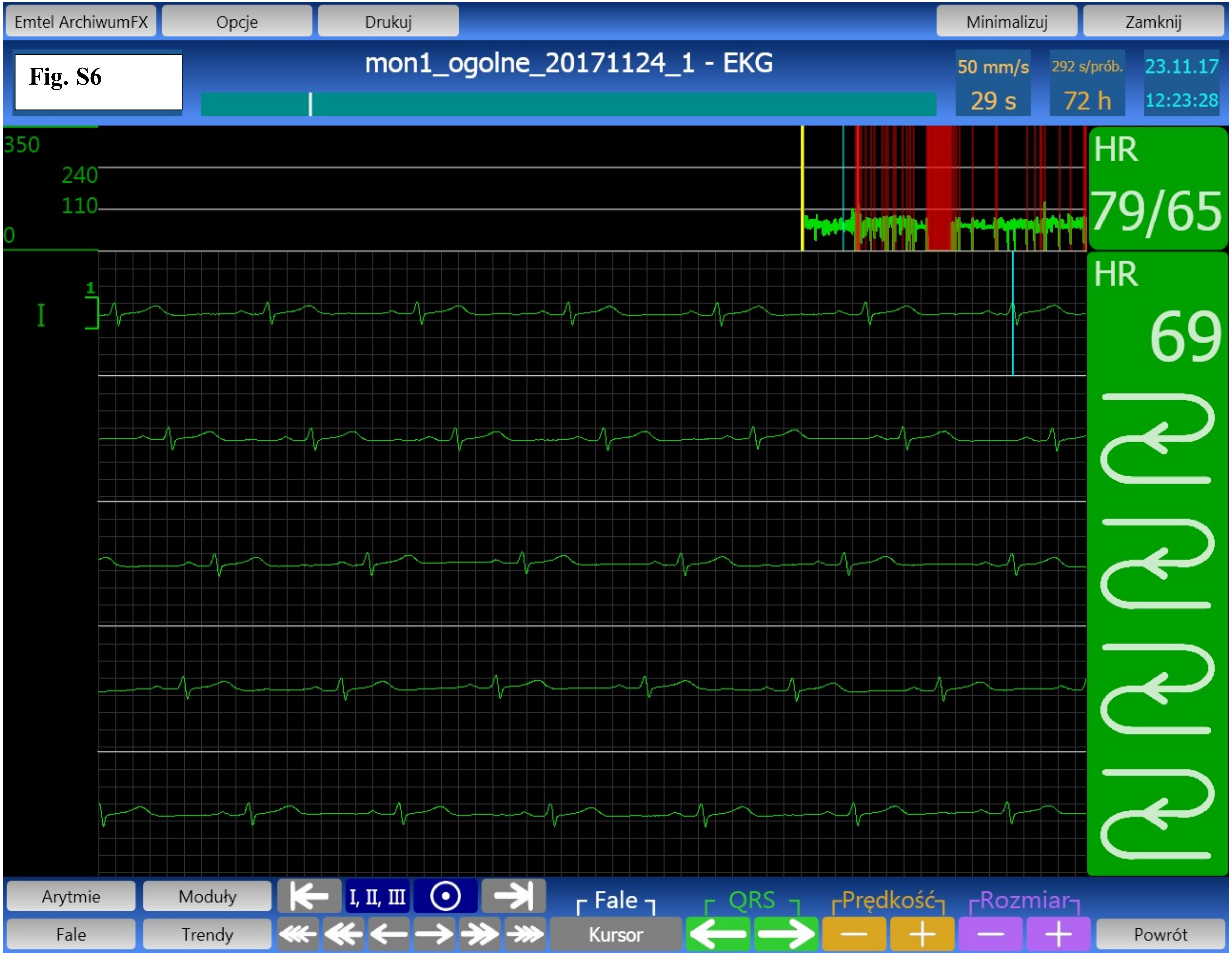
Emtel ArchiwumFX
Opcje
Drukuj
Minimalizuj
Zamknij
Fig. S6
mon1_ogolne_20171124_1 - EKG
50 mm/s
29 s
292 s/prób.
72 h
23.11.17
12:23:28
350
240
110
0
HR
79/65
I
1
HR
69
Arytmie
Moduły
I, II, III
Fale
Trendy
Fale
Kursor
QRS
Prędkość
Rozmiar
Powrót

Fig. S7

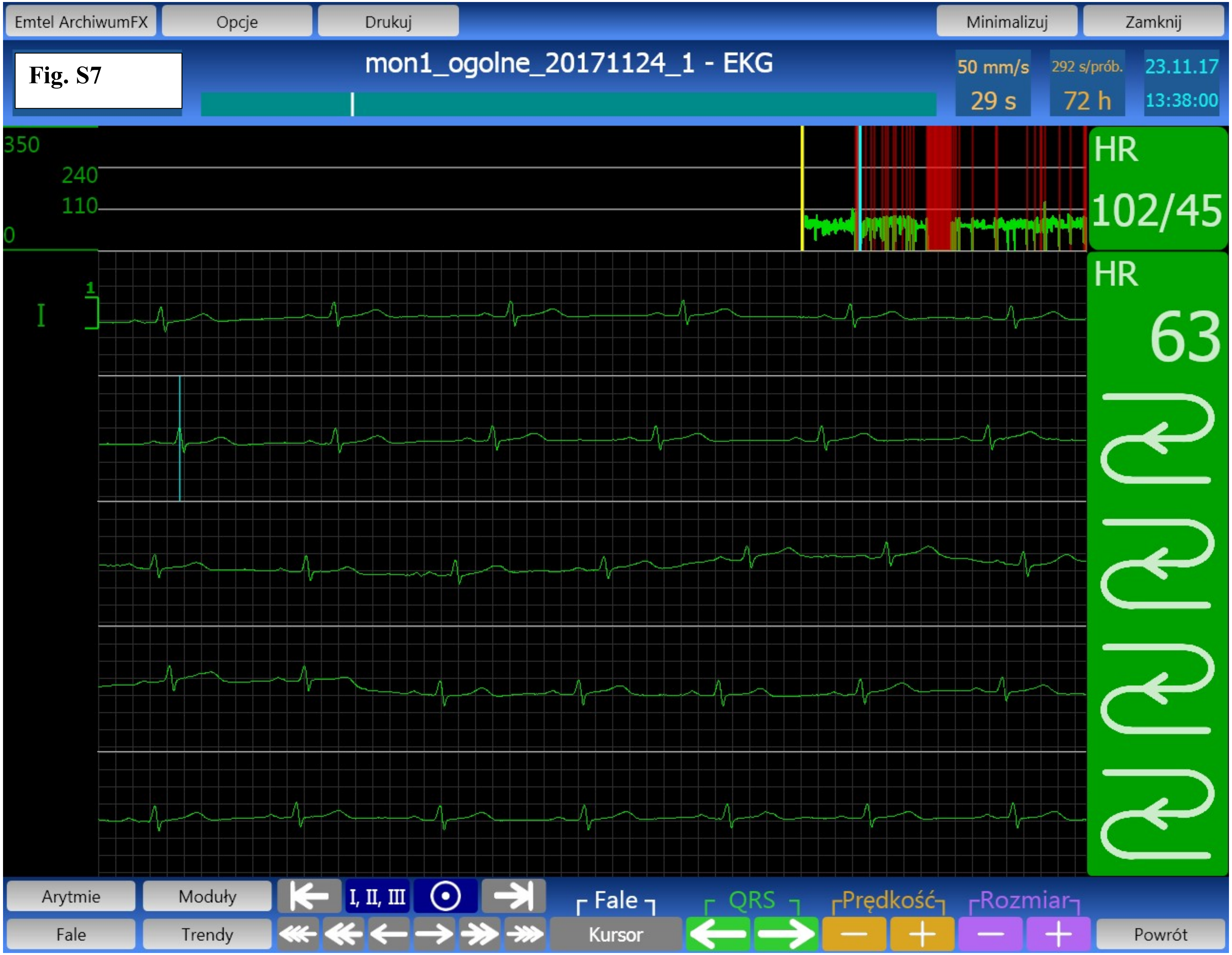

Emtel ArchiwumFX
Opcje
Drukuj
Minimalizuj
Zamknij
mon1_ogolne_20171124_1 - EKG
50 mm/s
29 s
292 s/prób.
72 h
23.11.17
13:38:00
350
240
110
0
HR
102/45
I
1
HR
63
Arytmie
Moduły
I, II, III
Fale
Trendy
Fale
Kursor
QRS
Prędkość
Rozmiar
Powrót

Fig. S8

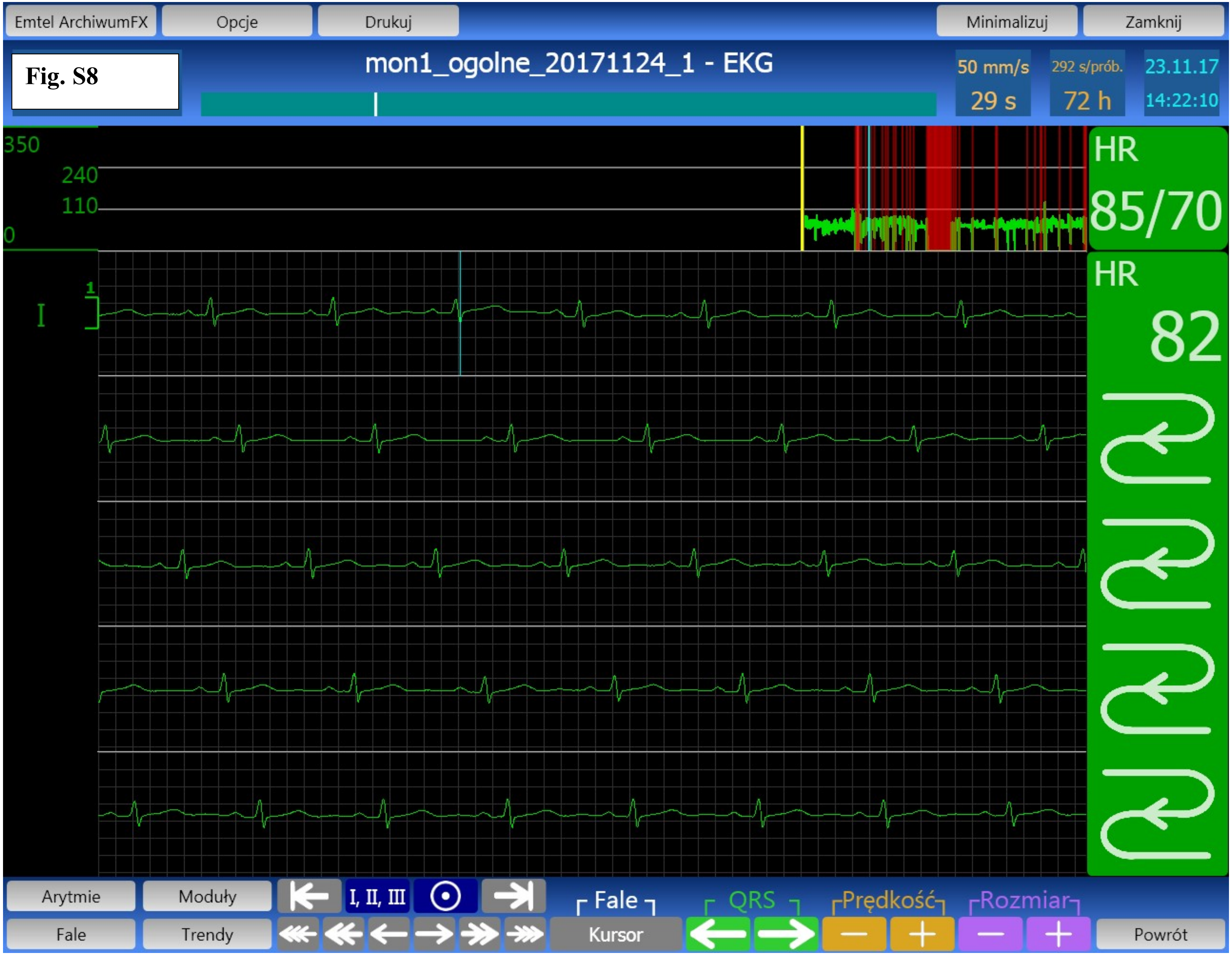

Emtel ArchiwumFX
Opcje
Drukuj
Minimalizuj
Zamknij
mon1_ogolne_20171124_1 - EKG
50 mm/s
29 s
292 s/prób.
72 h
23.11.17
14:22:10
350
240
110
0
HR
85/70
I
1
HR
82
Arytmie
Moduły
I, II, III
Fale
Kursor
QRS
Prędkość
Rozmiar
Fale
Trendy
Powrót

Fig. S9

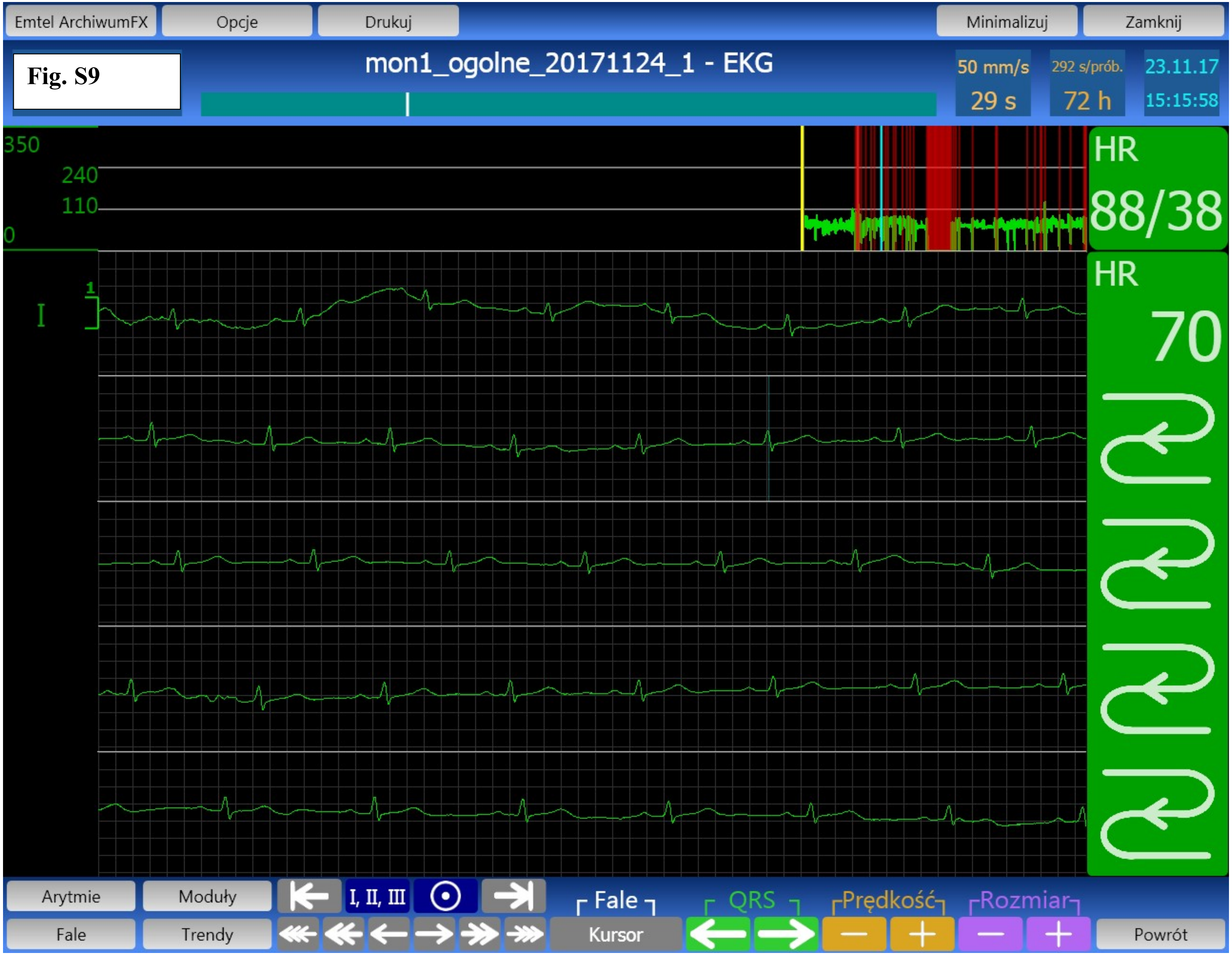

Emtel ArchiwumFX
Opcje
Drukuj
Minimalizuj
Zamknij
mon1_ogolne_20171124_1 - EKG
50 mm/s
29 s
292 s/prób.
72 h
23.11.17
15:15:58
350
240
110
0
HR
88/38
I
1
HR
70
Arytmie
Moduły
I, II, III
Fale
Trendy
Fale
Kursor
QRS
Prędkość
Rozmiar
Powrót

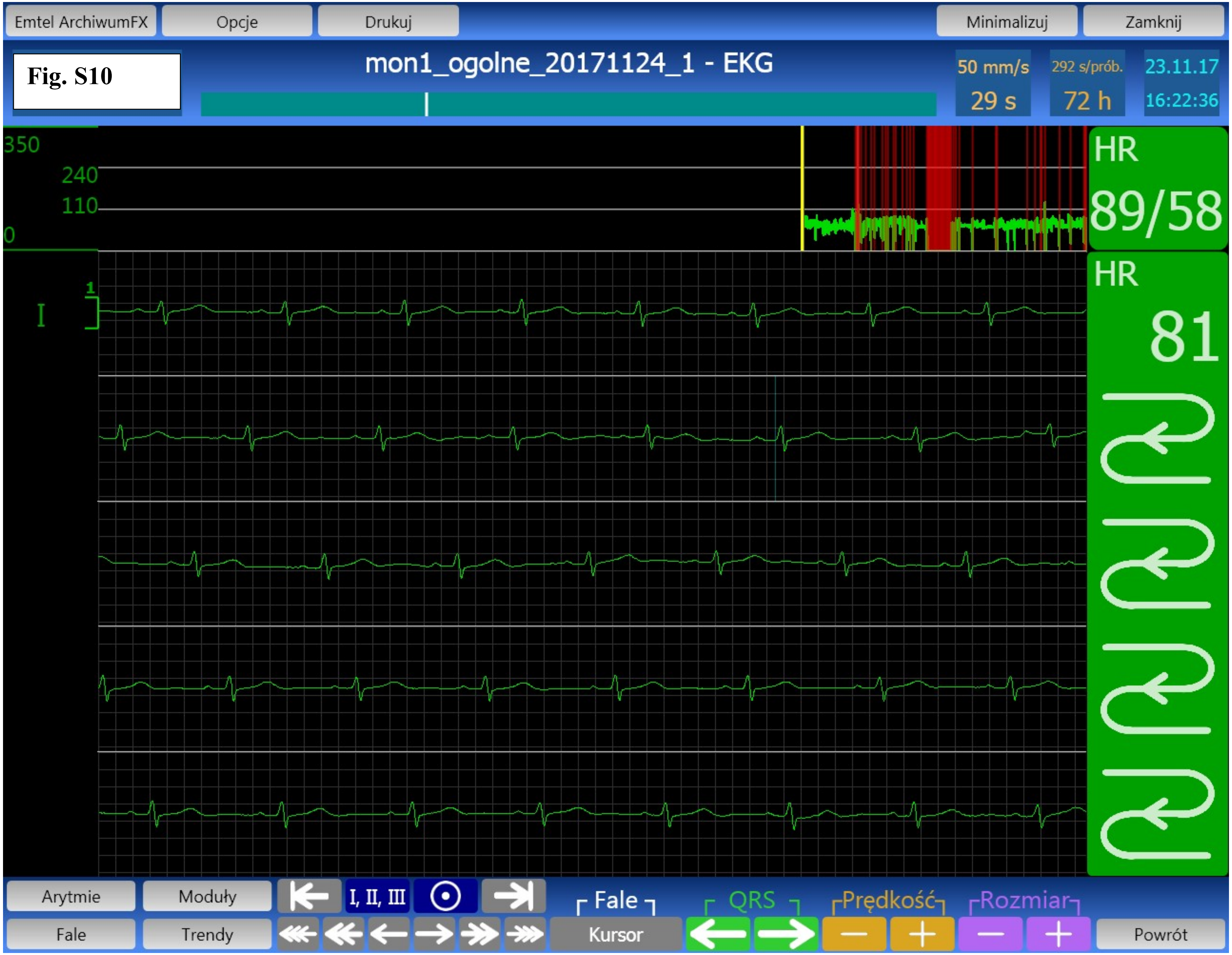
Emtel ArchiwumFX
Opcje
Drukuj
Minimalizuj
Zamknij
Fig. S10
mon1_ogolne_20171124_1 - EKG
50 mm/s
29 s
292 s/prób.
72 h
23.11.17
16:22:36
350
240
110
0
HR
89/58
I
1
HR
81
Arytmie
Moduły
I, II, III
Fale
Kursor
QRS
Prędkość
Rozmiar
Fale
Trendy
Powrót

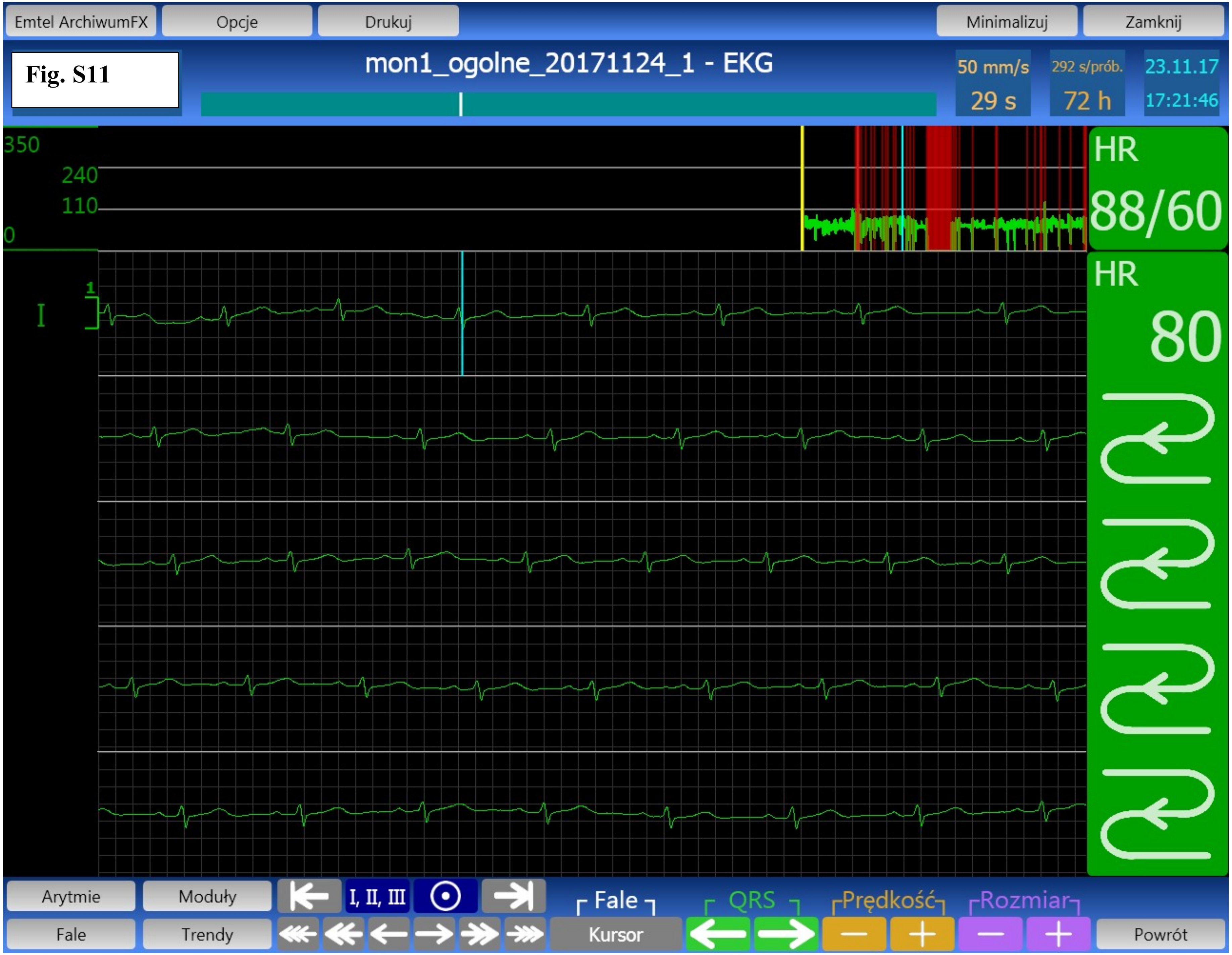
Emtel ArchiwumFX
Opcje
Drukuj
Minimalizuj
Zamknij
Fig. S11
mon1_ogolne_20171124_1 - EKG
50 mm/s
29 s
292 s/prób.
72 h
23.11.17
17:21:46
350
240
110
0
HR
88/60
HR
80
I
1
Arytmie
Moduły
I, II, III
Fale
Kursor
QRS
Prędkość
Rozmiar
Trendy
Powrót

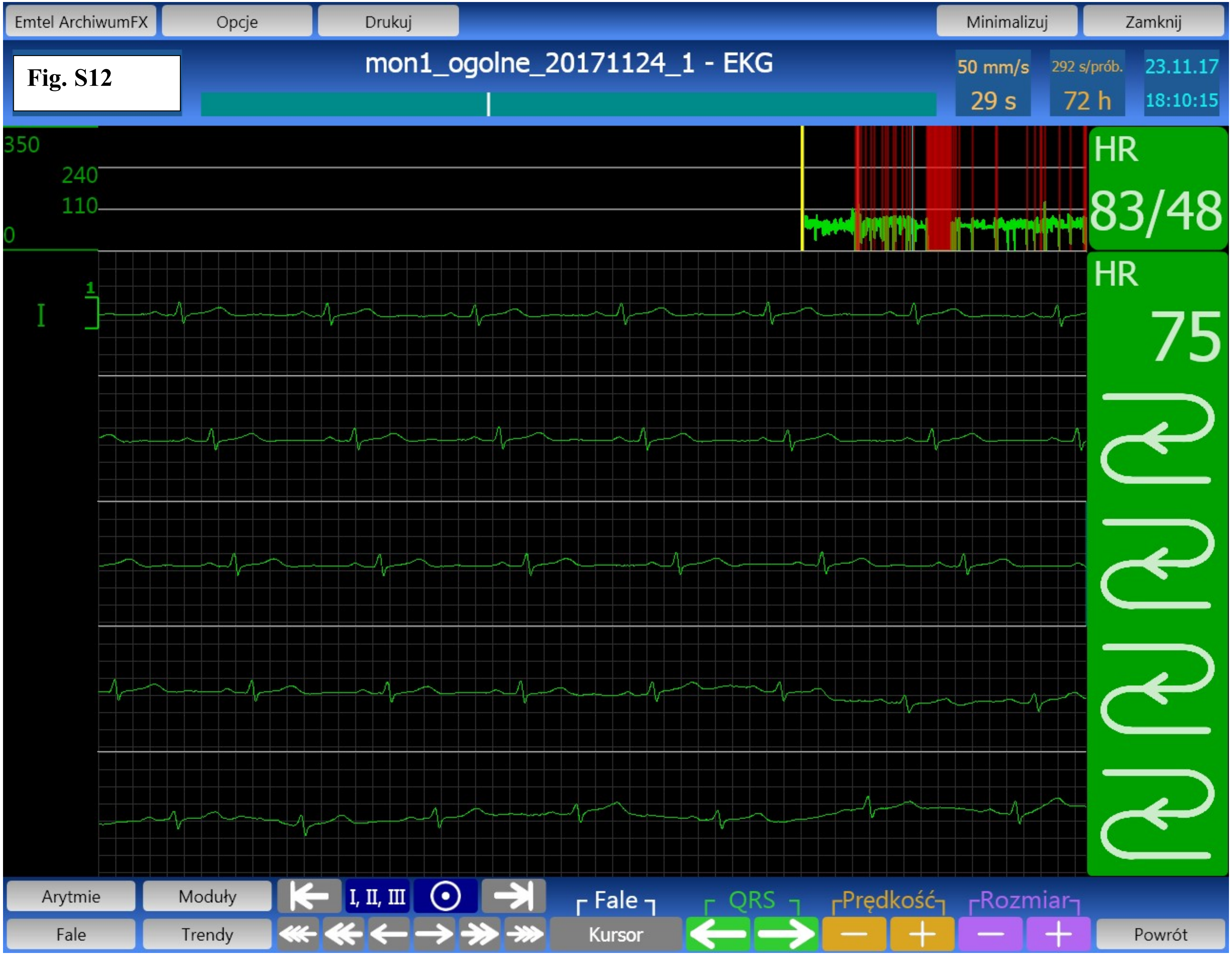
Fig. S12
Emtel ArchiwumFX
Opcje
Drukuj
Minimalizuj
Zamknij
mon1_ogolne_20171124_1 - EKG
50 mm/s
29 s
292 s/prób.
72 h
23.11.17
18:10:15
350
240
110
0
HR
83/48
HR
75
I
1
Arytmie
Moduły
I, II, III
Fale
Kursor
QRS
Prędkość
Rozmiar
Trendy
Powrót

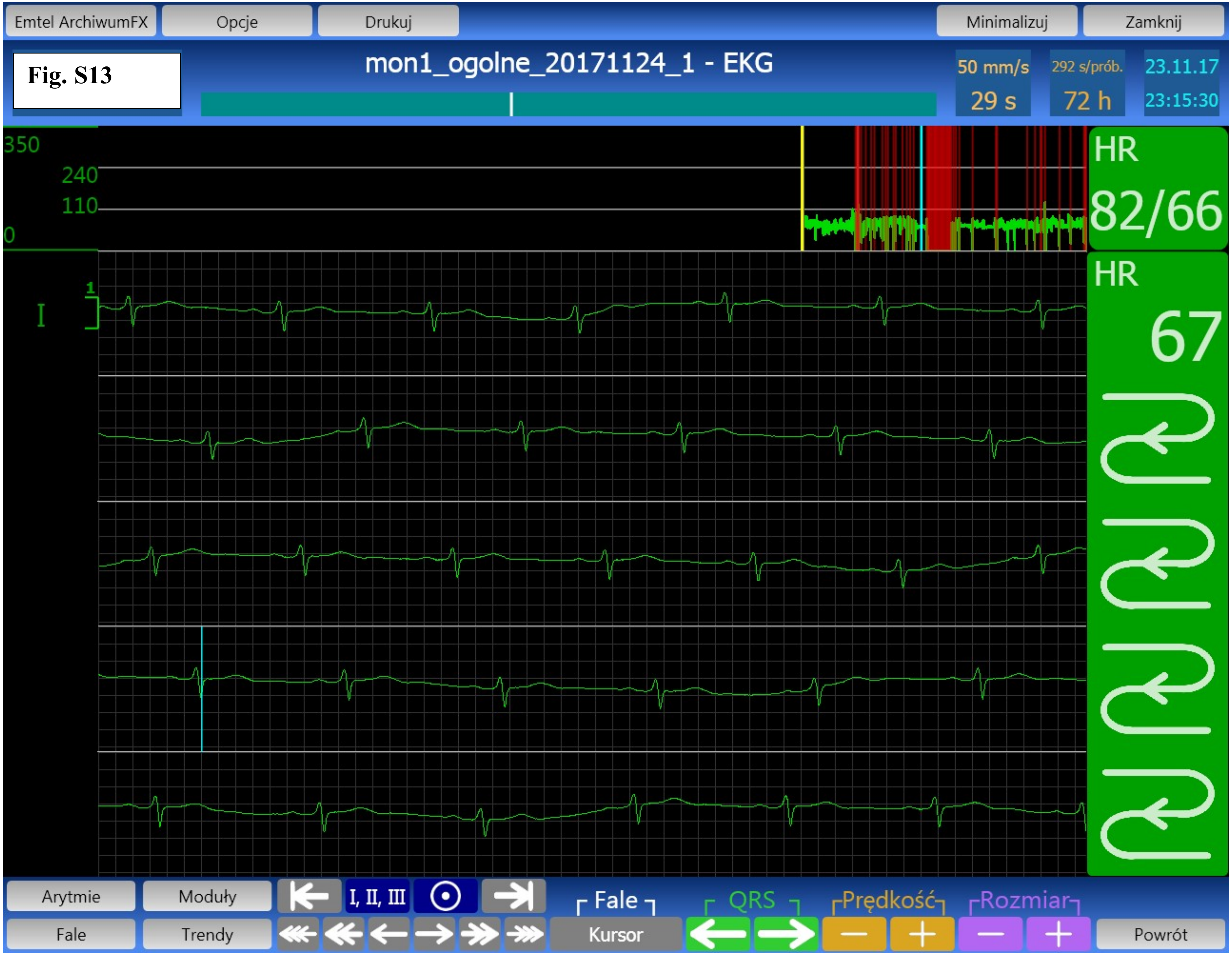

Emtel ArchiwumFX
Opcje
Drukuj
Minimalizuj
Zamknij
Fig. S13
mon1_ogolne_20171124_1 - EKG
50 mm/s
29 s
292 s/prób.
72 h
23.11.17
23:15:30
350
240
110
0
HR
82/66
HR
67
I
1
Arytmie
Moduły
I, II, III
Fale
Trendy
Fale
Kursor
QRS
Prędkość
Rozmiar
Powrót

Fig. S14

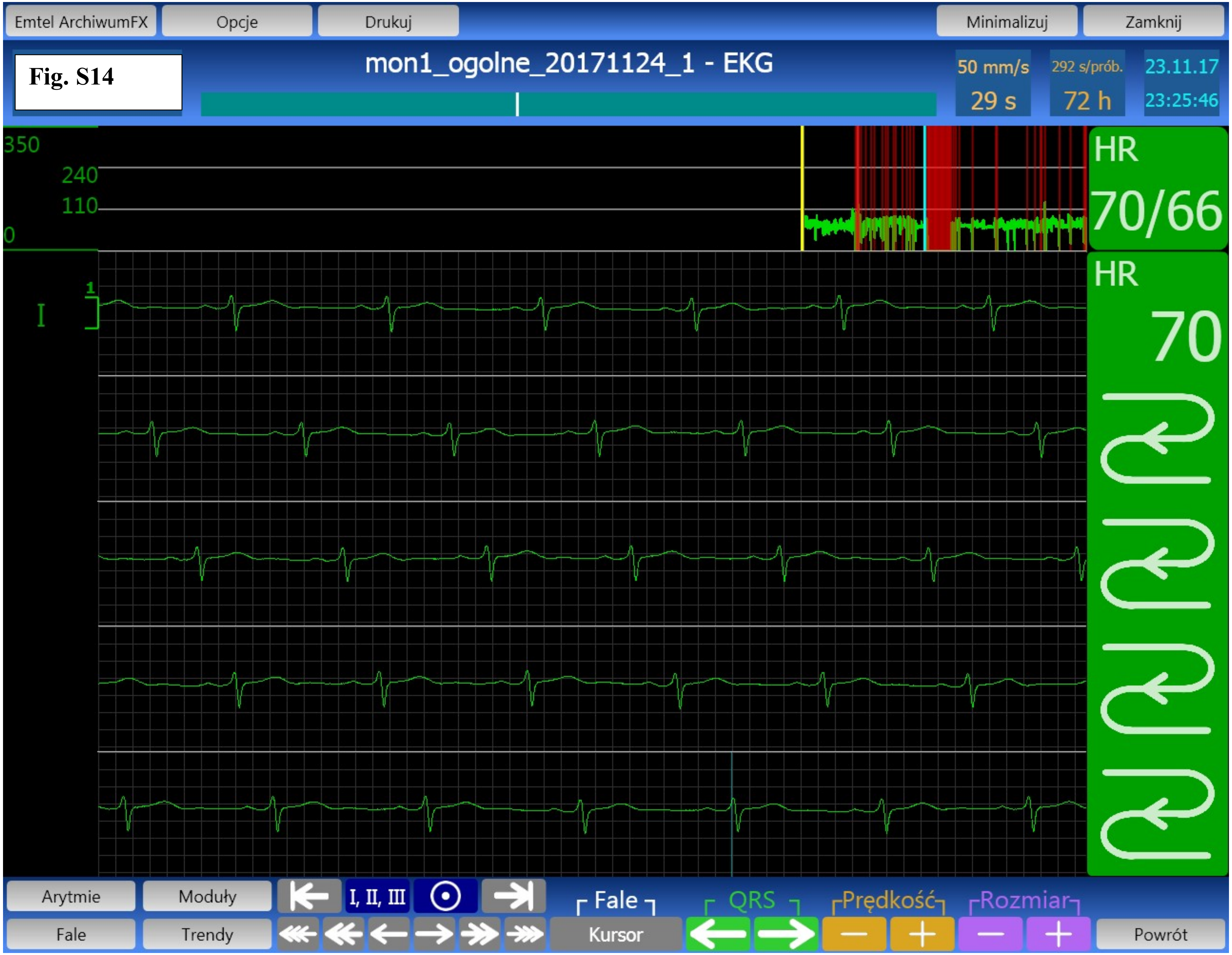

Emtel ArchiwumFX
Opcje
Drukuj
Minimalizuj
Zamknij
mon1_ogolne_20171124_1 - EKG
50 mm/s
29 s
292 s/prób.
72 h
23.11.17
23:25:46
350
240
110
0
HR
70/66
I
HR
70
Arytmie
Moduły
I, II, III
Fale
Kursor
QRS
Prędkość
Rozmiar
Fale
Trendy
Powrót

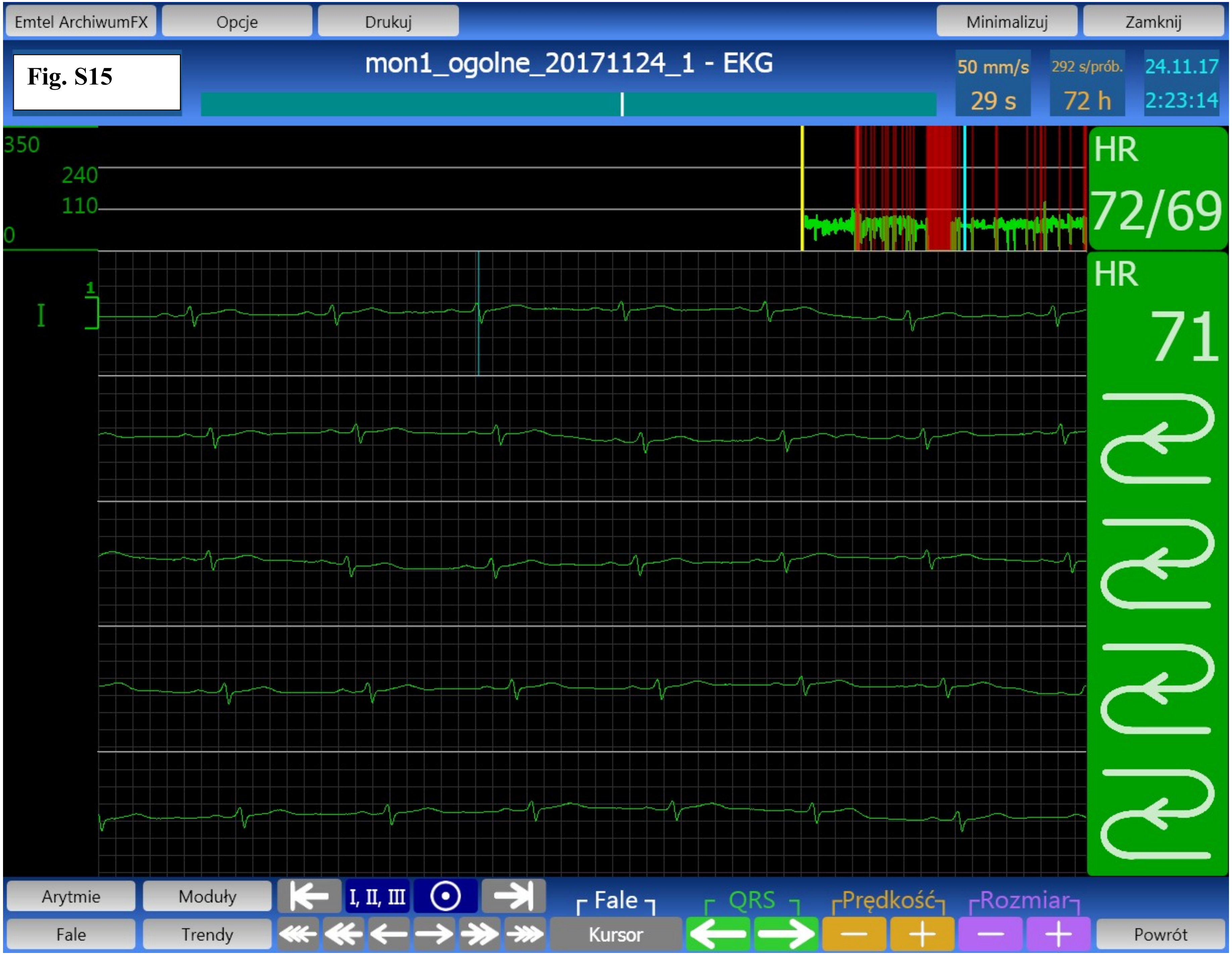
Emtel ArchiwumFX
Opcje
Drukuj
Minimalizuj
Zamknij
Fig. S15
mon1_ogolne_20171124_1 - EKG
50 mm/s
29 s
292 s/prób.
72 h
24.11.17
2:23:14
350
240
110
0
HR
72/69
HR
71
I
1
Arytmie
Moduły
I, II, III
Fale
Kursor
QRS
Prędkość
Rozmiar
Fale
Trendy
Powrót

Fig. S16

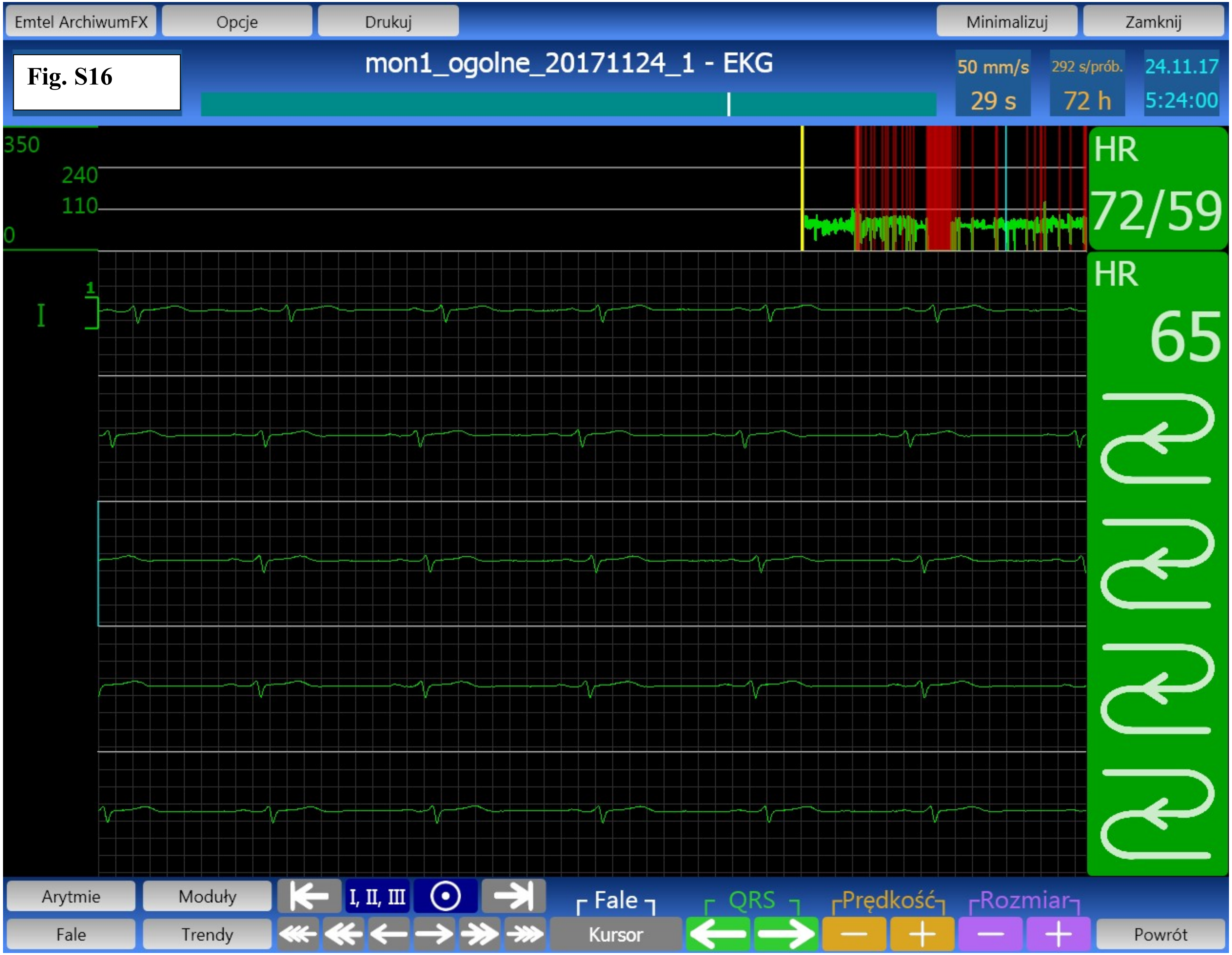
Emtel ArchiwumFX
Opcje
Drukuj
Minimalizuj
Zamknij
mon1_ogolne_20171124_1 - EKG
50 mm/s
29 s
292 s/prób.
72 h
24.11.17
5:24:00
350
240
110
0
HR
72/59
HR
65
I
1
Arytmie
Moduły
I, II, III
Fale
Kursor
QRS
Prędkość
Rozmiar
Trendy
Powrót

Fig. S17

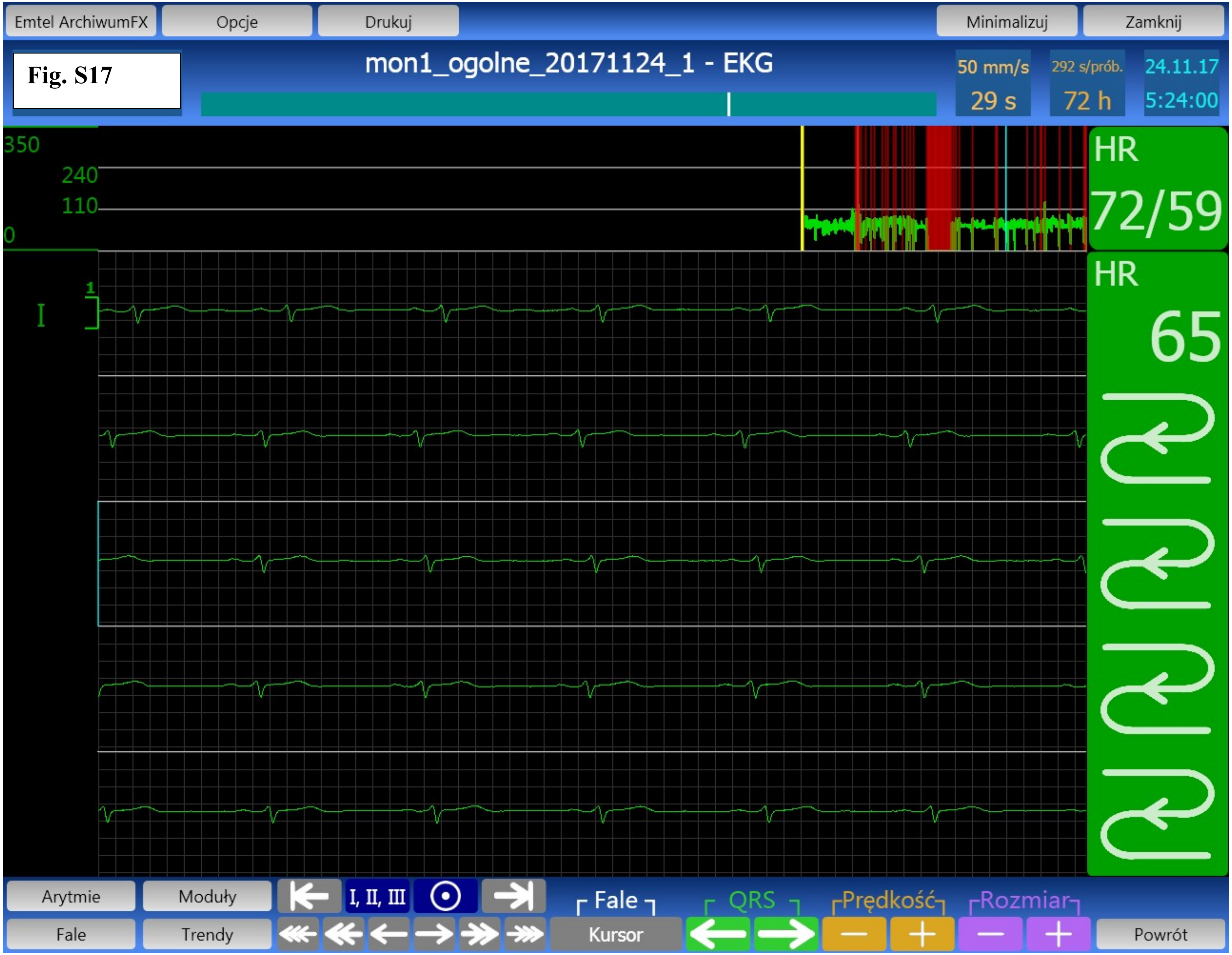
Emtel ArchiwumFX
Opcje
Drukuj
Minimalizuj
Zamknij
mon1_ogolne_20171124_1 - EKG
50 mm/s
29 s
292 s/prób.
72 h
24.11.17
5:24:00
350
240
110
0
HR
72/59
HR
65
I
1
Arytmie
Moduły
I, II, III
Fale
Trendy
Fale
Kursor
QRS
Prędkość
Rozmiar
Powrót

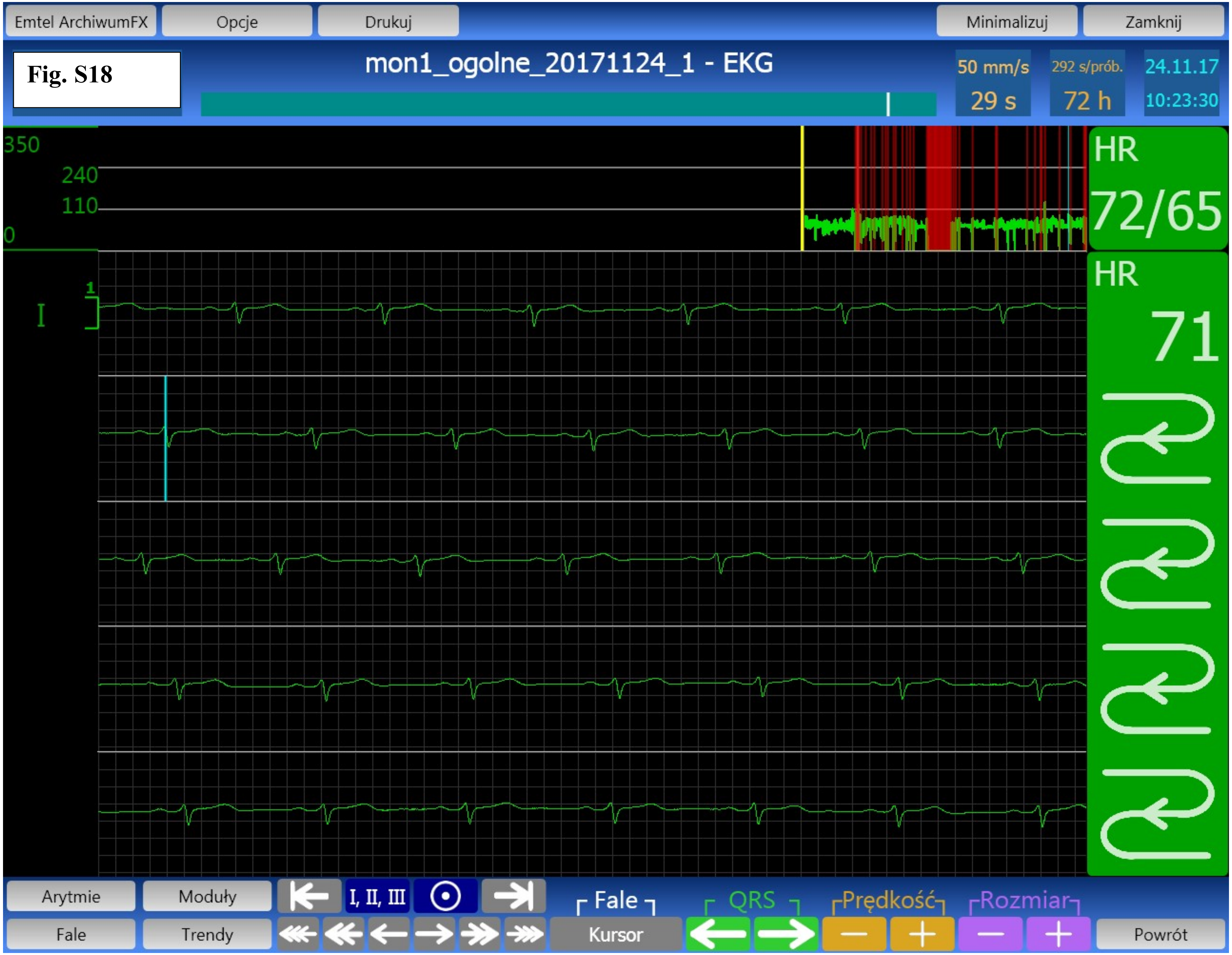
Emtel ArchiwumFX
Opcje
Drukuj
Minimalizuj
Zamknij
Fig. S18
mon1_ogolne_20171124_1 - EKG
50 mm/s
29 s
292 s/prób.
72 h
24.11.17
10:23:30
350
240
110
0
HR
72/65
HR
71
I
Arytmie
Moduły
I, II, III
Fale
Trendy
Fale
Kursor
QRS
Prędkość
Rozmiar
Powrót

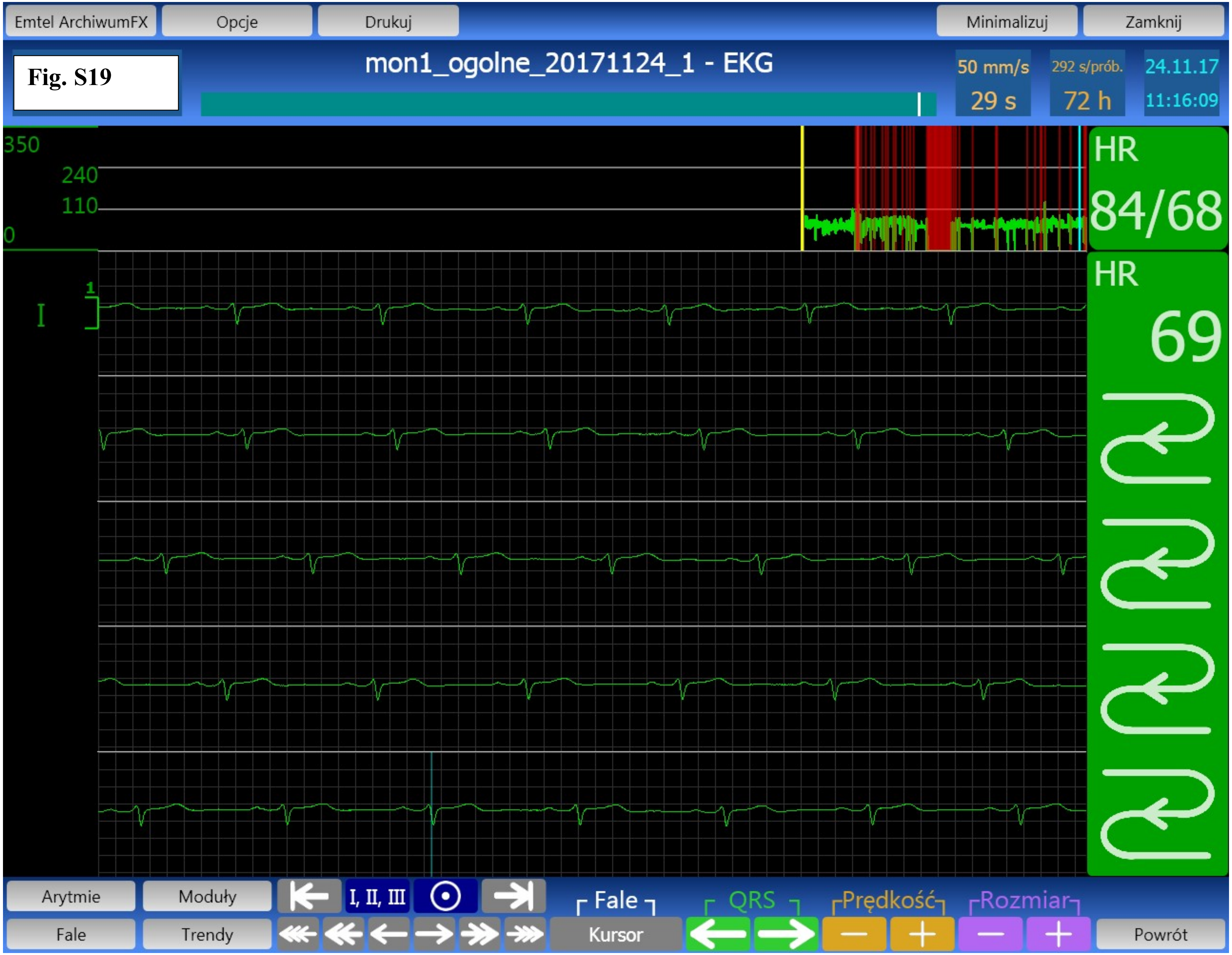
Emtel ArchiwumFX
Opcje
Drukuj
Minimalizuj
Zamknij
Fig. S19
mon1_ogolne_20171124_1 - EKG
50 mm/s
29 s
292 s/prób.
72 h
24.11.17
11:16:09
350
240
110
0
HR
84/68
I
HR
69
Arytmie
Moduły
I, II, III
Fale
QRS
Prędkość
Rozmiar
Fale
Trendy
Kursor
Powrót

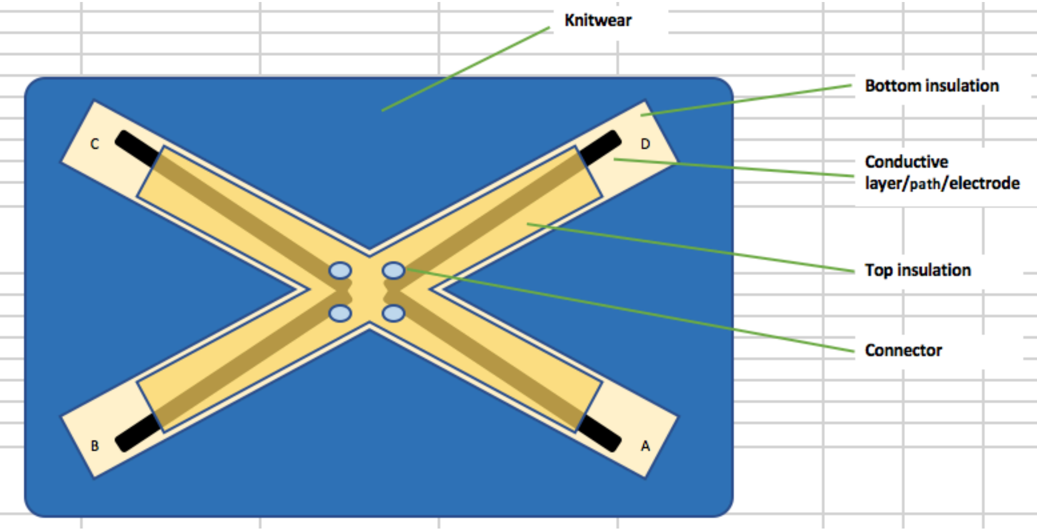

Knitwear	The thickness of the knitted fabric [mm]	thickness measuring point #1	0,338
		thickness measuring point #2	0,362
		thickness measuring point #3	0,327
		thickness measuring point #4	0,35
		thickness measuring point #5	0,355
		thickness measuring point #6	0,344
		thickness measuring point #7	0,335
	**Average thickness of the knitted fabric [mm]**		**0,344**

Bottom insulation	The thickness of the knited fabric with bottom insulation [mm]	thickness measuring point #1	0,446
		thickness measuring point #2	0,412
		thickness measuring point #3	0,431
		thickness measuring point #4	0,421
		thickness measuring point #5	0,425
		thickness measuring point #6	0,452
		thickness measuring point #7	0,455
	**Average thickness of the knited fabric with bottom insulation [mm]**		**0,435**

		Path A	Path B	Path C	Path D
First conductive layer	thickness measuring point #1	0,551	0,517	0,533	0,487
	thickness measuring point #2	0,523	0,538	0,546	0,484
	thickness measuring point #3	0,519	0,559	0,529	0,501
	thickness measuring point #4	0,52	0,549	0,548	0,54
	thickness measuring point #5	0,531	0,533	0,509	0,557
	thickness measuring point #6	0,545	0,549	0,524	0,532
	thickness measuring point #7	0,537	0,515	0,529	0,532
	**Average thickness of the knited fabric and bottom insulation with first layer conductive path [mm]**	**0,532**	**0,537**	**0,531**	**0,519**
	**Resistance of the conductive path after annealing [Ω]**	**332**	**344**	**221**	**355**

		Path A	Path B	Path C	Path D
Second conductive layer	thickness measuring point #1	0,561	0,534	0,538	0,506
	thickness measuring point #2	0,565	0,57	0,596	0,549
	thickness measuring point #3	0,557	0,561	0,559	0,549
	thickness measuring point #4	0,531	0,569	0,565	0,556
	thickness measuring point #5	0,596	0,577	0,55	0,56
	thickness measuring point #6	0,538	0,57	0,547	0,557
	thickness measuring point #7	0,558	0,541	0,563	0,548
	**Average thickness of the knited fabric and bottom insulation with first and second layer of conductive path [mm]**	**0,558**	**0,56**	**0,56**	**0,546**
	**Resistance of the conductive path after annealing [Ω]**	**141**	**143**	**121**	**147**

		Path A	Path B	Path C	Path D
Third conductive layer	thickness measuring point #1	0,593	0,568	0,565	0,578
	thickness measuring point #2	0,575	0,601	0,56	0,584
	thickness measuring point #3	0,568	0,607	0,579	0,572
	thickness measuring point #4	0,552	0,609	0,576	0,597
	thickness measuring point #5	0,601	0,59	0,566	0,588
	thickness measuring point #6	0,596	0,581	0,604	0,559
	thickness measuring point #7	0,596	0,559	0,566	0,532
	**Average thickness of the knited fabric and bottom insulation with first, second and third layer of conductive path [mm]**	**0,583**	**0,588**	**0,574**	**0,573**
	**Resistance of the conductive path after annealing [Ω]**	**84**	**94**	**76**	**97**

		Path A	Path B	Path C	Path D
First layer of top insulation	thickness measuring point #1	0,573	0,537	0,589	0,555
	thickness measuring point #2	0,579	0,584	0,62	0,565
	thickness measuring point #3	0,596	0,592	0,569	0,618
	thickness measuring point #4	0,553	0,576	0,556	0,561
	thickness measuring point #5	0,603	0,594	0,577	0,561
	thickness measuring point #6	0,61	0,565	0,561	0,573
	thickness measuring point #7	0,534	0,578	0,571	0,56
	**Average thickness of the knited fabric and bottom insulation with first, second and third layer of conductive path and first layer of top insulation [mm]**	**0,578**	**0,575**	**0,578**	**0,57**
	**Resistance of the insulation after annealing [Ω]**	**124**	**326**	**204**	**182**
	**Resistance between of the conductive path and insulation after annealing [Ω]**	96<br>95	116<br>98	84<br>170	420<br>282
	**Average resistance between of the conductive path and insulation after annealing [Ω]**	**96**	**107**	**127**	**351**

		Path A	Path B	Path C	Path D
**Second layer of top insulation**	thickness measuring point #1	0,632	0,633	0,655	0,634
	thickness measuring point #2	0,654	0,694	0,68	0,661
	thickness measuring point #3	0,659	0,681	0,626	0,67
	thickness measuring point #4	0,633	0,659	0,65	0,654
	thickness measuring point #5	0,659	0,654	0,667	0,663
	thickness measuring point #6	0,663	0,631	0,632	0,621
	thickness measuring point #7	0,577	0,641	0,64	0,666
	**Average thickness of the knited fabric and bottom insulation with first, second and third layer of conductive path and two layers of top insulation [mm]**	**0,64**	**0,656**	**0,65**	**0,653**
	**Resistance of the insulation after annealing [Ω]**	**38 000 000**	**11 000 000**	**114 000**	**2 700 000**
	**Resistance between of the conductive path and insulation after annealing**	11 000 000<br>11 000 000	1 100 000<br>4 300 000	2 500 000<br>5 000 000	5 000 000<br>2 000 000
	**Average resistance between of the conductive path and insulation after annealing [Ω]**	**11 000 000**	**2 700 000**	**3 750 000**	**3 500 000**

		Path A	Path B	Path C	Path D
Third layer of top insulation	thickness measuring point #1	0,624	0,641	0,697	0,646
	thickness measuring point #2	0,647	0,682	0,678	0,664
	thickness measuring point #3	0,686	0,685	0,671	0,696
	thickness measuring point #4	0,606	0,695	0,618	0,68
	thickness measuring point #5	0,577	0,672	0,688	0,68
	thickness measuring point #6	0,675	0,672	0,647	0,676
	thickness measuring point #7	0,636	0,666	0,64	0,675
	**Average thickness of the knited fabric and bottom insulation with first, second and third layer of conductive path and three layers of top insulation [mm]**	**0,636**	**0,673**	**0,663**	**0,674**
	**Resistance of the insulation after annealing [Ω]**	**out of measuring range**	**out of measuring range**	**out of measuring range**	**out of measuring range**
	**Resistance between of the conductive path and insulation after annealing [Ω]**	out of measuring range<br>out of measuring range	out of measuring range<br>out of measuring range	out of measuring range<br>out of measuring range	out of measuring range<br>out of measuring range
	**Average resistance between of the conductive path and insulation after annealing [Ω]**	**out of measuring range**	**out of measuring range**	**out of measuring range**	**out of measuring range**

		Path A	Path B	Path C	Path D
T-shirt ready for ECG registration	**Resistance between the electrode and connector after final annealing [Ω]**	116	101	112	97

Fig. S20-S21 Scheme of the central T-shirt textronics (top), and the corresponding gradually changing parameters (thickness, electroconductivity) over the successive preparation of the functional ECG T-shirt (bottom)

Fig. S22-S25 Examination of the fitness T-shirt in the configuration (**I**) (from **Fig. 5A**) using a generator of real signals collected from the CSE (Common Standards for Quantitative Electrocardiography) base signal with a small amplitude of the QRS-complex (**Fig. S24**), and using a generator of the reference ECG signals collected from the CTS base with an amplitude of 1 mV for the R-wave (**Fig. S25**). In both cases, the behavior of the conductive connections applied to the fitness T-shirt showed excellent transmission properties.

Fig. S22

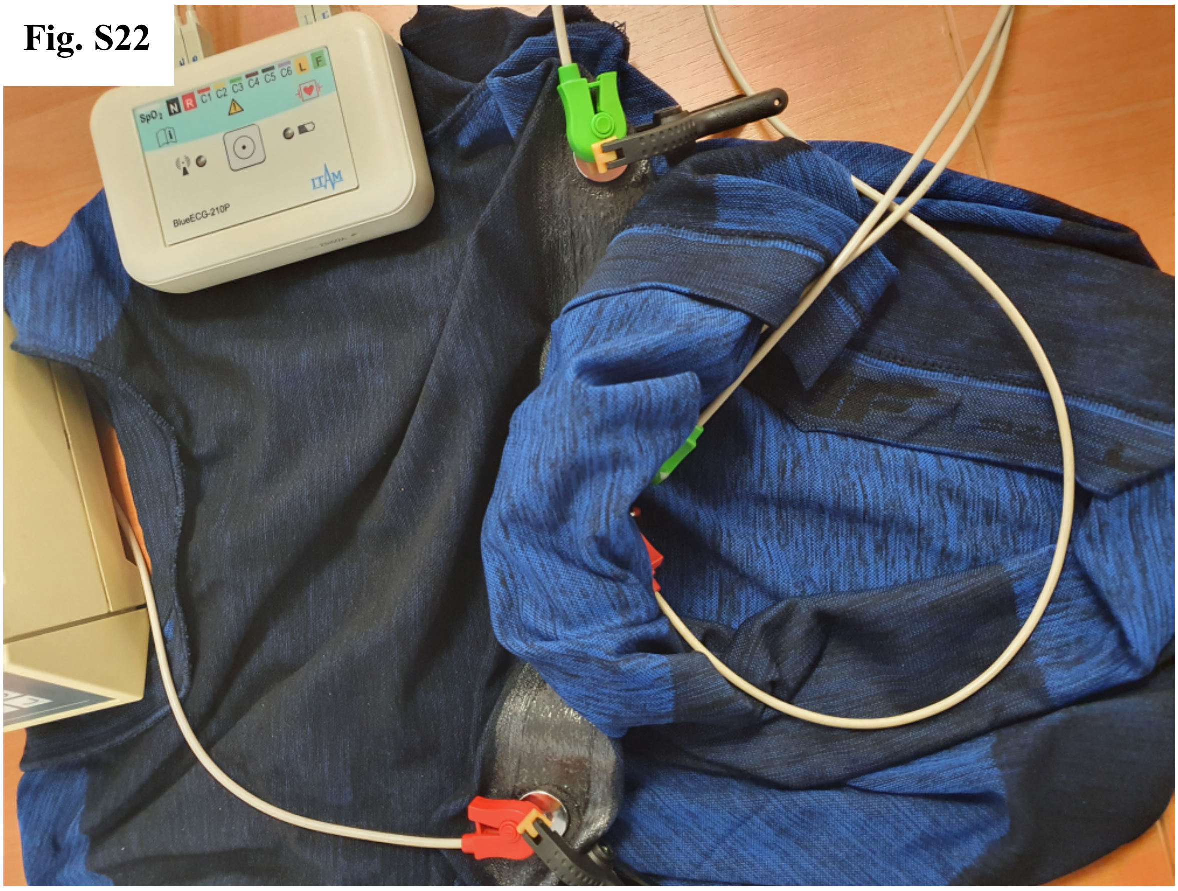

Fig. S23

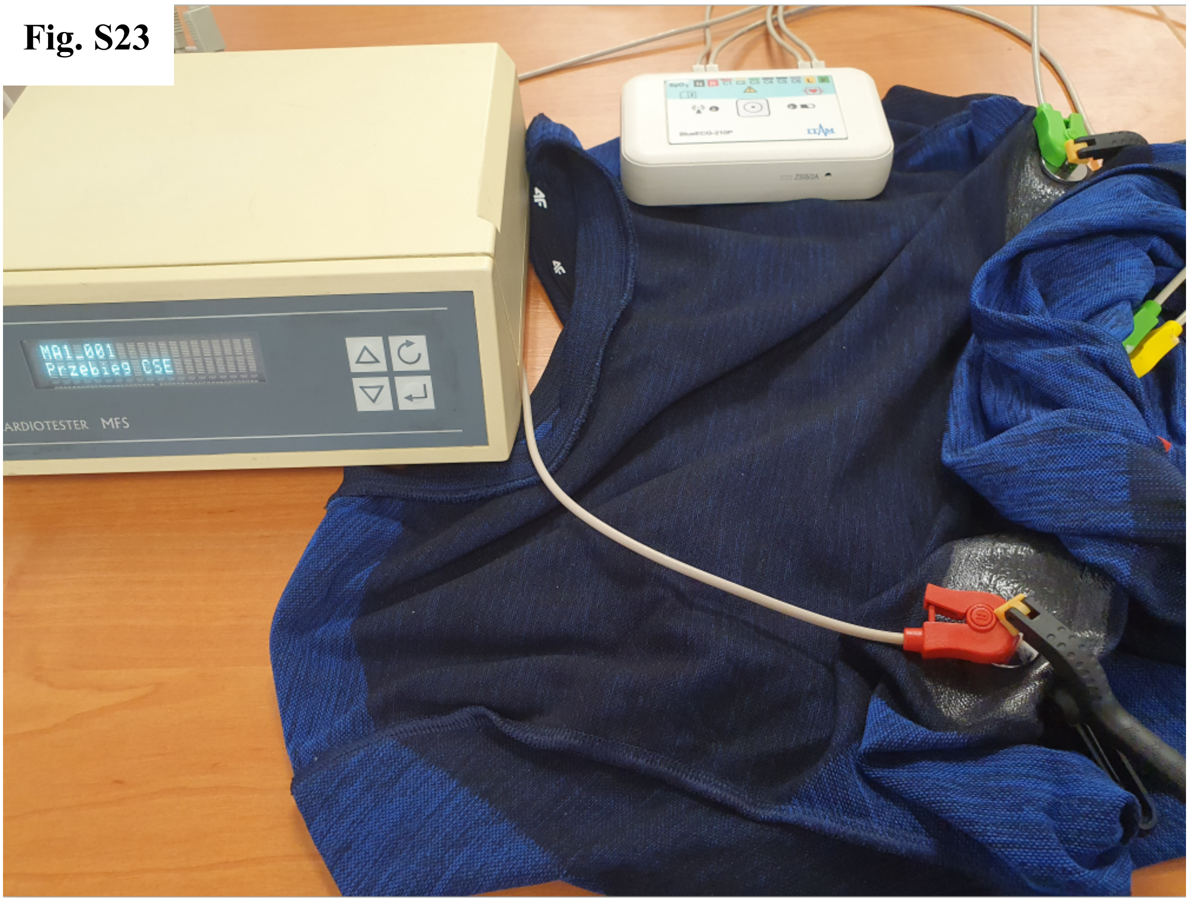

Fig. S24

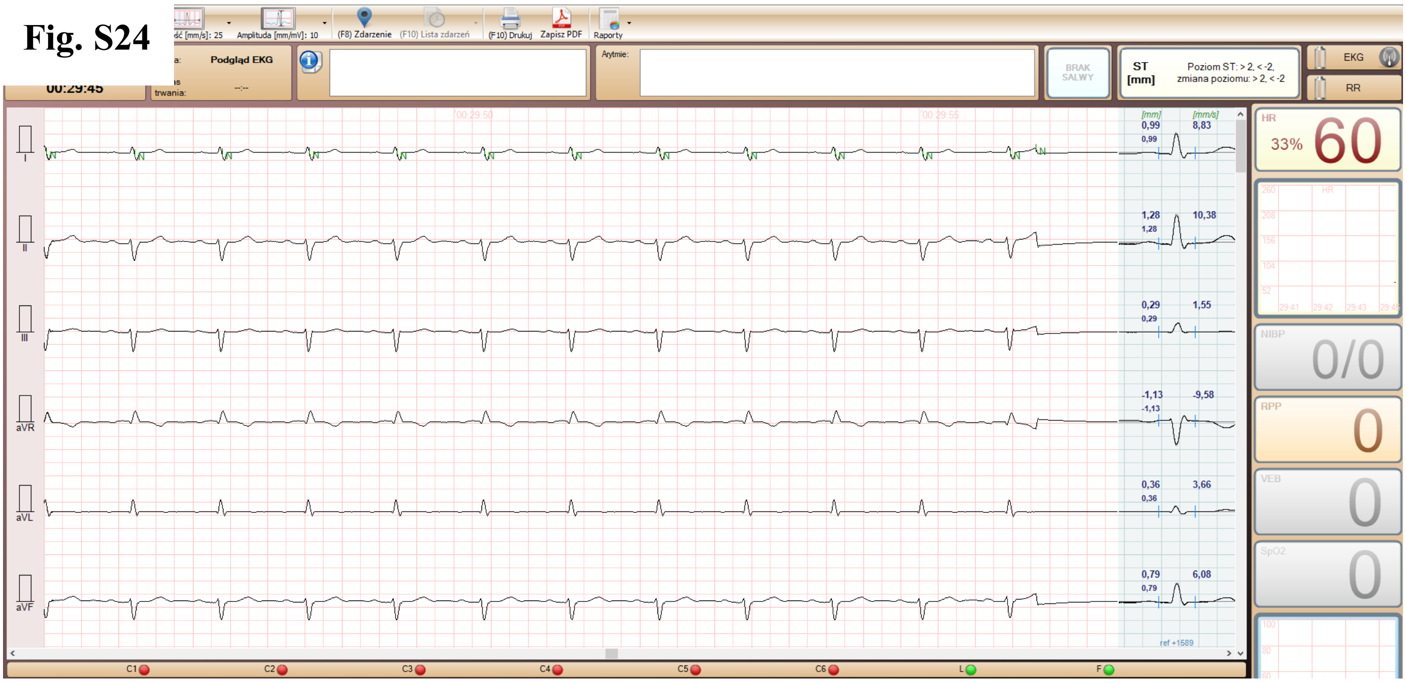

Fig. S25

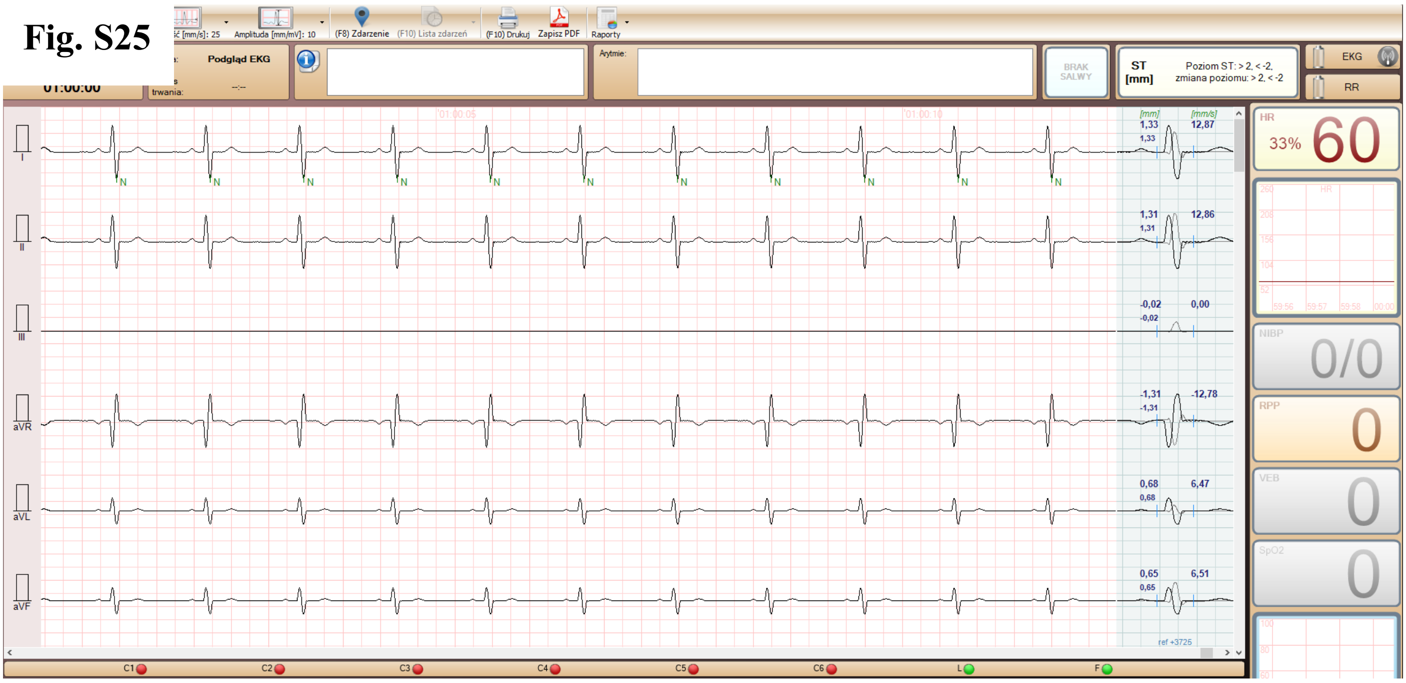

Amplituda [mm/mV]: 10
(F8) Zdarzenie
(F10) Lista zdarzeń
(F10) Drukuj
Zapisz PDF
Raporty
Podgląd EKG
01:00:00
trwania:
Arytmie:
BRAK SALWY
ST [mm]
Poziom ST: > 2, < -2, zmiana poziomu: > 2, < -2
EKG
RR
I
II
III
aVR
aVL
aVF
[mm]
[mm/s]
1,33
12,87
1,31
12,86
-0,02
0,00
-1,31
-12,78
0,68
6,47
0,65
6,51
HR
33%
60
NIBP
0/0
RPP
0
VEB
0
SpO2
0
C1
C2
C3
C4
C5
C6
L
F

Fig. S26-S31 Photographs from the examination of the first long-term T-shirt in the configuration (**II**) (from **Fig. 5A**) using the ECG reference signal generator taken from the CTS database with an amplitude of 1 mV for the R-wave and a variable repetition period of the characteristic PQRST-complexes corresponding to the heart rates respectively of 60, 40 and 120 min^{-1} (**Figs. S29, S30, S31**). Also, in this case, the recorded ECG signals with variable rhythm were found stable, undisturbed, and repeatable throughout the recording upon testing the T-shirt with phantom-generated ECG signals in the configuration (**II**)

Fig. S26

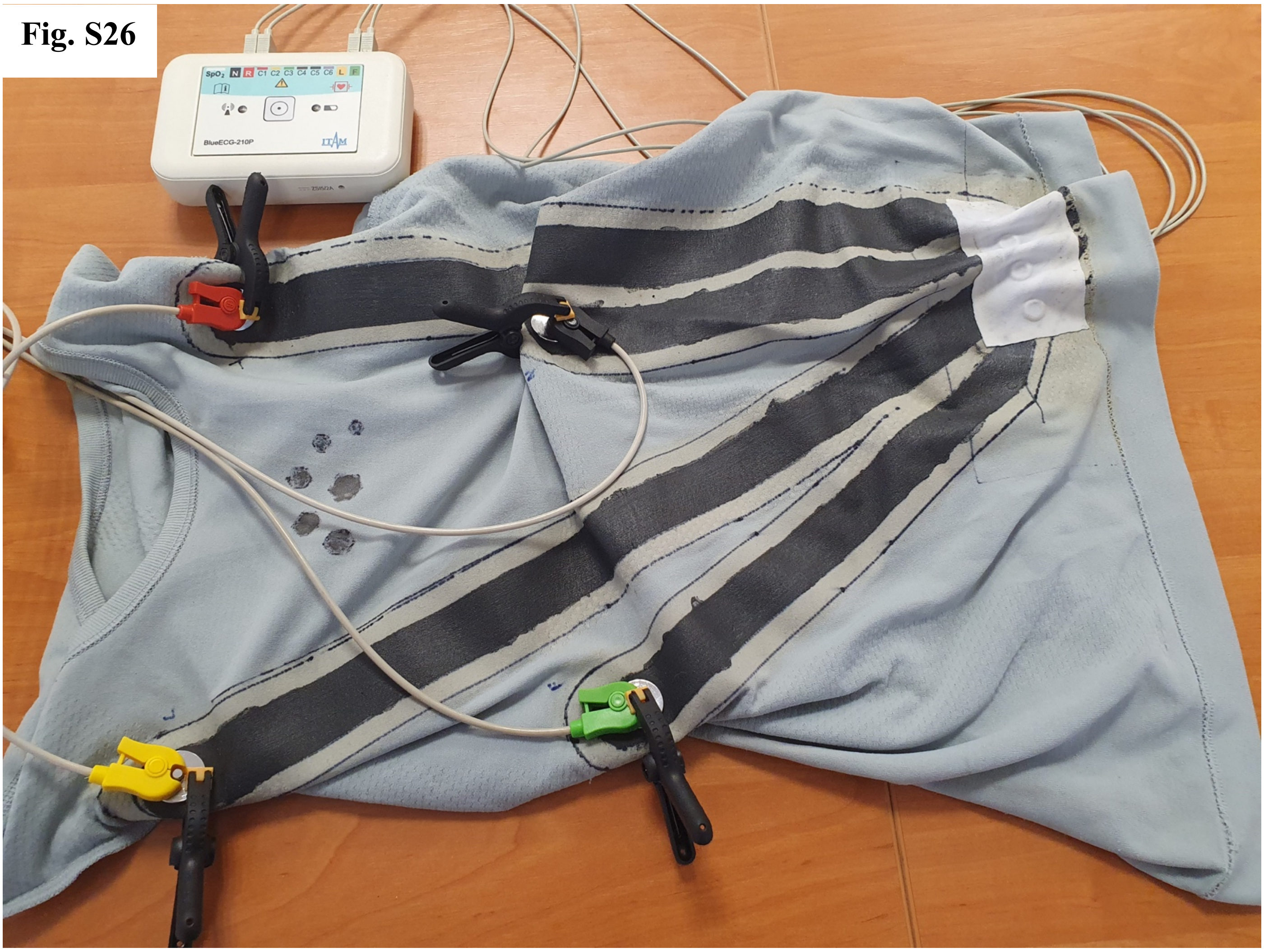

Fig. S27

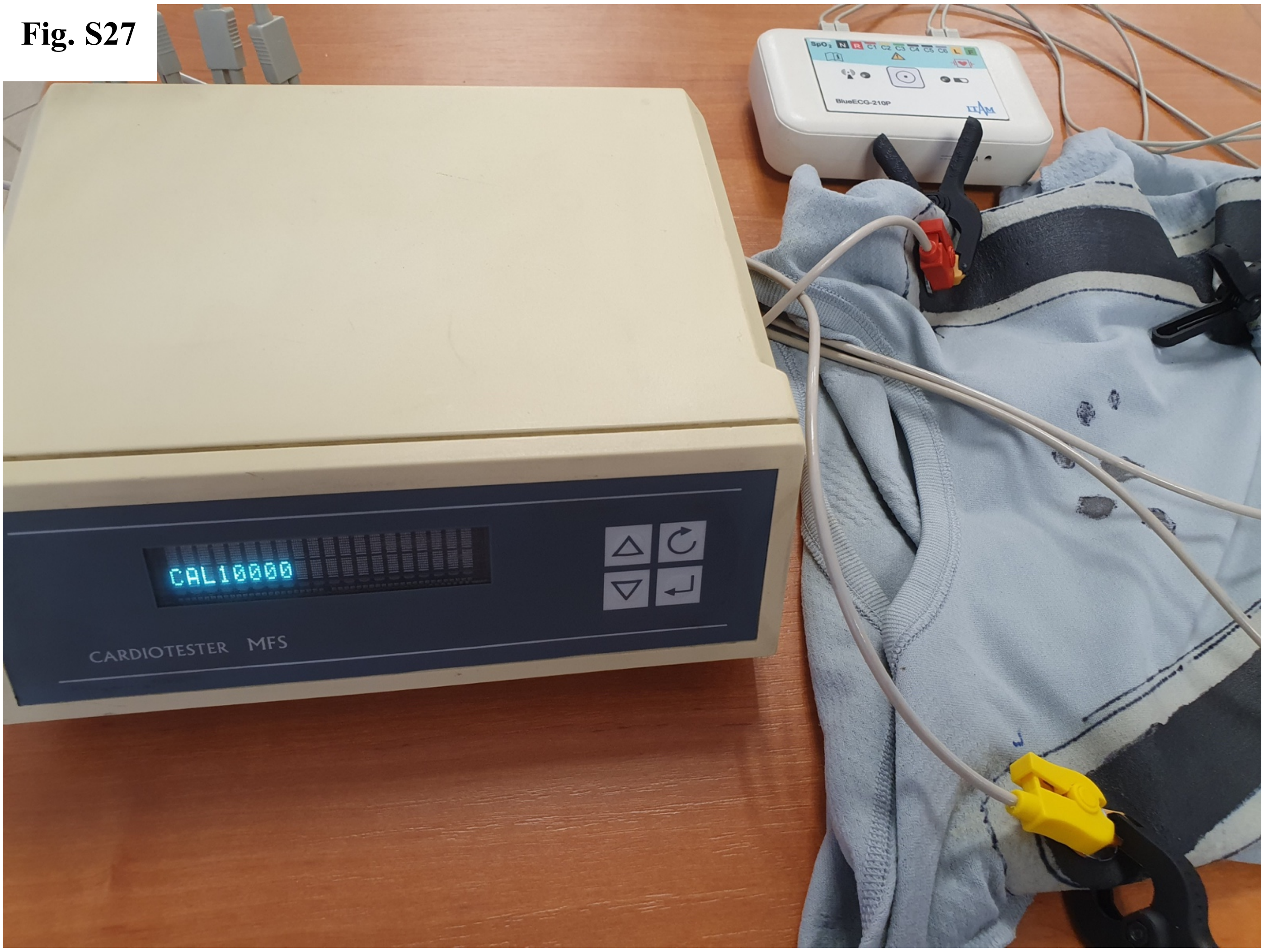

Fig. S28

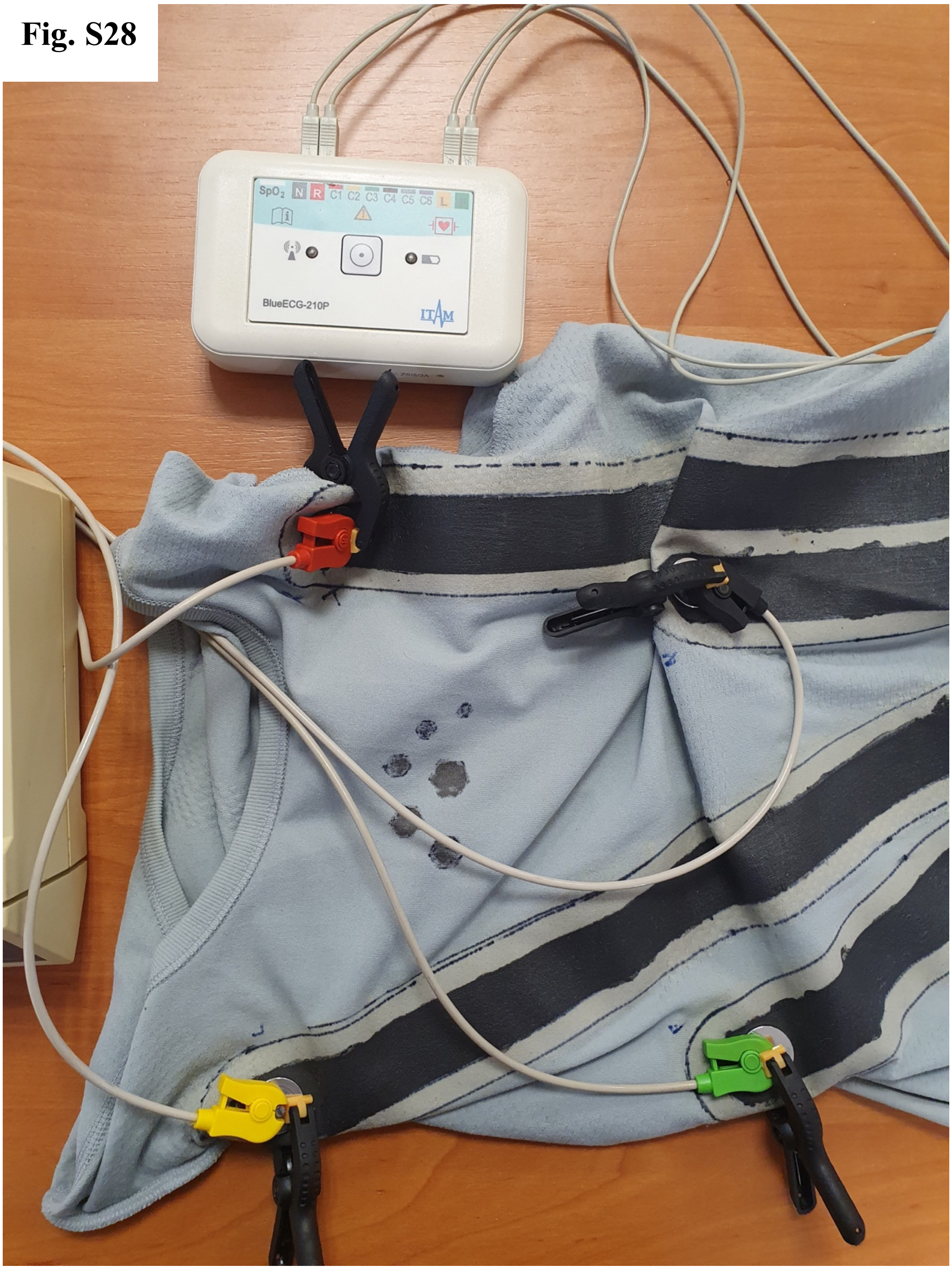

Fig. S29

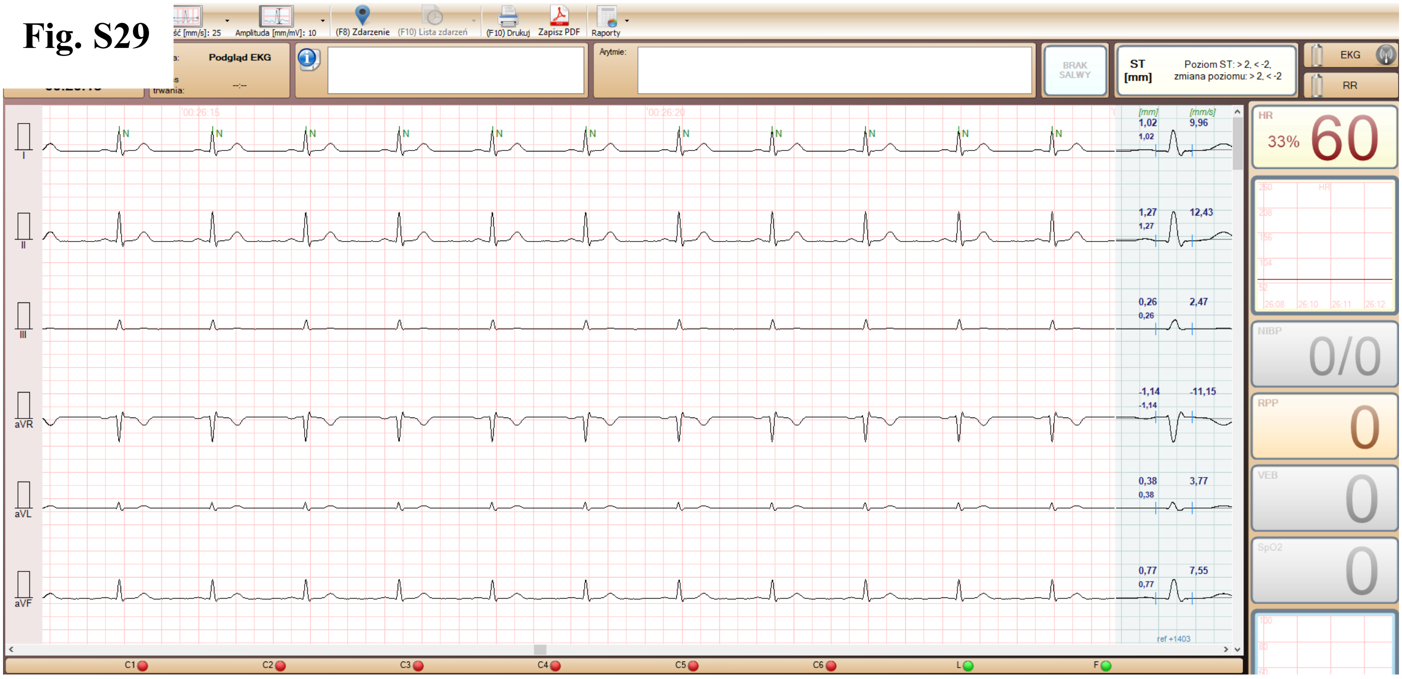

Podgląd EKG
Arytmie:
BRAK SALWY
ST [mm]
Poziom ST: > 2, < -2, zmiana poziomu: > 2, < -2
EKG
RR
HR
33%
60
NIBP
0/0
RPP
0
VEB
0
SpO2
0
(F8) Zdarzenie
(F10) Lista zdarzeń
(F10) Drukuj
Zapisz PDF
Raporty
I
II
III
aVR
aVL
aVF
C1
C2
C3
C4
C5
C6
L
F

Fig. S30

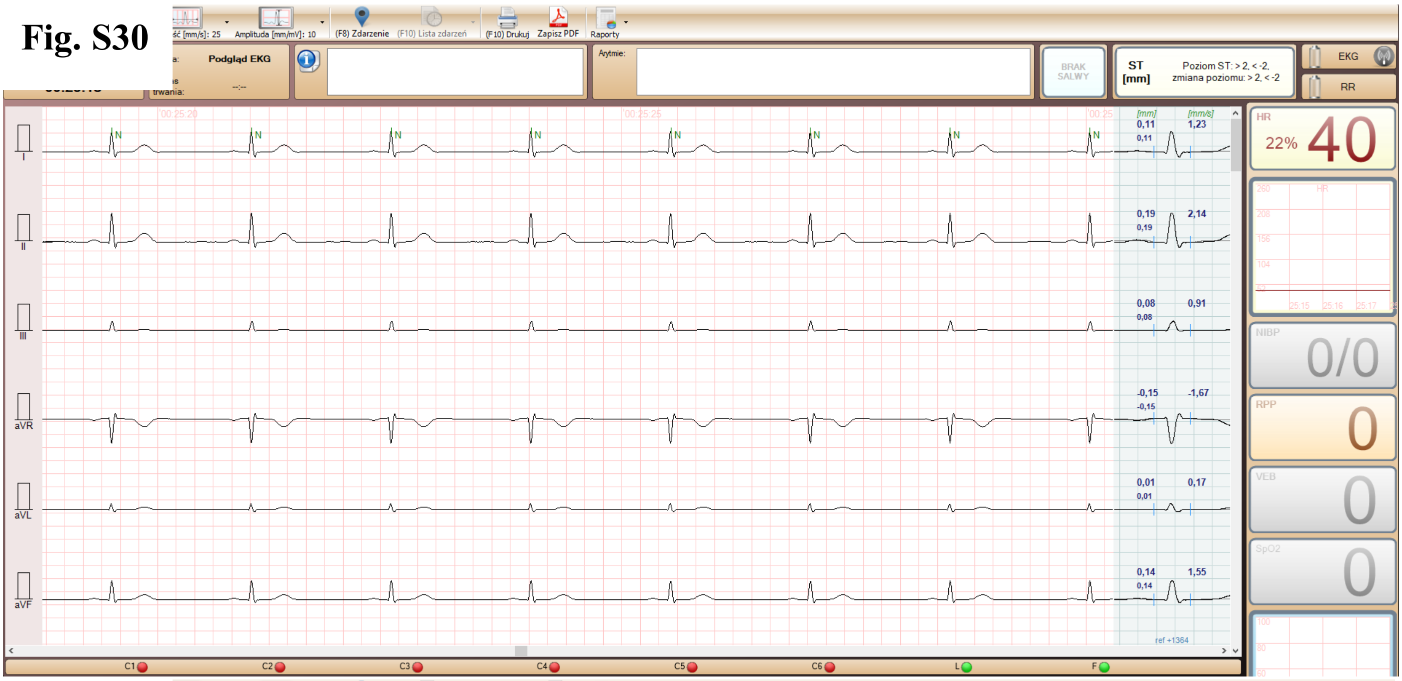

Fig. S31

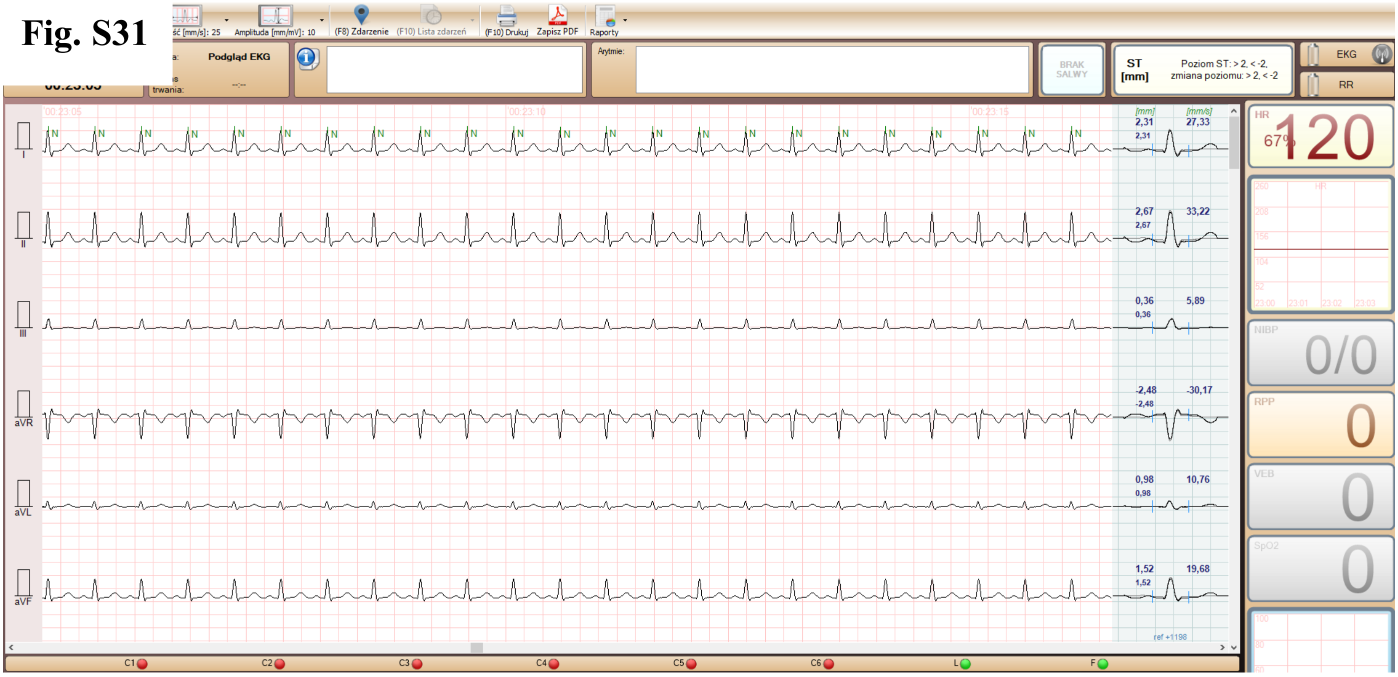

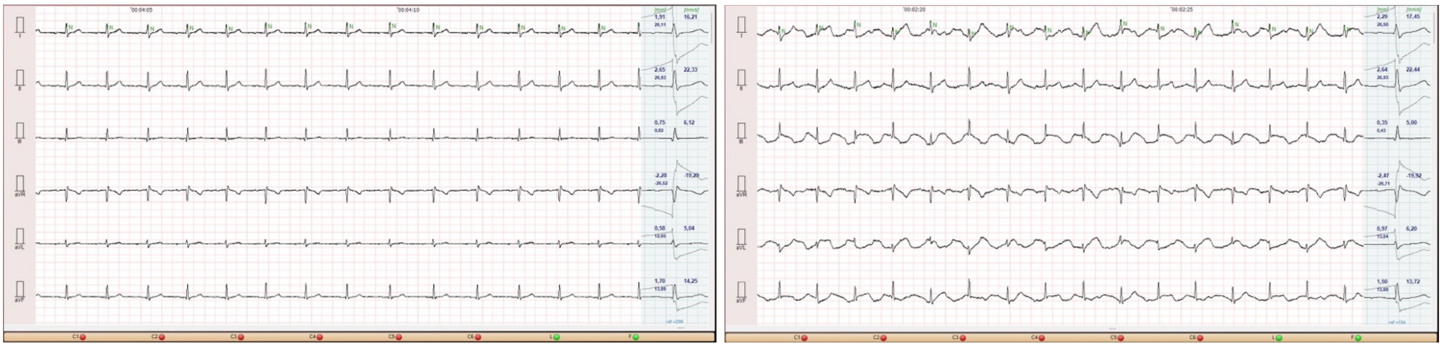

Fig. S32 ECG signal acquired for the healthy 50-yrs old male (S size) upon sitting (*left*) and movement (*right*) acquired using standard Ag/AgCl electrodes

Photographs from testing the T-shirt with phantom-generated ECG signals in the configuration (**IV**): **Fig. S33-S34** the final/optimized geometry of the T-shirt in the configuration (**IV**) (from **Fig. 5A**); **Fig. S35** presents a wearable medical T-shirt with a target model of a two-channel ECG recorder allowing a simultaneous recording of the respiratory parameters, including respiratory rate (this will significantly increase the diagnostic value of the recorded signals and expand the range of applications, e.g. for cardiac rehabilitation, but also for diagnosing respiratory functions, and monitoring the respiratory cycles of patients e.g. suffering from sleep apnea).

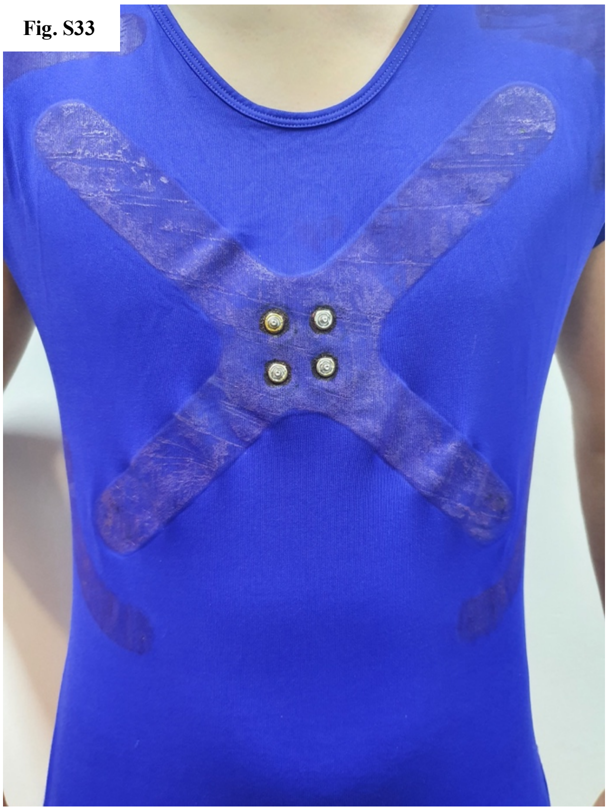

Fig. S33

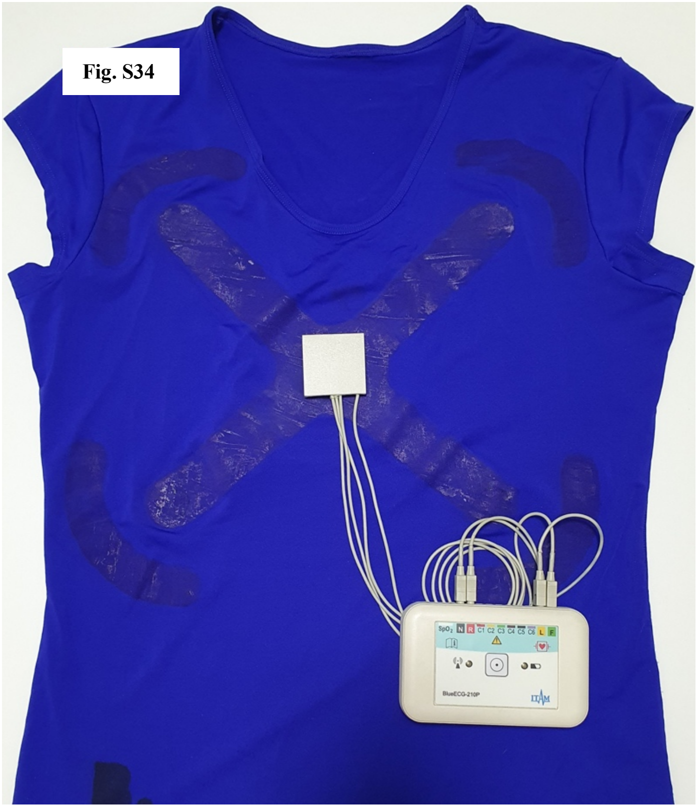

Fig. S34

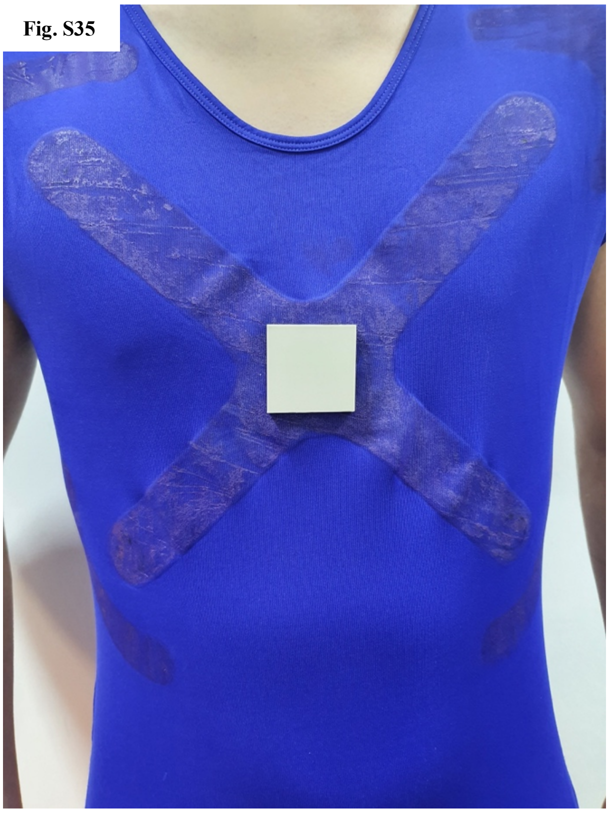

Fig. S35

Fig. S36-S48 show records of the exercise test during treadmill walking, the heart rate (HR) of which from 69 min^{-1} in 3 min became 122 min^{-1} while maintaining the unchanged diagnostic quality of the ECG signals; with a further giving up the physical activity

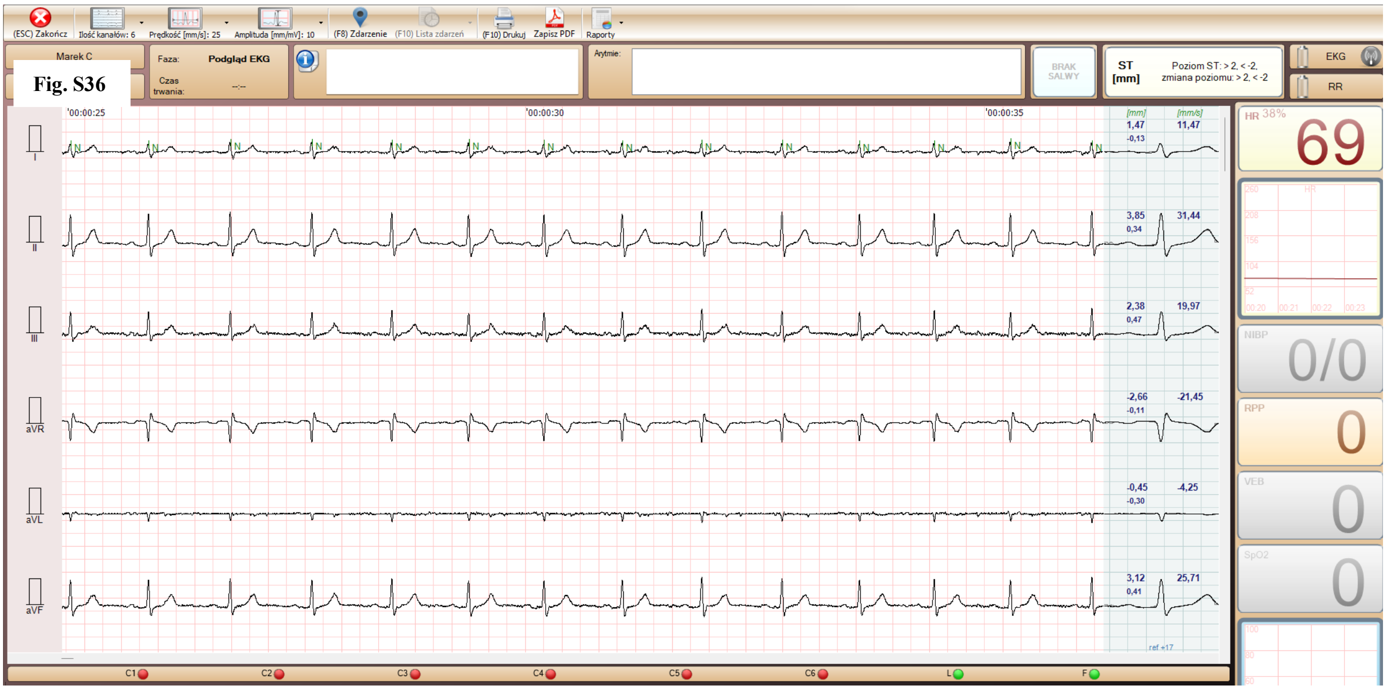

Fig. S36

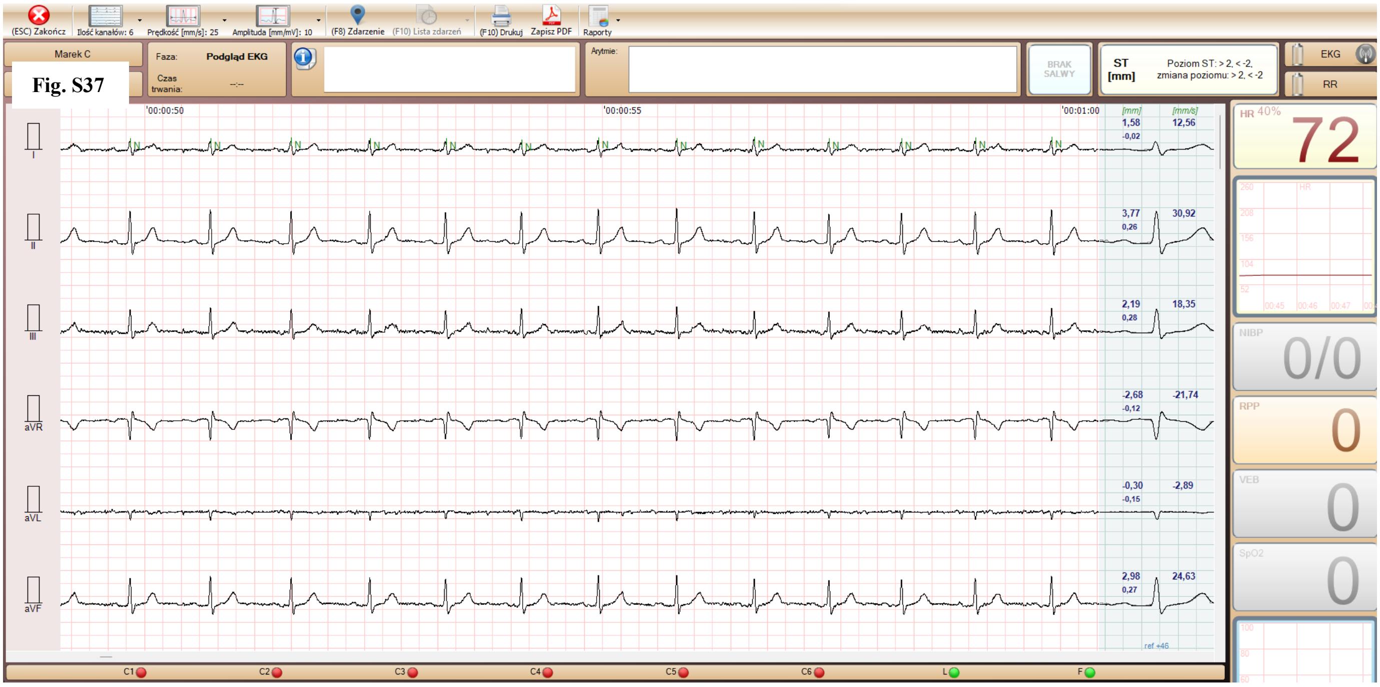

Fig. S37

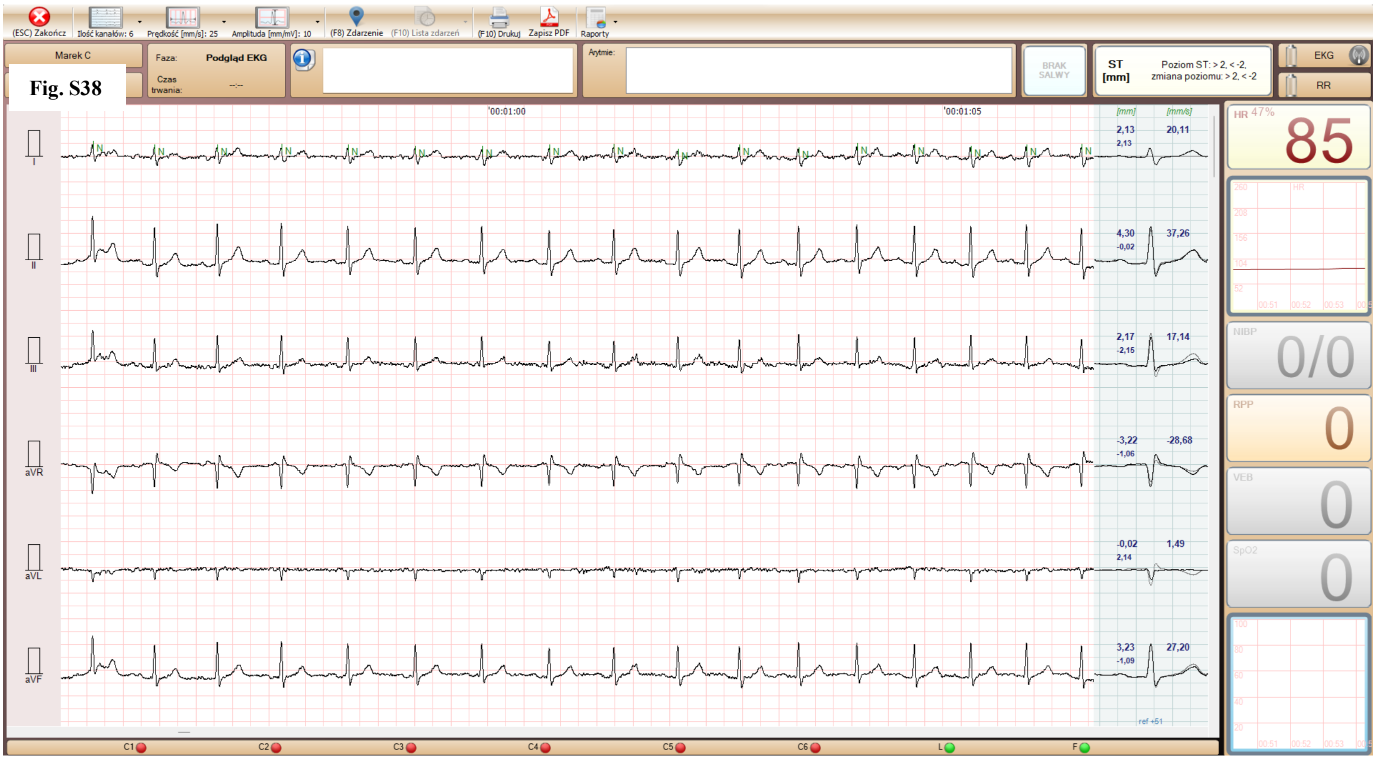

Fig. S38

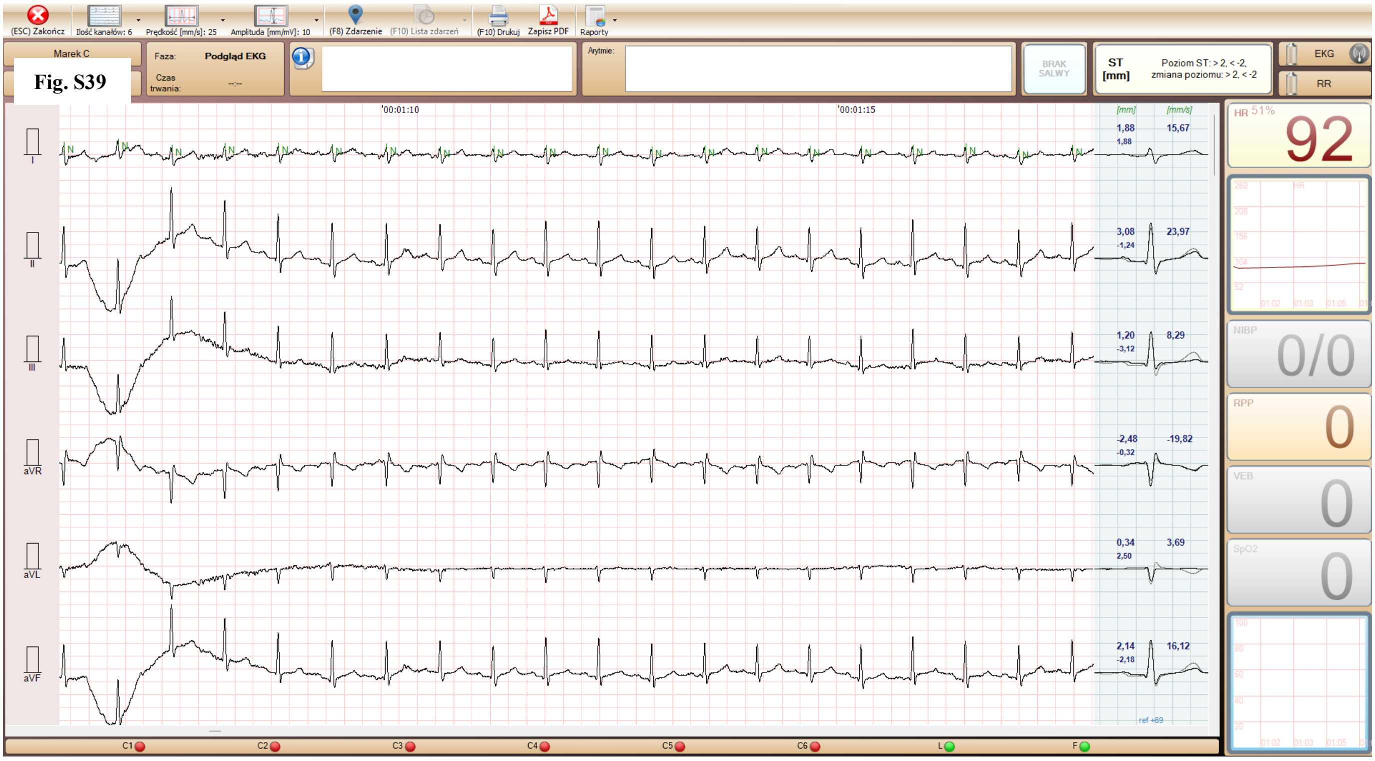

Fig. S39

Fig. S40

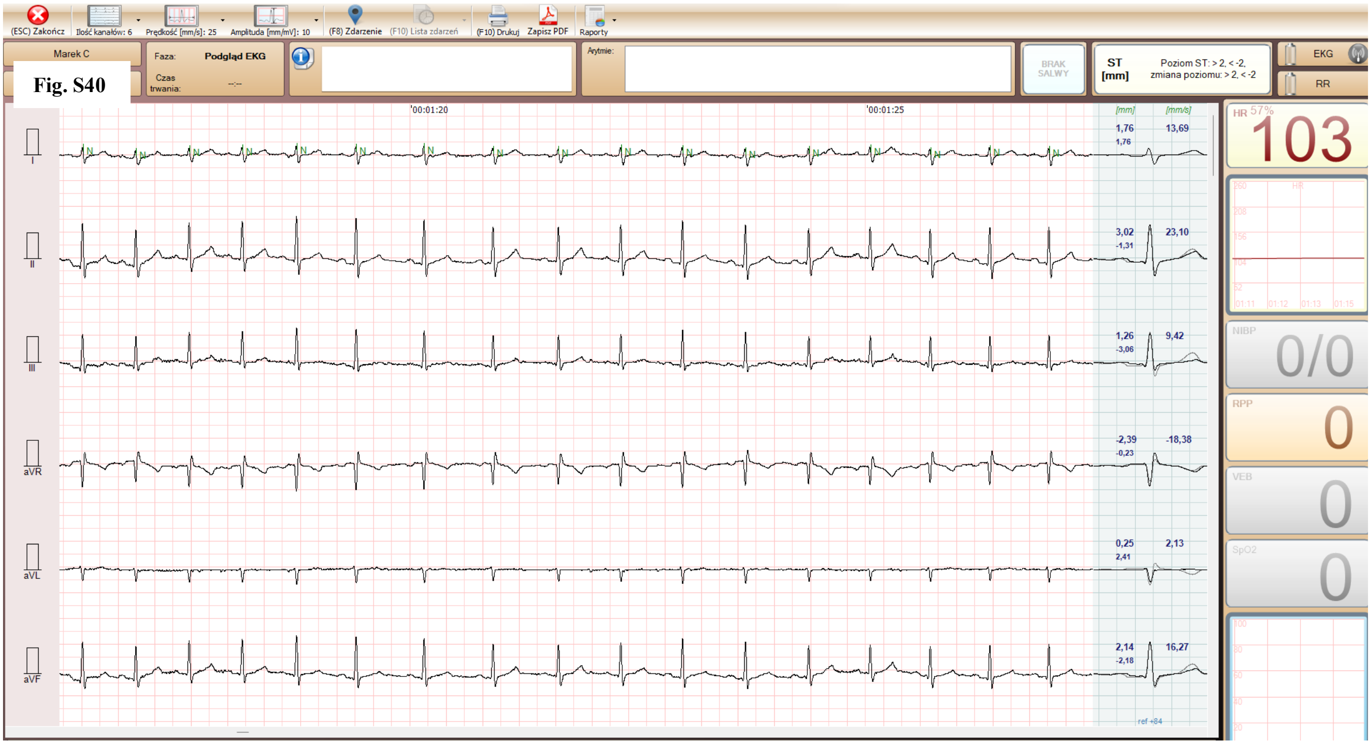

Fig. S41

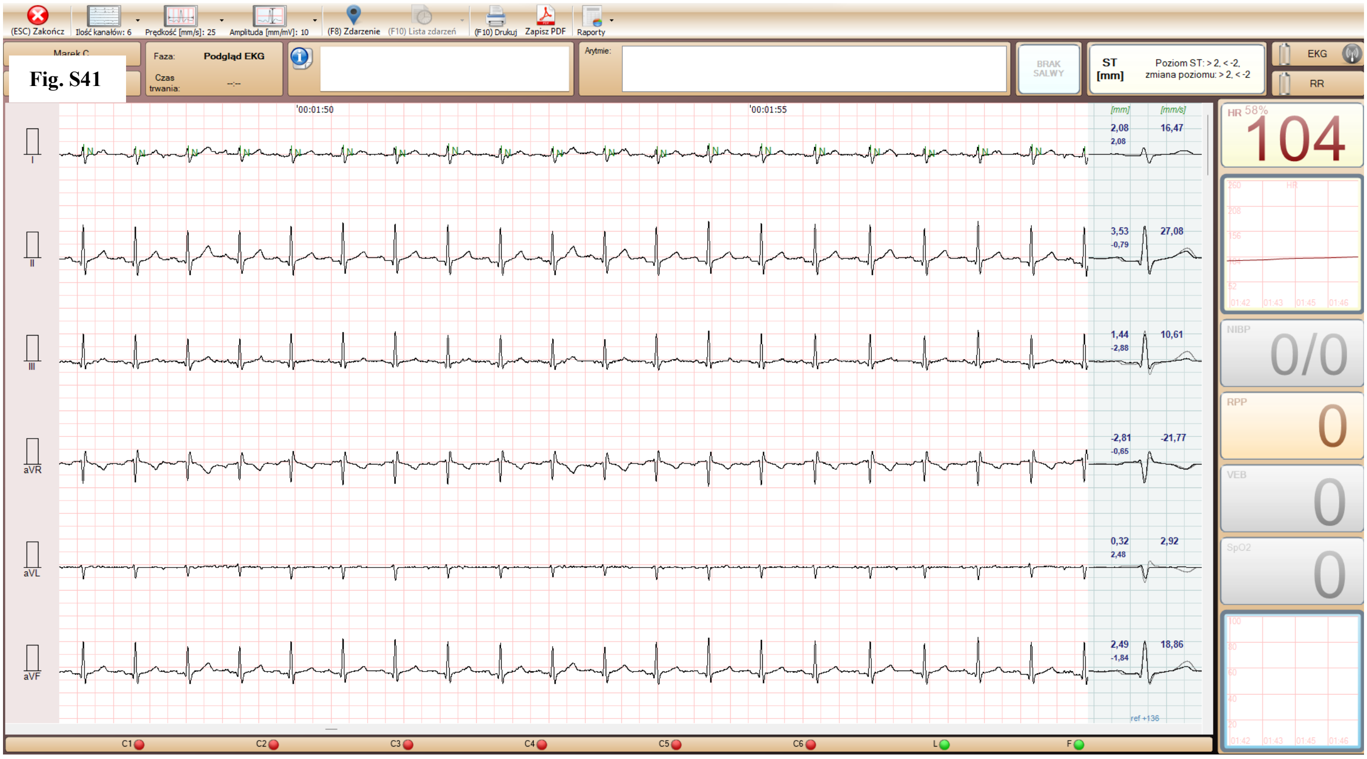

Fig. S42

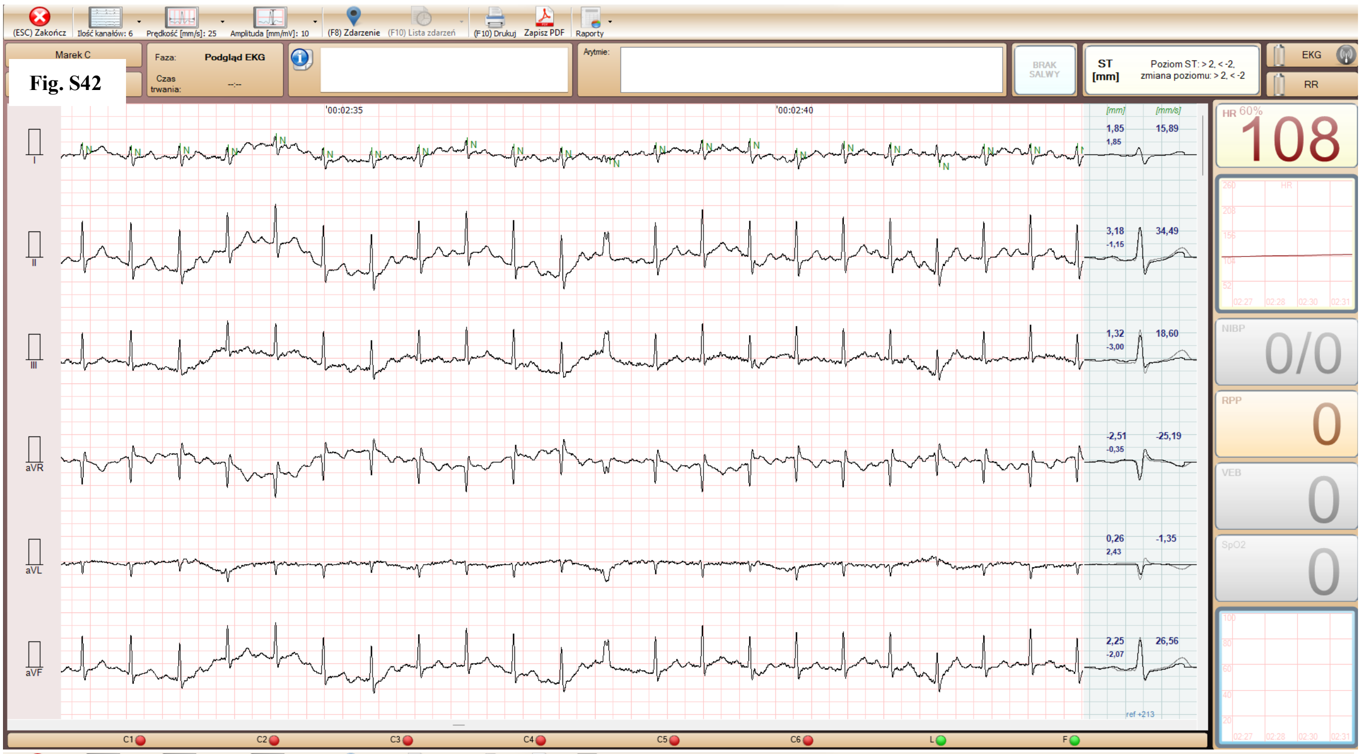

Fig. S43

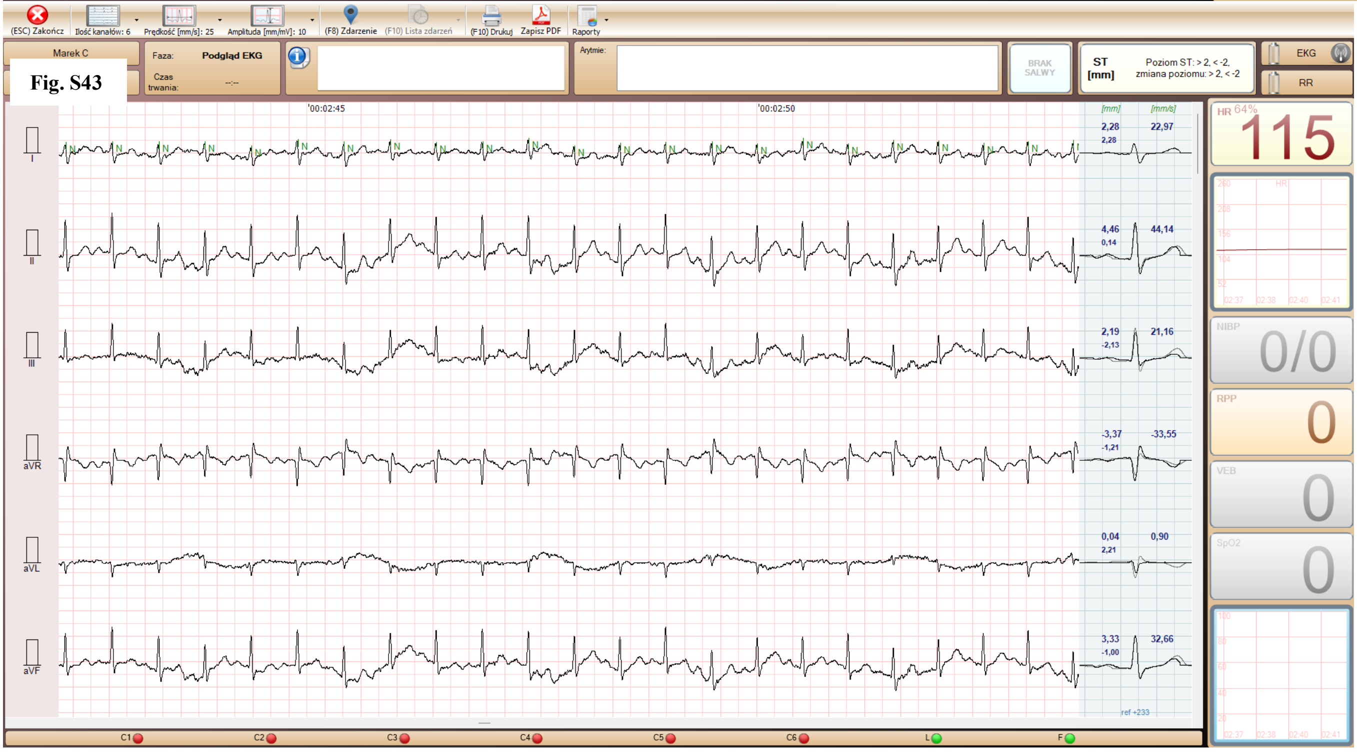

Fig. S44

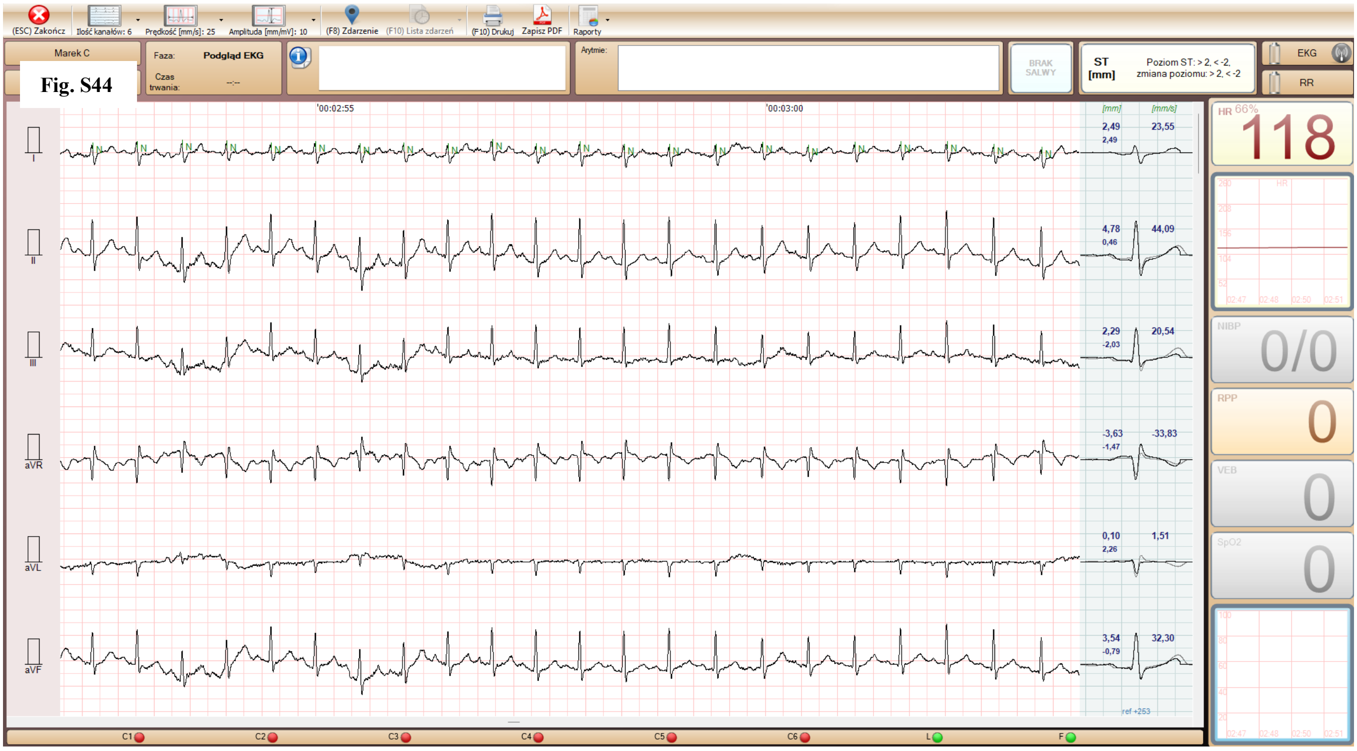

(ESC) Zakończ
Ilość kanałów: 6
Prędkość [mm/s]: 25
Amplituda [mm/mV]: 10
(F8) Zdarzenie
(F10) Lista zdarzeń
(F10) Drukuj
Zapisz PDF
Raporty
Marek C
Faza:
Podgląd EKG
Czas trwania:
Arytmie:
BRAK SALWY
ST [mm]
Poziom ST: > 2, < -2, zmiana poziomu: > 2, < -2
EKG
RR
'00:02:55
'00:03:00
[mm]
[mm/s]
I
2,49
23,55
2,49
II
4,78
44,09
0,46
III
2,29
20,54
-2,03
aVR
-3,63
-33,83
-1,47
aVL
0,10
1,51
2,26
aVF
3,54
32,30
-0,79
ref +253
HR 66%
118
NIBP
0/0
RPP
0
VEB
0
SpO2
0
C1
C2
C3
C4
C5
C6
L
F

Fig. S45

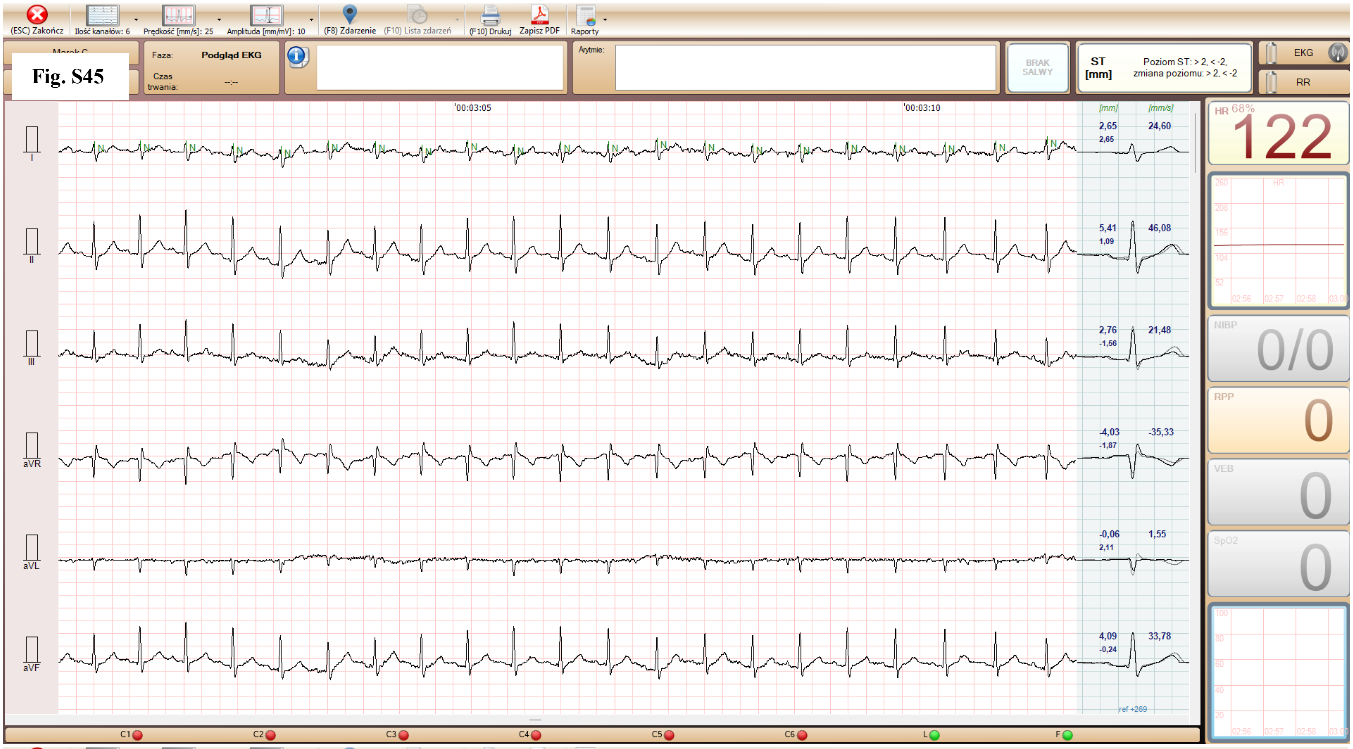

Fig. S46

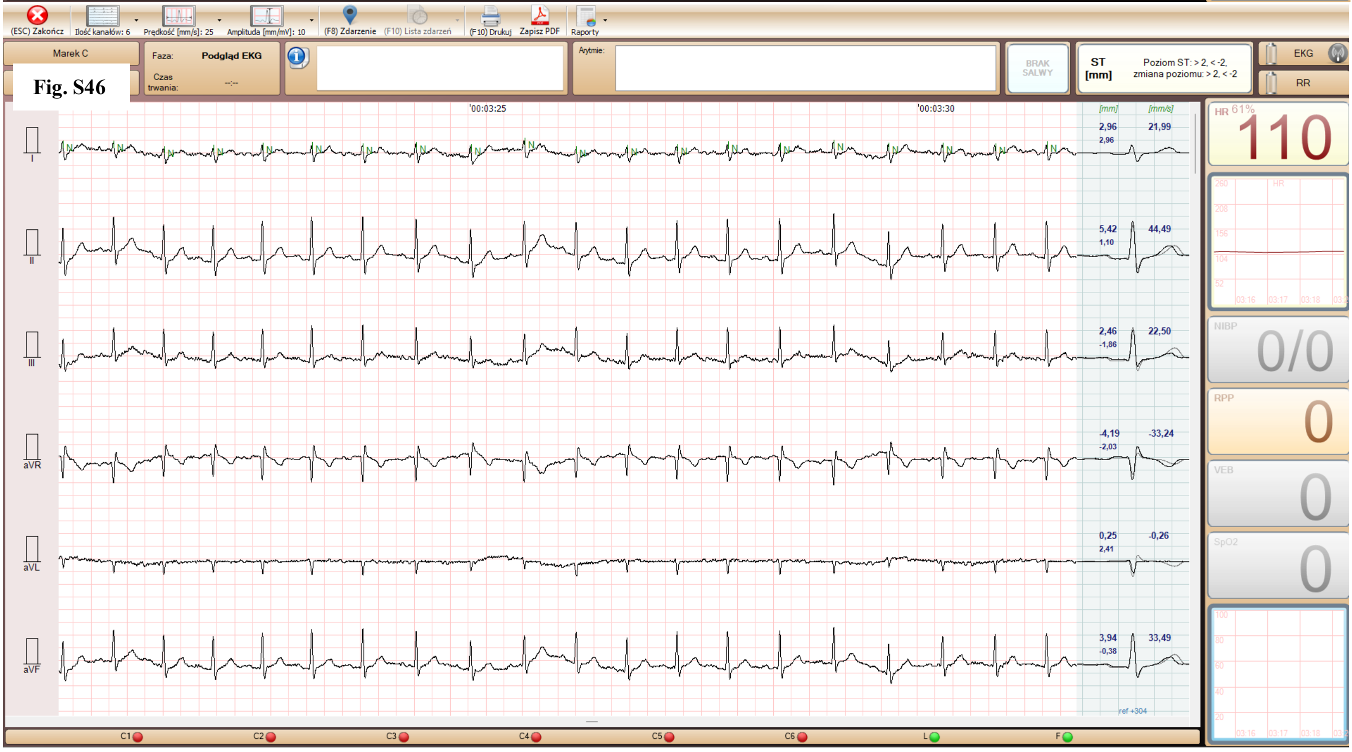

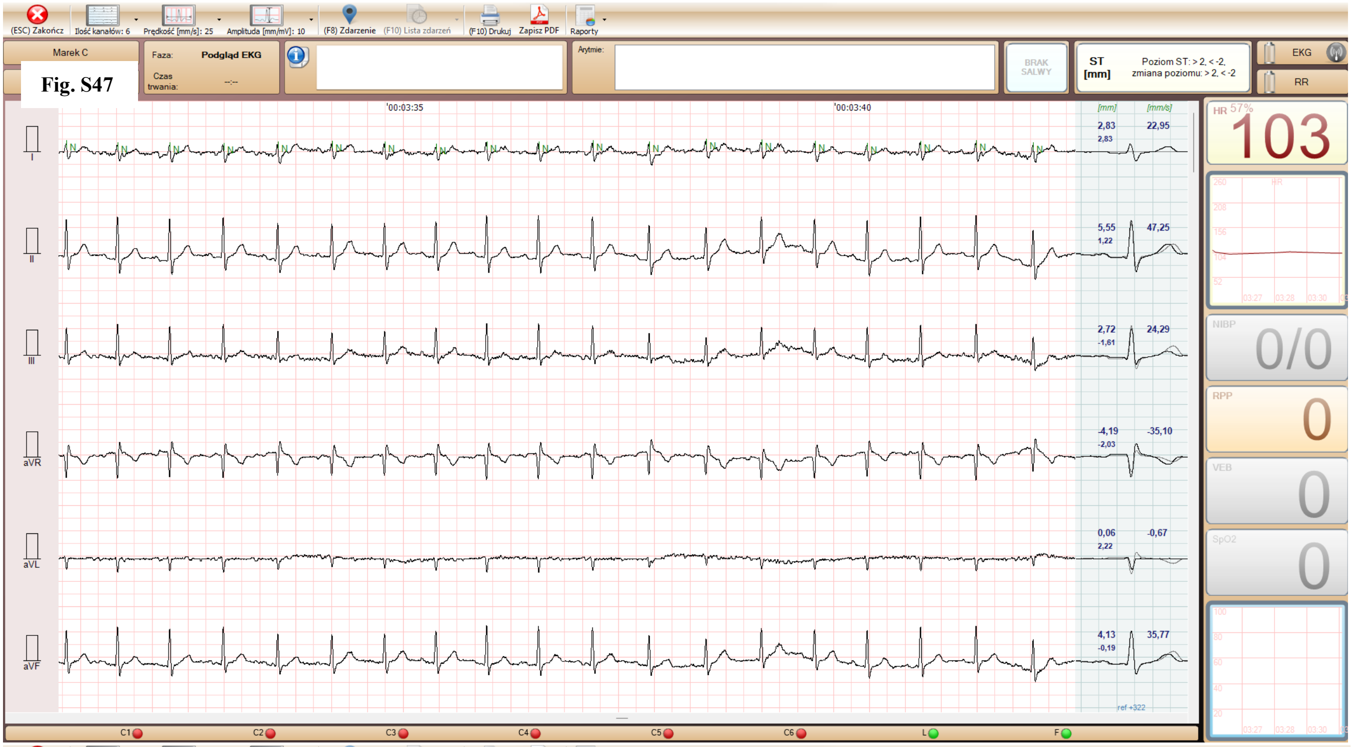

Fig. S47

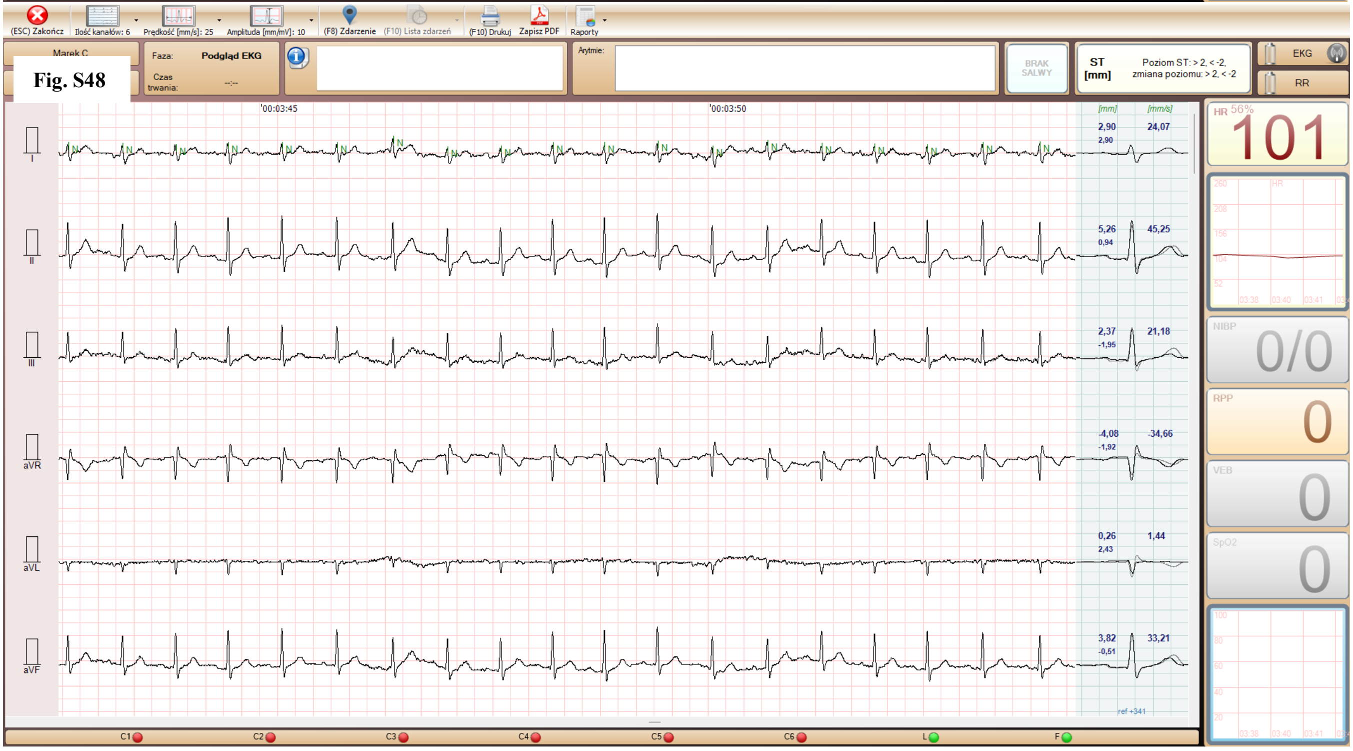

Fig. S48

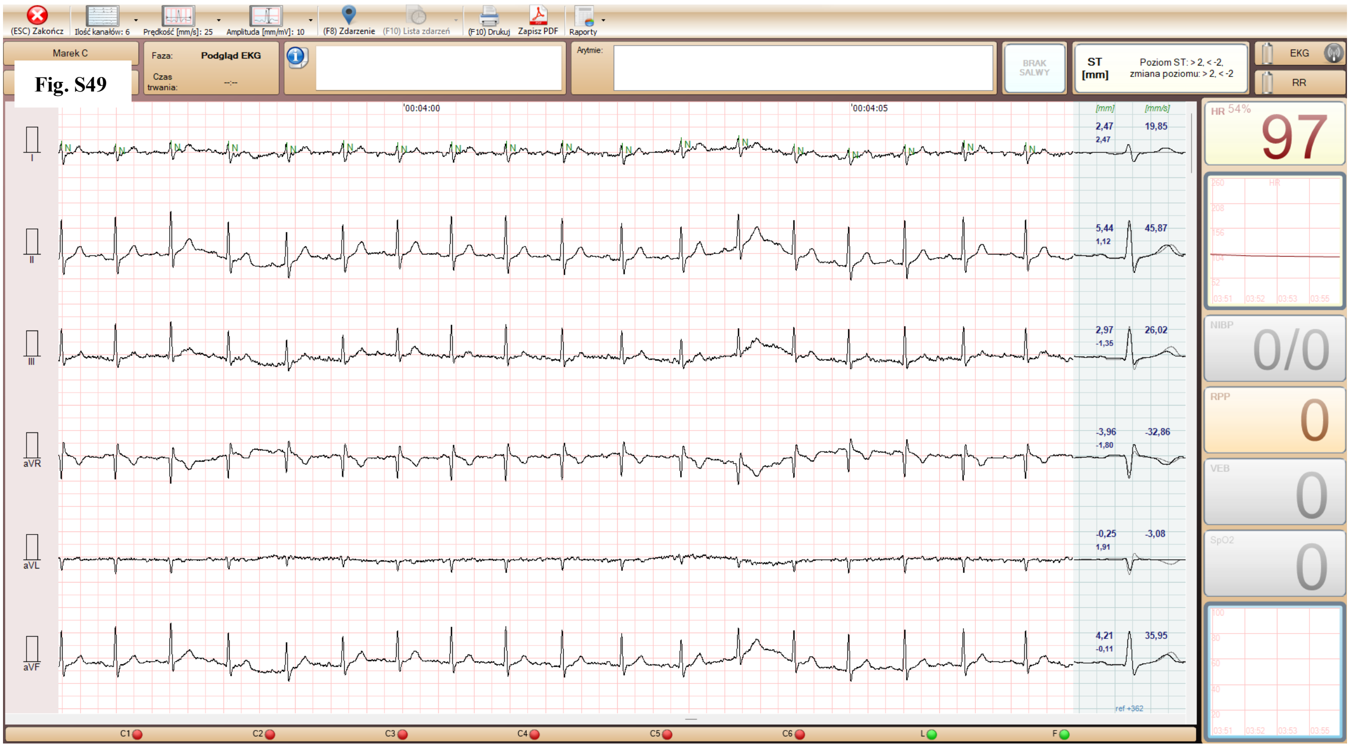

Fig. S49

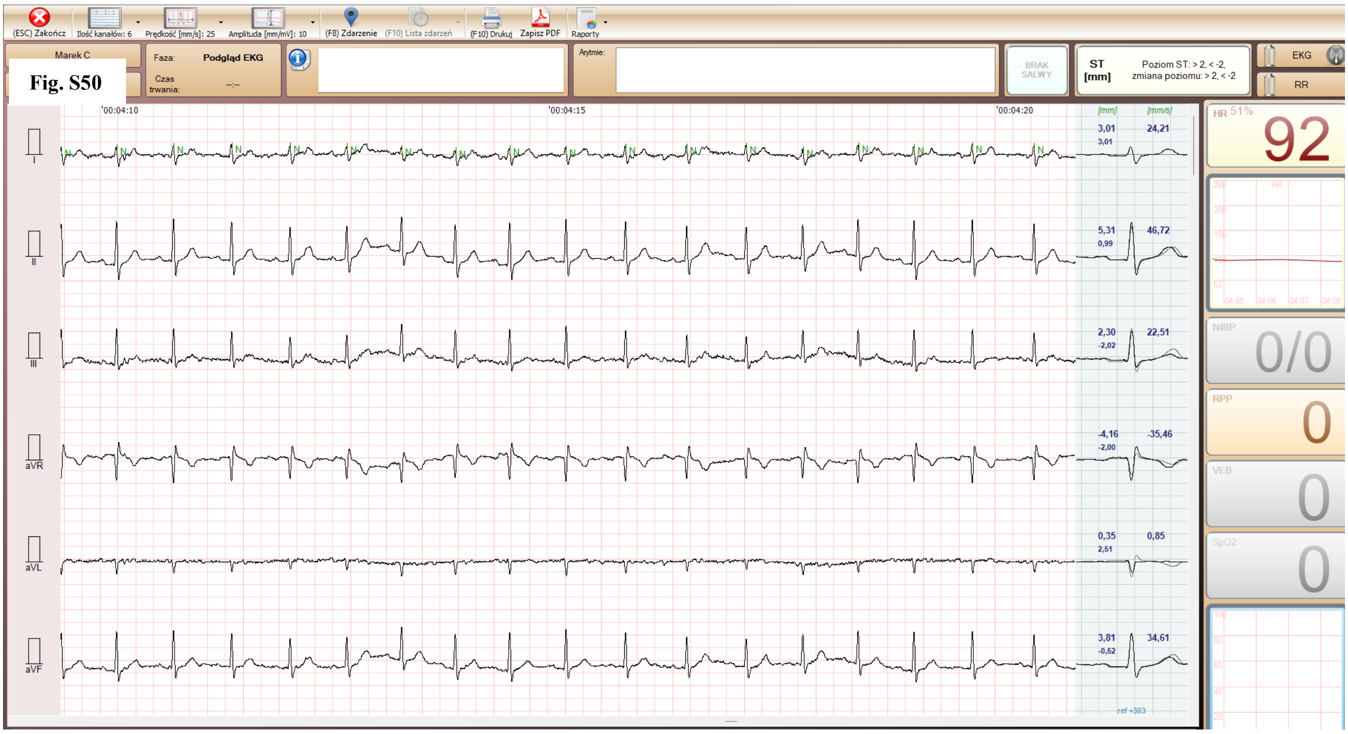

Fig. S50
